# Genetic architecture of MRI-derived cervical spinal cord morphology reveals sensory-motor axis and systemic disease associations

**DOI:** 10.1038/s41467-026-77419-x

**Published:** 2026-09-16

**Authors:** Zhuopin Sun, Jiru Han, Zachary F. Gerring, Victoria E. Jackson, Oneil G. Bhalala, Gustavo M. Jarola, Ian H. Harding, Thiago J. R. Rezende, Melanie Bahlo

**Affiliations:** 1 The Walter and Eliza Hall Institute of Medical Research, Parkville, VIC Australia; 2 Department of Medical Biology, The University of Melbourne, Parkville, VIC Australia; 3 Department of Medicine, Royal Melbourne Hospital, The University of Melbourne, Parkville, VIC Australia; 4 Department of Neurology, School of Medical Sciences, University of Campinas (UNICAMP), Campinas, São Paulo Brazil; 5 QIMR Berghofer Medical Research Institute, Brisbane, QLD Australia; 6 Department of Neuroscience, School of Translational Medicine, Monash University, Melbourne, Australia; 7 Department of Neurosciences, School of Medicine, University of São Paulo at Ribeirão Preto (USP-RP), Ribeirão Preto, SP Brazil

**Keywords:** Genome-wide association studies, Magnetic resonance imaging, Diseases

## Abstract

The spinal cord is critical to motor, sensory, autonomic function, and increasingly implicated in neurological disease and human health, yet its genetic architecture remains largely unexplored. We perform large-scale phenotyping of the upper cervical spinal cord (C1-C3) structure using brain magnetic resonance imaging from over 80,000 UK Biobank participants, extracting shape metrics including cross-sectional area, diameters, and eccentricity. We identify a total of 179 independent genome-wide significant variants, with cervical spinal cord morphology showing moderate to high SNP-based heritability (0.16 to 0.42). We also uncover sex-specific genetic signals, highlighting potential biological sex differences in spinal cord development. In addition, spinal cord structure is associated with a wide range of neurological, metabolic, and systemic conditions, such as multiple sclerosis, neuropathies, diabetes, and attention-deficit/hyperactivity disorder. These findings establish the cervical spinal cord as a genetically informative and health relevant structure, offering opportunities to study its role in disease mechanisms and human health.

## Abstract (web summary)

A genome-wide association study of cervical spinal cord morphology using UK Biobank brain MRI data identifies 179 genetic loci shaping spinal cord morphology, revealing its links to systemic health.

## Introduction

The spinal cord is the primary communication pathway between the brain and the body, enabling sensory signal transmission, motor commands, and sensory-motor integration for movement coordination and autonomic function^1^. Through sensory and motor tracts, the spinal cord connects to the brainstem, thalamus, basal ganglia, cerebellum, and motor-sensory cortices^2^. Spinal cord abnormalities are implicated in a wide range of health conditions, including multiple sclerosis (MS)^3,4^, motor neuron diseases^5,6^, spinal cord injury^7,8^, pain^9,10^, ataxia^11,12^, and other degenerative conditions. Studying the spinal cord is essential not only for defining neural organisation and sensorimotor integration, but also for understanding pathological changes that inform clinical diagnosis, symptom manifestation, and treatment.

Recent clinical and population cohorts linking imaging-derived phenotypes (IDPs) and genetic variation have provided insights into biological mechanisms underlying development and disease susceptibility. Previous genome-wide association studies (GWAS) using the UK Biobank (UKB) imaging and genetic data have explored structural and functional brain measurements^13–19^, retinal thickness^20^, skeletal proportions^21^, and other organ-specific traits^22,23^. However, while GWAS have identified hundreds of loci associated with magnetic resonance imaging (MRI) derived brain phenotypes, the genetics of the spinal cord remain largely unexplored. This gap reflects a major limitation in our understanding of the genetic basis of the full central nervous system and its relationship to health and disease.

Neuroimaging advances have enabled non-invasive characterisation of spinal cord anatomy and functions^24,25^, revealing changes associated with neurological diseases^26,27^, injury^28,29^, and biological factors^30,31^. Genetically, a small twin study^32^ suggested high heritability of cervical spinal cord structure (h^2^ = 0.85–0.91), and genotype-specific spinal cord damage has been reported in spinocerebellar ataxias^33^. These studies demonstrate the potential role of genetic variability in shaping spinal cord structure. However, existing findings have relied on modestly sized clinical cohorts. A key challenge has been the lack of reliable high-throughput methods to process spinal cord MRI at scale, due to the sensitivity of spinal cord imaging to anatomical variability, neck curvature, partial volume effects, motion, and distortion artefacts, making fully automated segmentation, labelling, and quality control difficult, especially in large scale datasets^24,34^.

To address this, we implemented a fully automated deep learning-based pipeline optimised for upper cervical spinal cord segmentation and labelling, robust to pathological variations. We applied this pipeline to the UKB T1-weighted brain MRI, extracting spinal cord shape metrics from over 80,000 participants. These IDPs capture the mean cross-sectional area (CSA), eccentricity, anterior-posterior (AP) and right-left (RL) diameters of the spinal cord at the C1, C3, and C3 vertebral levels. Leveraging these quantitative traits, we conducted a GWAS of spinal cord morphology, establishing its genetic architecture and heritability, and highlighting potential links to development and disease susceptibility. This work establishes a scalable framework for spinal cord imaging genetics and highlights the value of incorporating the spinal cord into future integrative studies of human health and disease.

## Results

### Spinal cord phenotypes

**Fig. 1:**
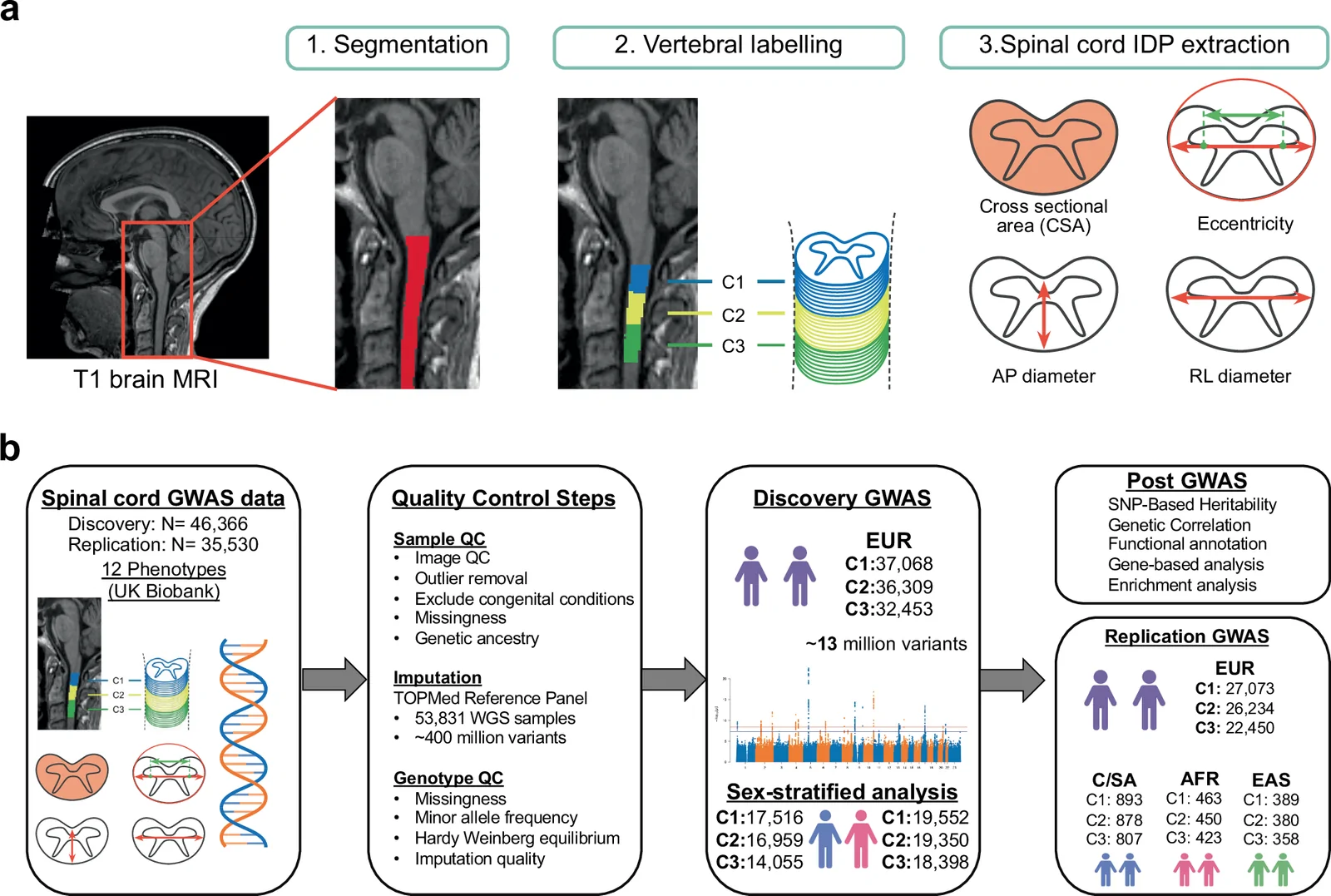
Genome-wide associations of cervical spinal cord shape metrics. **a** An overview of the spinal cord image processing pipeline using T1-weighted brain MRI from the UKB. Following spinal cord segmentation and vertebral labelling of C1 to C3, spinal cord shape metrics including the mean CSA, eccentricity, AP and RL diameters were derived. **b** Overview of the GWAS study design for spinal cord shape metrics. We conducted GWAS of 12 MRI-derived spinal cord shape phenotypes, including CSA, eccentricity, AP and RL diameters at cervical levels C1 to C3. Quality control included sample and genotype filtering, with imputation using the TOPMed reference panel. Discovery analyses were performed in individuals of European (EUR) ancestry, including sex-stratified GWAS, followed by post-GWAS analyses. Replication was carried out in EUR, Central/South Asian (C/SA), East Asian (EAS), and African (AFR) cohorts. Detailed sample and demographic information for all cohorts is provided in Supplementary Data 35. Spinal cord shape metric icons in (**a**) and (**b**) are reproduced according to Fig. 1 in Valošek et al., 2024^93^ to depict standard geometric conventions. **a** Reproduced by kind permission of UK Biobank ©.

We applied and validated a fully automated pipeline optimised for upper cervical spinal cord segmentation and phenotyping^35^ (Fig. 1a, detailed in Methods). In brief, raw T1-weighted brain MRI data underwent spinal cord segmentation using the Spinal Cord Toolbox^25^ followed by vertebral labelling of the first three cervical spinal cord segments (C1–C3) using a pretrained nnU-Net model^36^. Twelve (4 measurements × 3 spinal levels) spinal cord IDPs were then extracted, including CSA, eccentricity, AP and RL diameters of the spinal cord at each vertebral level. After quality control (QC), IDPs were available for 45,684 (C1), 44,737 (C2), and 40,024 (C3) participants in the discovery cohort (UKB release v15.1). An additional 34,969 (C1), 33,847 (C2), and 28,894 (C3) IDPs were available for the replication cohort (UKB release v20.5). The distribution of spinal cord phenotypes and their average values across age are shown in Supplementary Fig. 1-2.

Using follow-up data from repeat scans (Supplementary Section 2.1), repeatability analysis indicated that eccentricity was highly stable across C1-C3 (Spearman’s *r*^*2*^ = 0.77–0.91) (Supplementary Fig. 4), whereas CSA and diameters were more susceptible to age-related degeneration (Supplementary Fig. 2). C1 phenotypes had moderate-to-high correlation (*r*^*2*^ = 0.68–0.91) within the first four years, but had declining repeatability over longer time spans and weaker longitudinal agreement at C2 (*r*^*2*^ = 0.62–0.89) and C3 (*r*^*2*^ = 0.50–0.88) levels (Supplementary Fig. 4). At the metric extraction stage, additional robustness tests were conducted to ensure our final set of phenotype extractions was robust and independent of coordinate space (Supplementary Section 2.2). Spinal cord phenotypes at C2 and C3 showed high cross-method consistency across all traits (*r*^*2*^ = 0.97–0.99) using the subject- and atlas-based analysis. Notably, extraction in the subject space yielded better C1 detection and eliminated potential registration errors (Supplementary Fig. 6). Detailed quality control and validation are presented in the “Methods” section and Supplementary sections 2, 14.

### GWAS sample characteristics

Following sample and variant QC of the imputed genetic data (Fig. 1b and Methods), the discovery GWAS (mean age = 64 years, SD = 7.69 years, 52.6% female) focused on individuals of European ancestry, defined by the Pan-UK Biobank^37^. After QC (Supplementary Data 1, Methods), the study population included 37,068 individuals at the C1 spinal level, 36,309 at C2, and 32,453 at C3. Sex-stratified GWAS was performed separately at each spinal level. For replication, we performed GWAS in a European-ancestry cohort within the UK Biobank using additional individuals from the 2025 imaging release (v20.5), applying the same analytical framework as in the discovery analysis. The replication population included 27,073 individuals at the C1 spinal level, 26,234 at C2, and 22,450 at C3. In addition, we examined the independently significant SNPs identified in the European-ancestry GWAS among non-European individuals in the UKB, including those of Central and South Asian (C/SA), African (AFR), and East Asian (EAS) ancestry. Sample sizes for each group are shown in Fig. 1b.

### GWAS identifies 179 independent genome-wide significant variants

**Fig. 2:**
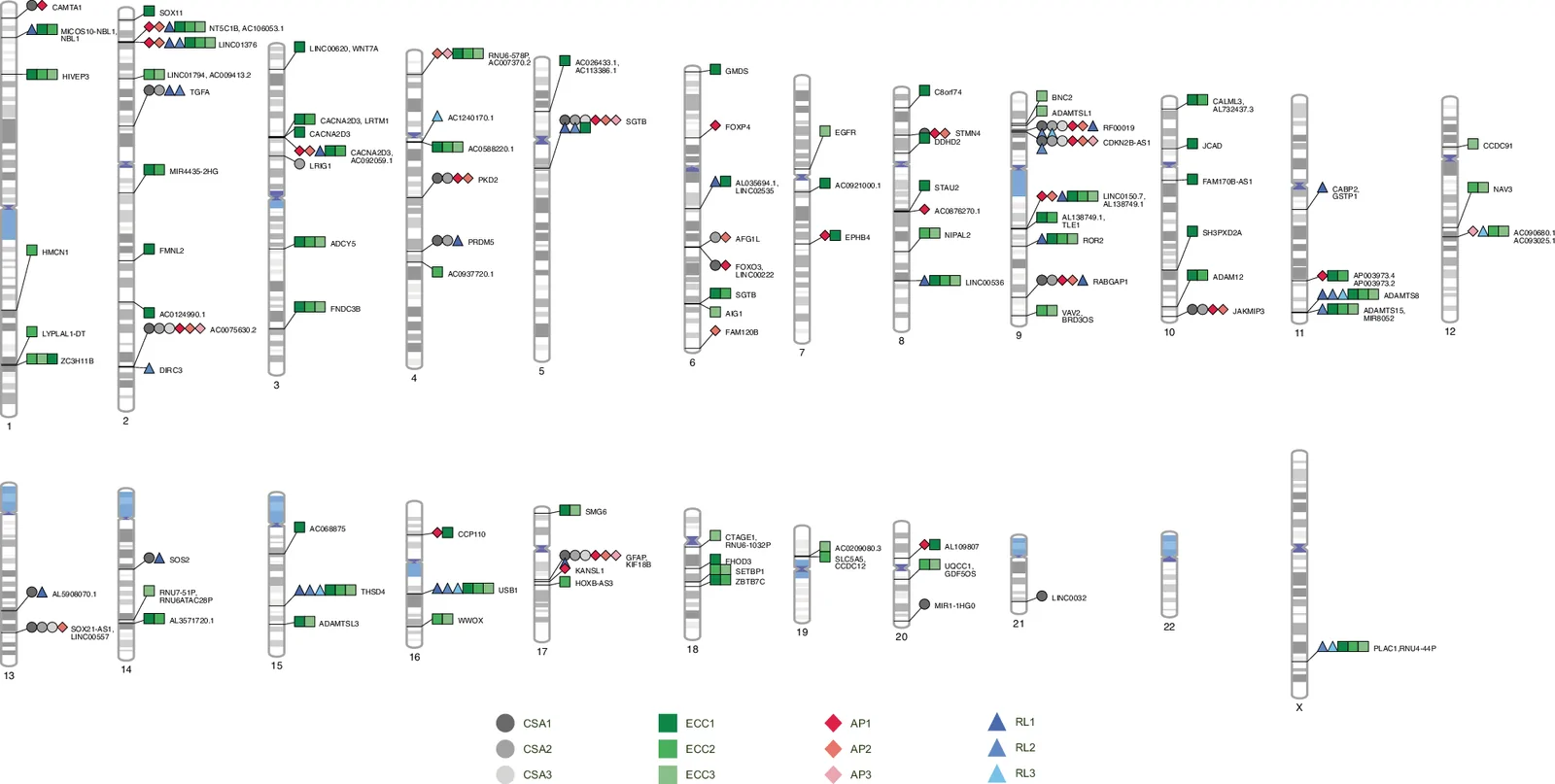
Ideogram of genomic loci associated with 12 spinal cord shape phenotypes. Shapes represent the four spinal cord shape metrics: cross-sectional area CSA, eccentricity ECC, anterior-posterior AP and right-left RL diameters, and are shaded by vertebral level (C1 to C3). Loci were defined by merging genome-wide independent significant variants (*P* < 4.17 × 10⁻⁹, REGENIE; Bonferroni-corrected for 12 phenotypes) that were in linkage disequilibrium (r²  > 0.1) and located within 250 kb, based on the discovery GWAS in individuals of European ancestry (C1: *n* = 37,068; C2: *n* = 36,309; C3: *n* = 32,453). Each locus is labelled with the nearest protein-coding gene, and loci annotated to the same gene were merged for clarity. Complete results of all independent significant variants are presented in Supplementary Data 2.

We conducted quantitative trait GWAS for all autosomes and the X chromosome for the 12 spinal cord IDPs using REGENIE^38^, adjusting for sex, age, age², genotyping batch, imaging site, intracranial volume (ICV), height, and the inverted signal-to-noise ratio in T1-weighted images. We identified 179 independent genome-wide significant variants (*P* < 4.2 × 10⁻⁹, adjusted for 12 phenotypes) mapping to 104 unique genomic loci, including one on the X chromosome (Fig. 2 and Supplementary Data 2). Manhattan and quantile–quantile (Q–Q) plots are in Supplementary Fig. 9–12. Genetic inflation factors (*λ* = 1.04–1.09) and LDSC intercept values (0.99–1.03) suggest little to no inflation due to population stratification, or confounding factors (Supplementary Data 3). Eccentricity showed the most associations: 58 variants at C1, 46 at C2, and 35 at C3 (78 total, 13 shared across C1–C3). Other phenotypes showed fewer associations: AP diameters (22, 17, 6 variants at C1, C2, C3; 27 total, 4 shared), RL diameters (22, 11, 7 variants; 27 total, 5 shared), and CSA (18, 13, 6 variants; 20 total, 6 shared).

Among the 104 genomic loci associated with spinal cord IDPs, several were shared across multiple phenotypes and/or across spinal levels (Supplementary Fig. 13). A locus on chromosome 5, spanning a gene cluster that includes *ADAMTS6*, *CENPK*, *TRAPPC13*, and *SGTB*, was associated with all four spinal cord shape metrics. The most statistically significant association at this locus was observed for rs201934842 in the 3′ UTR of *SGTB*, with C1 CSA (Minor allele frequency (MAF) = 0.26, *beta*  =  0.079, *P*  =  2.65 × 10⁻²³). *SGTB* has been linked to brainstem volume and synaptic protein homoeostasis^39^. A few rare (MAF < 0.01) SNPs exhibited relatively large effect sizes (Supplementary Fig. 14). For example, rs554265256 (MAF = 0.0083), located in an intronic region of *RABGAP1*, was associated with CSA, AP, and RL diameters (peak association with CSA C1: *beta* = −0.29, *P* = 6.5 × 10⁻¹⁴). This variant has been linked to iron levels in the brain^40^. RABGAP1, a GTPase-activating protein involved in vesicular trafficking and cell migration, is linked to neurodevelopmental syndromes^41^ and potentially neurodegenerative disease^42^.

Cross-referencing the 179 independent genome-wide significant variants with the GWAS Catalog^43^ revealed 50 variants previously associated with 120 unique phenotypes (Supplementary Data 4). The most frequent associations were with brain-related traits, blood pressure measures, and body composition, suggesting enrichment in neurological, cardiovascular, and anthropometric phenotypes.

Height was included as an important covariate in the main GWAS due to its significant correlation with brain volume and spinal cord^30^, confirmed by stepwise covariate selection analysis (Supplementary Fig. 15). For sensitivity analysis, we performed GWAS without adjusting for height (Supplementary Fig. 16–19, Supplementary Data 3). We identified 182 independent genome-wide significant variants (*P* < 4.2 × 10⁻⁹), mapping to 104 unique genomic loci (Supplementary Data 5). Most loci (100/104) were consistently identified in both height-adjusted and non-adjusted analyses, indicating robust associations independent of height. However, three loci (rs5861819, rs2956033, rs10883922) were significant only in the height-adjusted analysis, while four loci (rs58273475, rs112635299, rs2410978, rs4128399) were significant only in the non-adjusted analysis, suggesting that a small subset of loci may reflect height-mediated or height-independent effects. In addition, SNP effect sizes showed high concordance in both direction and magnitude, with Pearson correlation coefficients ranging from 0.986 to 0.999 when comparing results with and without height adjustment (Supplementary Data 6). *P* values were also highly correlated (*r* = 0.944–0.996).

### Heritability and genetic correlation of spinal cord shape metrics

**Fig. 3:**
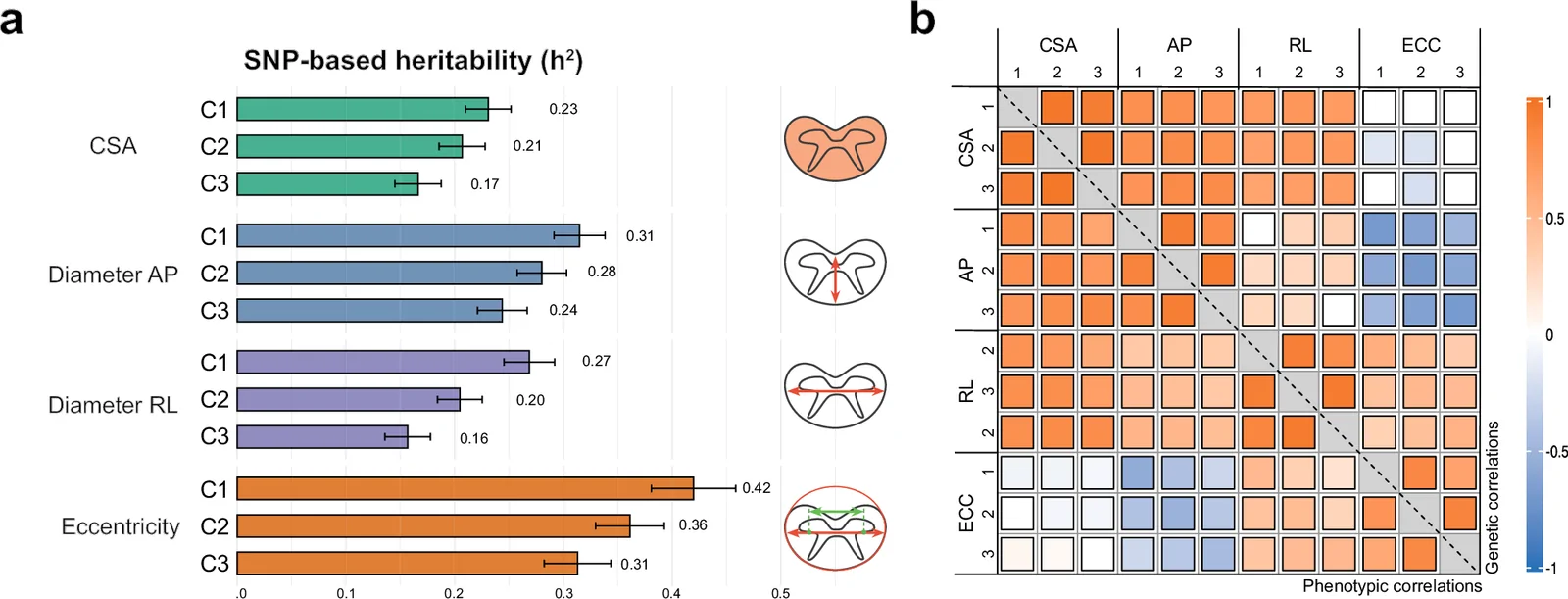
Heritability estimation and correlations of spinal cord shape metrics. **a** SNP-based heritability (h^2^) and (**b**) phenotypic (lower half of the heatmap) and genetic correlations (upper half of the heatmap) of the spinal cord shape metrics (LDSC). Sample sizes used are 37,068 (C1), 36,309 (C2), 32,453 (C3) to calculate SNP-based heritability and genetic correlation. Error bars in (a) represent standard error. Correlations shown in (**b**) reached statistical significance after Bonferroni correction for multiple comparisons; non-significant correlations are set to 0 in the heatmap. Spinal cord shape metric icons in (**a**) are reproduced based on Spinal Cord Toolbox documentation²^5^. AP: Anterior-Posterior; RL: Right-Left; CSA: cross sectional area; ECC: eccentricity. The complete heritability estimates are provided in Supplementary Data 7; phenotypic and genetic correlation results are provided in Supplementary Data 8 and 9.

SNP-based heritability for spinal cord shape metrics across the C1, C2, and C3 levels was estimated using linkage disequilibrium score regression (LDSC)^44^. Heritability estimates ranged from 0.16 to 0.42, indicating moderate to strong genetic contribution (Fig. 3a and Supplementary Data 7). Eccentricity showed the highest heritability overall, with an estimate of 0.42 at C1, 0.36 at C2, and 0.31 at C3. We observed a consistent trend of decreasing heritability from the C1 to C3 levels across all traits. This pattern was consistently observed in analyses of the discovery cohort restricted to the same individuals across segments, and was also replicated in both the replication cohort and its corresponding same-sample analyses (Supplementary Fig. 20).

Genetic correlations among the 12 spinal cord phenotypes were examined using LDSC to assess the degree of shared genetic architecture (Fig. 3b and Supplementary Data 8). CSA showed strong genetic correlations across all spinal levels (C1-C2: *r*_g_ = 0.98, C2-C3: *r*_g_ = 0.98, C1-C3: *r*_g_ = 0.94), indicating consistent genetic influence. AP and RL diameters also exhibited strong inter-level correlations ranging from 0.93 to 0.96. In contrast, eccentricity showed moderate within-phenotype correlations (C1-C2: *r*_g_ = 0.87; C1-C3: *r*_g_ = 0.67), suggesting partially distinct genetic determinants across levels.

Between phenotypes, CSA strongly correlated with AP and RL diameters (*r*_g_ = 0.72–0.80), as expected given their geometric relationship. Eccentricity, derived from the ratio of RL and AP diameters, negatively correlated with AP diameter (peak *r*_g_ = –0.69) and positively with RL diameter (peak *r*_g_ = 0.58). In contrast, CSA and eccentricity were weakly correlated, suggesting that they likely capture distinct biological dimensions.

The overall pattern of spinal cord shape metrics phenotypic correlations closely mirrored the genetic correlations (Fig. 3b and Supplementary Data 9).

### Sex-specific genetic associations

We investigated potential sex differences in the genetic architecture of spinal cord shape by conducting sex-specific GWAS for each of the 12 traits. Miami plots of the sex-stratified GWAS results are in Supplementary Fig. 21–24. Genomic inflation factors ranged 1.02–1.06 in females and 1.00–1.05 in males, with LDSC intercepts of 0.99–1.01 in both sexes (Supplementary Data 3). SNP-based heritability estimates ranged 0.17–0.38 in females and 0.10–0.43 in males, similar to sex-combined GWAS (Supplementary Data 7). We further assessed the concordance between sexes and observed a strong correlation in SNP-based heritability estimates across the 12 traits (Pearson correlation coefficients *r* = 0.887, *P* = 0.00012; Supplementary Fig. 25).

We identified two independent genetic signals (*P* < 4.17 × 10⁻⁹, Bonferroni-corrected for 12 traits) that were not detected through our sex-combined analyses. Rs150219059, an intronic SNP in *PEPD*, was significantly associated with C2 AP diameter in males (MAF = 0.045, *beta* = 0.15, *P* = 1.94 × 10⁻⁹), but not females (MAF = 0.047, *beta* = 0.015, *P* = 0.52). Similarly, rs373400435 (intergenic) was associated with C1 AP diameter in males only (males: MAF = 0.0028, *beta* = 0.6, *P* = 1.22 × 10⁻⁹, females: MAF = 0.0029, *beta* = 0.057, *P* = 0.54). SNP by sex interaction analysis confirmed a significant sex-interaction for these two SNPs (Supplementary Data 10, Supplementary Fig. 26).

SNP-by-sex interactions were additionally examined for all independent SNPs identified through the sex-combined discovery analyses. Rs2328459, associated with C3 eccentricity, showed significant interaction (*P* = 9.65 × 10⁻^6^, Benjamini-Hochberg *P* = 0.0026, Supplementary Data 10). Sex-specific analyses revealed significant association in females (MAF = 0.54, *beta* = −0.069, *P* = 1.1 × 10⁻^11^, Supplementary Fig. 26), but not males (MAF = 0.54, *beta* = −0.012, *P* = 0.31). This SNP is within an intron of *AIG1* (Androgen Induced 1), involved in fatty acid metabolism and may be regulated by androgen levels.

### Associations in replication GWAS

We performed a separate replication GWAS (*n* = 22,450 to 27,073) for each of the 12 spinal cord shape traits. Manhattan and quantile–quantile (Q–Q) plots are shown in Supplementary Fig. 27–30. Genomic inflation factors ranged from 1.03–1.06, with LDSC intercepts of 1.00–1.03 for the replication GWASs. The discovery and replication GWAS results demonstrated strong genetic correlations across all phenotypes (*r*_g_ = 1.00–1.12; all *P* < 5.5 × 10⁻^22^; Supplementary Data 11). Each of the 179 independent genome-wide significant loci identified in the discovery cohort were evaluated in the replication GWAS with detailed discovery, replication, and combined meta-analysis statistics provided in Supplementary Data 12. All variants showed concordant directions of effect across phenotypes (Supplementary Fig. 31) in the replication cohort. Across all phenotypes, most variants ( ≥ 84.6%) were nominally significant (*P* < 0.05) in the replication cohort (Supplementary Data 13). A substantial proportion of loci also achieved genome-wide significance, with at least 28.6% of variants reaching *P* < 5 × 10⁻^8^. These results support the reproducibility of the identified loci and reinforce the robustness of our findings.

### Association analyses in non-European ancestries

Replication analyses of 179 independent genome-wide significant loci from the European-ancestry discovery GWAS were performed using non-European UKB cohorts: C/SA (*n* = 893–807), AFR (*n* = 463–423), and EAS (*n* = 389–358). After excluding SNPs with MAF < 0.05, 178, 175, and 178 variants were available in C/SA, EAS, and AFR cohorts, respectively. No variants reached Bonferroni-corrected significance (*P* < 0.00028, correcting for 179 tests) in any non-European group. Effect size estimates were compared between European and non-European ancestry groups (Supplementary Fig. 32–34). Directional concordance ranged from 38.5% (C2 CSA in AFR) to 100% (C3 RL diameter in C/SA and C3 AP diameter in AFR), with overall higher concordance observed in C/SA compared to EAS and AFR. Spearman correlation coefficients between effect sizes were generally positive but mostly not statistically significant. These results suggest that many loci exhibit consistent effect directions across ancestries, particularly in C/SA cohort. However, the lack of statistical significance and the variability in correlation strength reflect the limited power of the currently available non-European samples.

### Gene-based findings

Candidate risk genes for each of the four morphological features of cervical spinal cord segments C1–C3 were prioritised using six gene-based mapping approaches. Positional mapping was conducted with MAGMA and mBAT-combo, while functional mapping leveraged eQTL data via TWAS (PsychENCODE and GTEx v8 expression weights) and SMR (eQTLGen and BrainMeta datasets). Across all spinal cord morphology-segment combinations, we identified 432 unique significant genes after applying a Bonferroni correction (*P* < 0.05 after adjusting for all tests within each gene-based method and phenotype; for the multi-tissue TWAS and SMR panels, the denominator is the number of gene × tissue tests, and the most significant tissue per gene was selected only after correction). The largest number of unique gene associations was found for eccentricity (*n* = 241), followed by AP diameter (*n* = 194), CSA (*n* = 165), and RL diameter (*n* = 113). The proximity-based approaches mBAT-combo and MAGMA identified the largest number of significant genes, with 292 and 196 genes, respectively, followed by TWAS of GTEx tissues with 128 significant gene associations (Supplementary Data 14–30). Of the 442 genes significantly associated with a spinal cord phenotype in at least one approach, 218 were implicated by at least two approaches and 96 by at least 3 approaches (Supplementary Data 18, 19). Five genes—*DPYSL4*, *CCDC88B*, *UQCC1*, *UFSP1* and *DDHD2*—were significant in all six approaches.

**Fig. 4:**
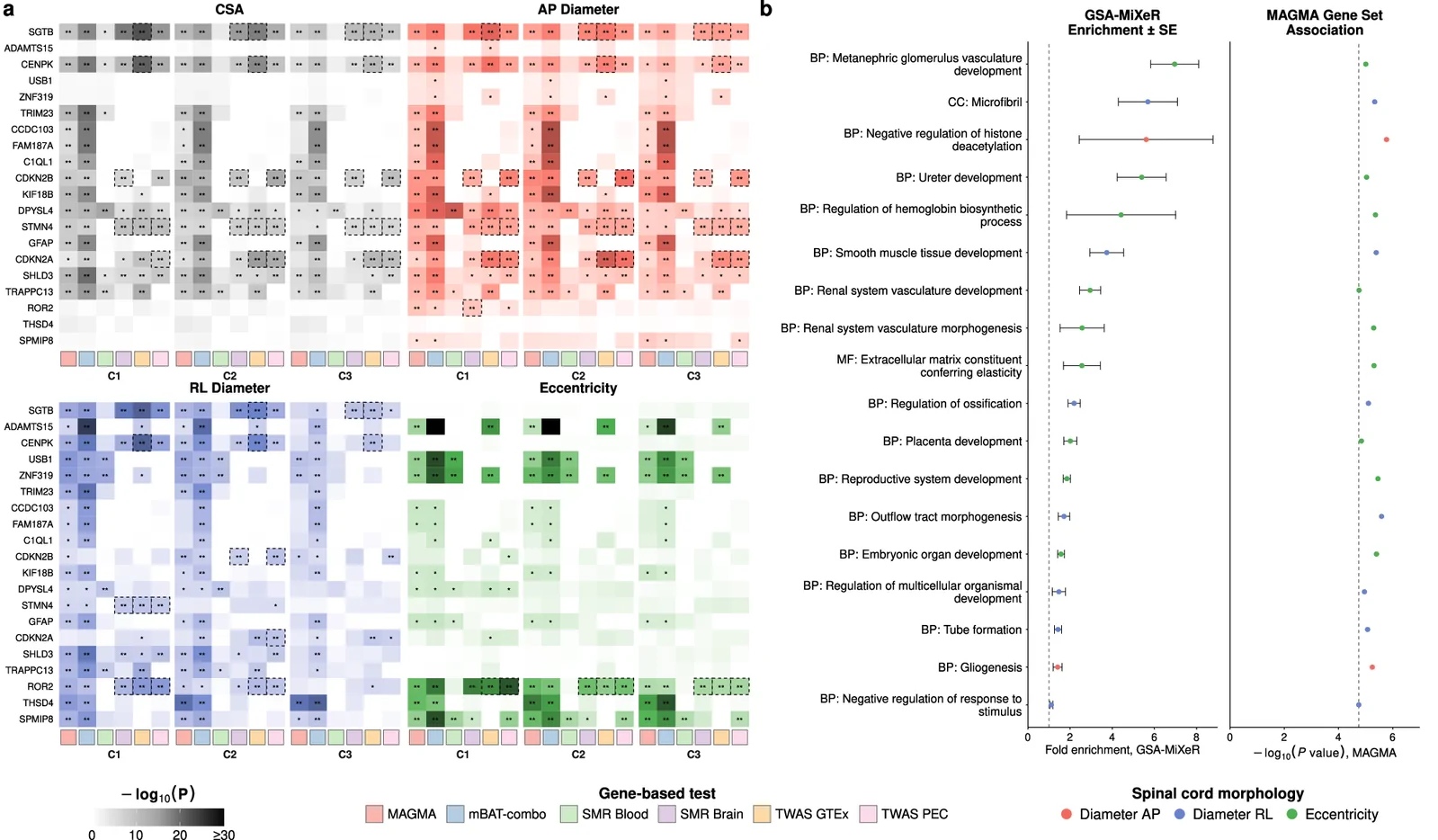
Top 20 prioritised gene-based associations for spinal cord morphology phenotypes. **a** For each combination of spinal cord vertebra (C1, C2, C3) and spinal cord morphology phenotype (CSA, AP diameter, RL diameter, and eccentricity), we extracted gene-level association results from six gene-based analysis methods: MAGMA, mBAT, SMR (BrainMeta and eQTLgen), and TWAS (GTEx and PsychENCODE). For each method and verbetra-morphology feature pair, we identified the gene with the minimum *p* value. From these results, a set of unique gene identifiers was extracted to summarise the most significant associations across all analytic approaches and phenotype combinations. The genes are ordered by the number of times they were significant after Bonferroni correction across all verbetra-morphology feature pair combinations. * FDR < 0.05; ** Bonferroni *P* < 0.05; dashed boxes represent significant gene-based associations (Bonferroni *P* < 0.05) with evidence the same causal variant underlies the GWAS and eQTL association using the HEIDI test in SMR (HEIDI P > 0.05) or colocalisation in TWAS (COLOC PP.H4 > 0.8). **b** Gene sets prioritised by MAGMA, ranked by GSA-MiXeR fold enrichment. GSA-MiXeR fold enrichment estimates with standard errors; the vertical dashed line denotes enrichment threshold (fold enrichment = 1). MAGMA -log(10) *P* values gene set analysis; the vertical dashed line indicates the Bonferroni-corrected significance threshold. GOMF: Gene Ontology Molecular Function; GOBP: Gene Ontology Biological Process; GOCC: Gene Ontology Cellular Component.

Among the 96 genes identified by at least three mapping approaches, gene candidates for spinal cord phenotypes were further prioritised using colocalization (TWAS-COLOC) and SMR-HEIDI analyses (Fig. 4a and Supplementary Data 21, Supplementary Fig. 35). Colocalization identified TWAS associations supported by a shared causal variant between GWAS and eQTL signals (posterior probability H4 > 0.8). HEIDI was used to retain SMR associations consistent with a single causal variant influencing both gene expression and trait (*P* > 0.05). Of the 96 genes implicated by at least three gene-based tests, 44 had a significant TWAS association in GTEx brain tissue and showed evidence of colocalization (i.e., PP.H4 > 0.8), while 41 genes had a significant SMR association in brain tissue, 29 of which had a HEIDI *P* value > 0.05. Top among the list of high confidence genes was *SGTB*, which was implicated by five gene-based approaches across three morphological measures and showed evidence of colocalization in human brain tissue. Other high confidence genes included *TGFA* and *ROR2*, both of which were implicated by five gene-based approaches (across three spinal levels) and colocalized in human brain tissue (Fig. 4a). Although *TGFA* fell just outside the top 20 when genes were ranked jointly across all features and tests (rank 24 of 1761 genes), it was among the most highly ranked genes for individual features—8th for RL diameter and 17th for CSA—and is nonetheless highlighted here as a candidate of interest. These candidate genes have important implications in diverse functions such as neuronal survival and neurite morphology^45^, neural tissue proliferation during development^46^, and axial skeletal and vertebral patterning^47^.

### Gene set enrichment analysis

We applied GSA-MiXeR and MAGMA gene-set enrichment analysis to each spinal cord morphology phenotype (Fig. 4b and Supplementary Data 31). Among the 20 significant (*P*_*FDR*_ < 0.05) MAGMA gene sets, gene sets with the largest GSA-MiXeR enrichment included pathways related to organ development (e.g., metanephric glomerulus vasculature [GSA-MiXeR enrichment = 6.96; MAGMA *P* = 9.82 × 10⁻^6^] and smooth muscle tissue development [enrichment = 3.74; MAGMA *P* = 4.06 × 10⁻^6^]). We also identified more specific biological pathways related to spinal cord development and morphology, including microfibril pathways (enrichment = 5.69; MAGMA *P* = 4.61 × 10⁻^6^), tube formation (enrichment;= 1.43; MAGMA *P* = 8.43 × 10⁻^6^), and gliogenesis (enrichment = 1.41; MAGMA *P* = 5.65 × 10⁻^6^). We found 37 overlapping genes between our prioritised list of candidate genes (Supplementary Data 19) and gene members of enriched gene sets. The gene overlap showed that the enrichment of organ development pathways is driven by a core set of significant genes from our gene-based analysis (*OSR1*, *BMP7*, *PKD2*, *WNT9B*, and *PDGRFA*) that are significant in at least one of the spinal cord phenotypes tested.

### Associations with brain-image derived phenotypes

**Fig. 5:**
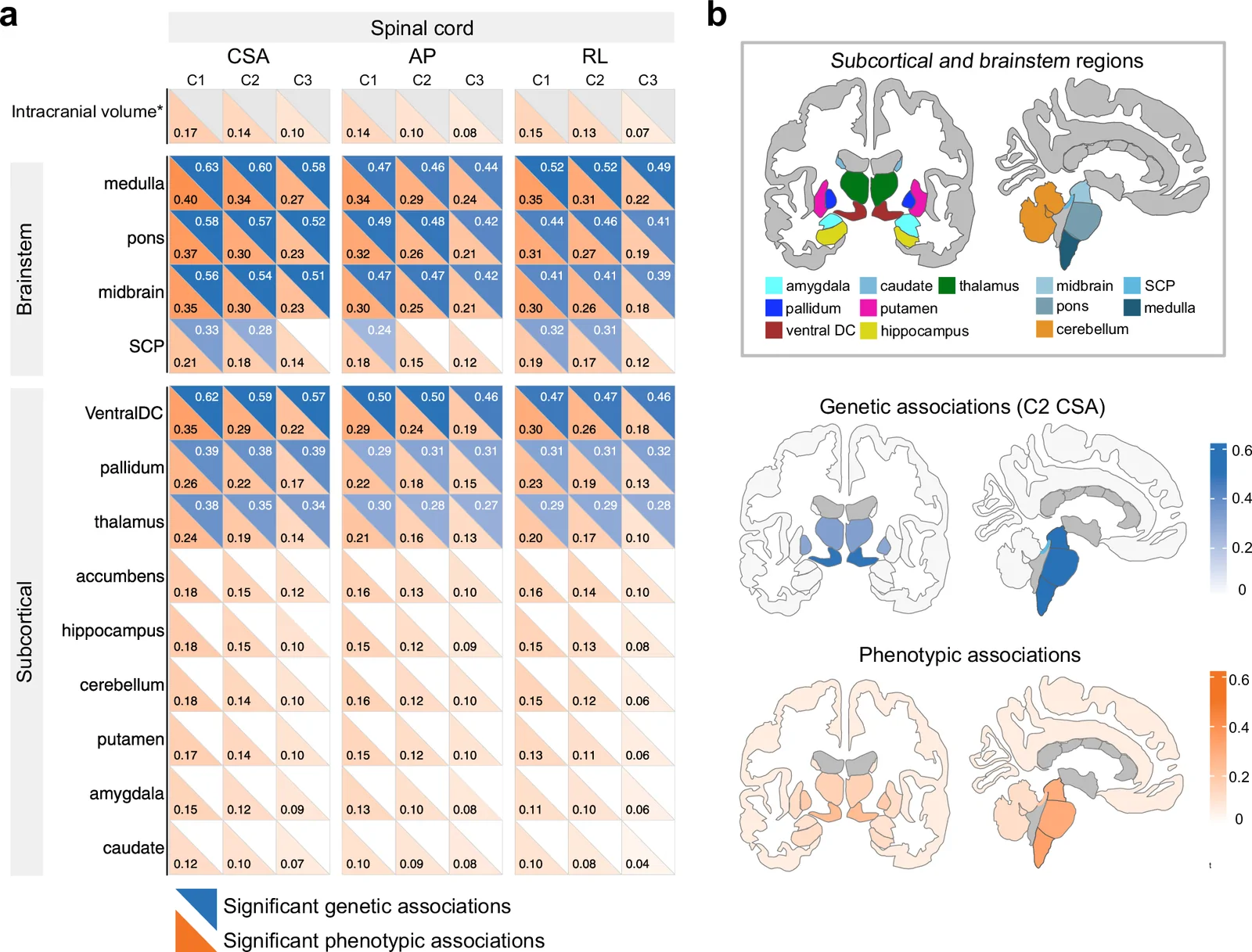
Genetic and phenotypic associations between spinal cord phenotypes and brain volumes. **a** Heatmap of significant associations (two-sided, Bonferroni-corrected *P* < 0.05/504, correcting for 42 brain regions × 12 spinal cord phenotypes) sorted by effect size: upper blue triangle shows genetic correlations (r_g_), lower orange triangle shows phenotypic associations (beta). Eccentricity and cortical regions are not displayed due to no significant genetic associations. Genetic correlation for intracranial volume is not calculated as it’s used as a covariate in GWAS. **b** top row: subcortical and brainstem regions in Freesurfer aseg atlas, generated using the ggseg R package^94^; middle: significant genetic associations for C2 cross-sectional area (CSA); bottom: phenotypic associations for C2 CSA. SCP: Superior Cerebellar Peduncle; ventral DC: ventral diencephalon. AP: anterior-posterior; RL: right-left. Colour intensity indicates magnitude of the correlations/beta values. Complete results of all genetic and phenotypic associations with brain volumes are presented in Supplementary Data 32-33.

We examined genetic correlations and phenotypic associations between spinal cord phenotypes and brain volumes (Fig. 5 and Supplementary Data 32, 33). Both revealed that spinal cord CSA and diameters were most strongly associated with the brainstem, particularly the medulla (peak association with C1 CSA: *beta* = 0.40, *P* < 5 × 10⁻^324^; genetic correlation *r*_g_ = 0.63), followed by the pons and midbrain, with similar trends. C2 and C3 spinal levels showed slightly lower, but comparable correlations to C1.

Significant phenotypic associations were also observed between spinal cord CSA and diameters, and all sub-cortical regions of the brain. Among subcortical structures, the ventral diencephalon (C1 CSA *beta* = 0.35, *P* < 5 × 10⁻^324^; *r*_g_ = 0.62), pallidum (*beta* = 0.26, *P* < 5 × 10⁻^324^; *r*_g_ = 0.39), and thalamus (*beta* = 0.24, *P* < 5 × 10⁻^324^; *r*_g_ = 0.38) exhibited the strongest associations, as well as significant genetic correlations. Several additional subcortical regions showed weaker genetic correlations including the cerebellum, hippocampus, accumbens, amygdala, and putamen (Supplementary Fig. 36 and Supplementary Data 32), but were not significant after multiple testing corrections.

In cortical regions, the strongest phenotypic associations were observed in motor areas, particularly the bilateral anterior and posterior Brodmann area (BA) 4 (motor) regions (Supplementary Fig. 36 and Supplementary Data 33). Genetically, the right posterior BA4 showed the highest correlation but was not significant after multiple testing corrections. As expected, eccentricity, being a shape metric, did not show strong correlations with other brain volumes.

### Links to disease and health-related traits

**Fig. 6:**
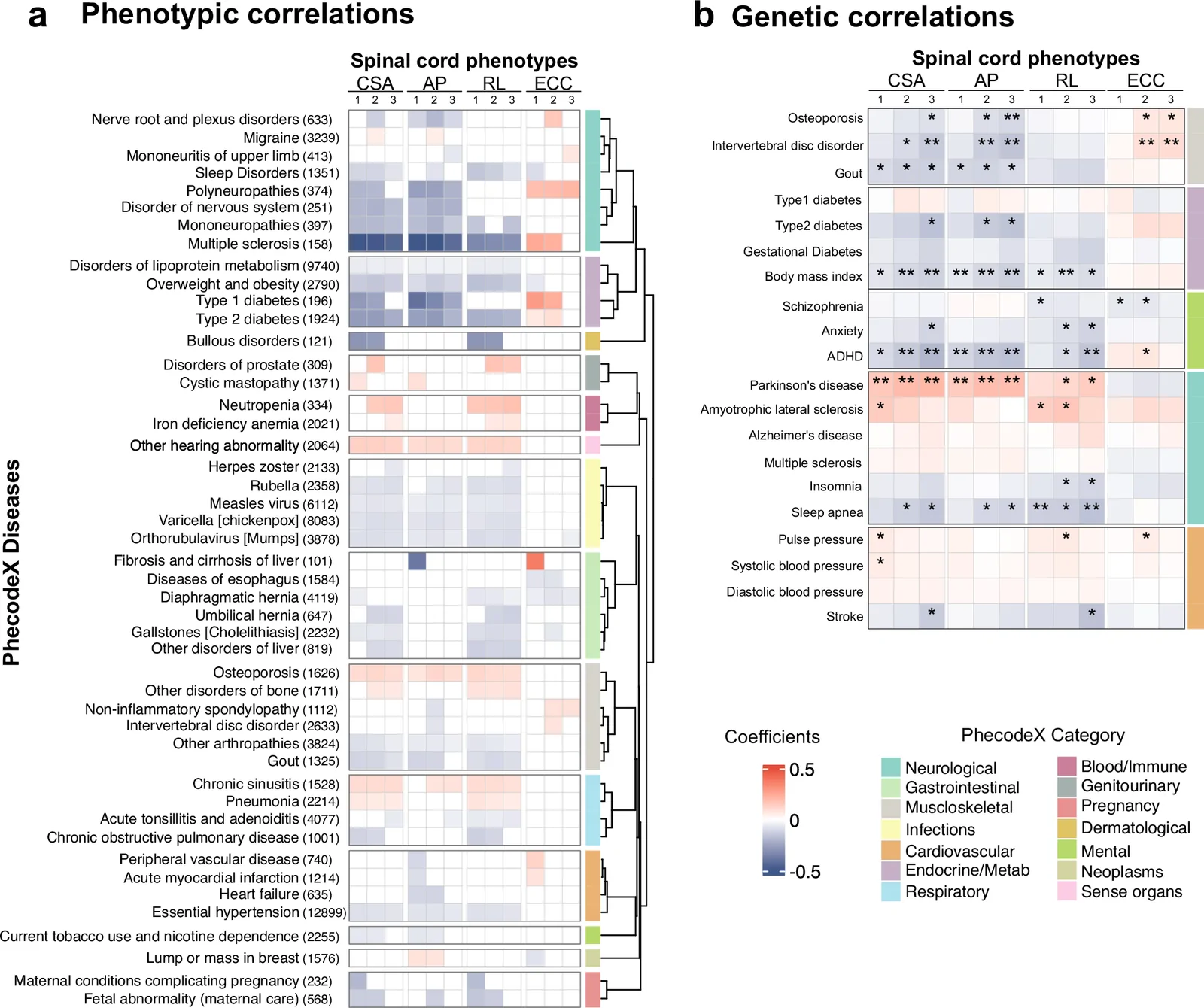
Associations between spinal cord phenotypes and health-related outcomes. **a** Heatmap of phenotypic associations (two-sided, logistic regression) between spinal cord shape metrics and health-related outcomes (converted to PhecodeX diseases) in the UKB. Only associations with FDR-adjusted *P*  <  0.05 and diseases associated with multiple spinal cord phenotype are displayed. The number of cases available for each disease is shown in parentheses next to the disease name. Diseases are grouped by category (legend on the right) and ordered by hierarchical clustering based on similarity in association patterns across 12 phenotypes. **b** Genetic associations (LDSC) for diseases/ traits. Asterisks indicate significance: * *P*  <  0.05 (nominal); **FDR-adjusted *P*  <  0.05. Blue indicates negative effect size; red indicates positive effect size, where effect size is beta for phenotypic associations and r_g_ for genetic correlations. ADHD: attention-deficit/hyperactivity disorder; CSA: cross-sectional area; AP: anterior-posterior diameter; RL: right-left diameter; ECC: eccentricity. Complete results of all phenotypic and genetic association analyses are presented in Supplementary Data 34 and 37.

We tested associations between spinal cord shape metrics and PhecodeX diseases from UKB health records, identifying significant phenotypic associations across 14 major disease categories (Fig. 6a and Supplementary Data 34). Neurological diseases showed the most significant associations, with smaller spinal cord sizes, measured by reduced CSA and diameters, consistently associated with increased risk of neurological conditions. Strongest negative associations were observed in MS (highest *beta* = -0.55, *P* = 1.8 × 10⁻^14^ for C1 AP diameter), polyneuropathies and mononeuropathies. Weaker, but notable associations were also observed with nerve root disorders and sleep disorders. In some conditions, such as polyneuropathies, increased eccentricity, reflecting flatter spinal cord, was also associated with higher disease risk.

Beyond neurological disorders, we observed strong and consistent negative associations between spinal cord size and metabolic conditions, including type 1 (highest *beta* = -0.4, *P* = 3.1 × 10⁻^10^ for C1 AP diameter) and type 2 diabetes (highest *beta* = -0.28, *P* = 8.3 × 10⁻^37^ for C1 CSA). Additional associations were identified in categories such as viral infections and musculoskeletal conditions. These findings suggest that spinal cord morphology may reflect broader systemic health and disease susceptibility.

### Polygenic Risk Scores (PRS) disease associations

For the 47 diseases showing significant phenotypic associations with spinal cord shape metrics, we aimed to emulate these associations with genetically predicted spinal cord measures in the independent remainder of the UKB. Demographic and clinical prevalence of these cohorts are shown in Supplementary Data 35. We estimated PRS for each of the 12 spinal cord measures in the remaining UKB participants without imaging data and tested these scores for disease association (Supplementary Fig. 37). Across all tested disease-spinal cord phenotype pairs, 63.2% of associations show consistent direction of effect in the PRS association analyses, compared to the phenotypic associations. After restricting to the diseases that were significant in the PRS association analyses after FDR correction, 96.1% of the tests showed concordant effects (Supplementary Fig. 38 and Supplementary Data 36). The significant but discordant associations were limited to viral infection diseases, reflecting the differential ascertainment of self-reported viral infections between imaged and non-imaged participants (Supplementary Fig. 39). In total, 11 out of 47 diseases had PRS reproducing the significant phenotypic associations with concordant effect directions. These results indicate that spinal cord PRS serve as effective proxies, validating a subset of the disease relationships identified in the phenotypic associations.

### Genetic correlations and Mendelian randomisation

Following the exploratory phenotypic associations with health-related outcomes, we used bivariate LDSC to estimate genetic correlations between spinal cord shape metrics and 20 disease traits from well-powered GWAS studies spanning neurological, musculoskeletal, metabolic, cardiovascular, and mental health conditions (Fig. 6b, Supplementary Data 37). Significant genetic correlations (*P*_*FDR*_ < 0.05) were found with intervertebral disc disorder, osteoporosis, BMI, Attention-deficit/hyperactivity disorder (ADHD), Parkinson’s disease (PD), and sleep apnoea. The genetic correlations with greatest magnitude were with PD and ADHD. PD showed positive genetic correlation with spinal cord sizes (C2 CSA: *r*_g_ = 0.20, *P* = 3 × 10⁻^4^, *P*_*FDR*_ = 6.55 × 10⁻³; C2 AP diameter: *r*_g_ = 0.18, *P* = 3 × 10⁻^4^, *P*_*FDR*_ = 6.55 × 10⁻³). ADHD had significant negative genetic correlations (C3 CSA: *r*_g_ = –0.2, *P* = 1 × 10⁻^4^, *P*_*FDR*_ = 6 × 10⁻³; C3 AP diameter: *r*_g_ = –0.17, *P* = 4.09 × 10⁻^5^, *P*_*FDR*_ = 3.27 × 10⁻³; and C3 RL diameter: *r*_g_ = –0.16, *P* = 0.002, *P*_*FDR*_ = 0.03). These results suggest potential shared genetic architecture between spinal cord shape metrics and a range of disease categories.

For traits showing significant genetic correlations with spinal cord phenotypes, and with sufficient genetic instruments ( ≥ 3 variants; BMI, PD, ADHD, sleep apnoea and osteoporosis), we next utilised Mendelian randomisation (MR) methods to explore potential causal effects of these traits on spinal cord shape metrics. Our primary MR analyses utilised the two-stage, inverse-variance weighted (IVW) approach^48^, and identified potential causal effects of PD on CSA (peak association at C3: *beta* = 0.037, *P* = 0.0054, *P*_*FDR*_ = 0.017) and AP diameter (C3: *beta* = 0.043, *P* = 0.0048, *P*_*FDR*_ = 0.017), and of BMI on CSA (C2: *beta* = -0.12, *P* = 0.0001, *P*_*FDR*_ = 0.001), AP diameter (C2: *beta* = -0.15, *P* = 4.27 × 10⁻^6^, *P*_*FDR*_ = 8.11 × 10⁻^5^), and RL diameter (C3: *beta* = -0.09, *P* = 0.0025, *P*_*FDR*_ = 0.012) (Supplementary Data 38 and Supplementary Fig. 40).

To evaluate result robustness, we performed additional analyses using complementary MR approaches (Supplementary Data 39, Supplementary Fig. 40). For BMI, similar causal estimates were obtained using weighted median and weighted mode methods; however, MR-Egger^49^ and CAUSE^50^ approaches showed no evidence of causal effect. We observed significant heterogeneity in the effects of the genetic instruments (Cochran’s Q statistic *P* values all ≤3.45 × 10^-10^). Furthermore, reverse MR revealed an apparent causal effect of CSA and AP diameter on BMI, with a direction of effect opposite to that observed in the forward MR (Supplementary Data 40). Together, these results suggest potential violations of MR assumptions, such as horizontal pleiotropy. For PD, no alternate approach provided evidence of a causal effect on spinal cord phenotypes. Overall, the inconsistencies across methods suggest that the original IVW results may not be valid due to violations of MR assumptions. Thus, our results do not provide robust evidence of a causal effect of PD or BMI on spinal cord phenotypes.

## Discussion

This study presents a GWAS of spinal cord morphology by extracting spinal cord shape metrics from MRI data. We identified novel genetic loci linked to spinal cord morphological traits and observed sex-specific associations. Our findings indicated that the spinal cord sizes have significant genetic overlap with brainstem and subcortical volumes, including the ventral diencephalon, pallidum, and thalamus. Leveraging health-related outcomes, we provide evidence that the spinal cord does not just reflect neurological health but also metabolic and wide-ranging systemic disease conditions, highlighting its importance as the conduit of nerve signals and its multifactorial vulnerability.

Our approach represents a substantial advance on previous work that quantified cervical spinal cord structure in modest sample sizes, including a small subset of UKB data (n = 804)^51^, by analysing the spinal cord morphology at the individual vertebral level across C1 to C3 of over 80,000 participants. Established spinal cord processing methods typically require manual intervention to generate vertebral labelling, a bottleneck in processing large-scale imaging data. We were able to overcome this challenge by using an AI-enabled automated imaging analysis pipeline, trained on high-quality spinal cord labels containing severe spinal cord pathological cases, enhancing robustness to anatomical variations and artefacts. The pipeline improved C1 labelling accuracy, reflecting known anatomical patterns and enabling detection of subtle morphological trends such as decreasing AP and increasing RL diameters from C1 to C3^52^. Neck angle correction was implemented to account for individual curvature and improve metric estimation accuracy. Concordance across C1–C3 levels, reproducibility between longitudinal scans, and correlations with structurally connected brain regions provide substantial evidence of this automated approach’s success.

We established that cervical spinal cord morphology exhibits moderate to high SNP-based heritability (h^2^ = 0.16–0.42). These estimates are comparable to previously reported heritability for subcortical and brainstem volumes^16,17,39^. Interestingly, we found that eccentricity, a metric reflecting circular or flattened shape, emerged as the most genetically robust metric, yielding the highest heritability and the greatest number of independent GWAS signals. This supports clinical observations of flatter spinal cords in ataxia patients and suggests eccentricity is largely developmentally determined and stable over time^12^. The longitudinal stability of the eccentricity measure suggests it captures a highly conserved aspect of spinal cord morphology, possibly reflecting early developmental structural variations that are less susceptible to environmental influences. In contrast, size-related metrics (CSA and diameters) show age-related changes and may be more sensitive to pathological processes and environmental factors.

Our large-scale genetic investigation identified 179 genetic variants associated with spinal cord shape metrics, many previously associated with neurological, cardiovascular, and anthropometric phenotypes. One highly associated variant, rs1126642, leads to a missense mutation in *GFAP*, an intermediate filament cytoskeletal gene highly expressed in glial cells, particularly astrocytes. *GFAP* is central to both brain and spinal cord development and pathology, but is also expressed in non-neural tissue^53^, highlighting a broad biological importance for this signal. Sex-specific analyses revealed both overlapping and distinct genetic influences on spinal cord shape in males and females. While most loci were detected in both combined and sex-stratified GWAS, three SNPs showed significant SNP-by-sex interaction effects, including one in the androgen receptor gene, suggesting potential sex-dependent genetic regulation of spinal cord morphology.

Our gene-based analysis identified 96 high-confidence genes likely contributing to cervical spinal cord morphology variation, with several prioritised based on colocalization and heterogeneity thresholds. Among these, *SGTB*, *TGFA*, and *ROR2* emerged as top ranked and biologically plausible gene candidates. For example, *SGTB*, encoding a small tetratricopeptide repeat co‑chaperone, showed strong evidence of colocalization in brain tissues with CSA and diameters. *SGTB* has a key role in shaping neurite morphology and promoting neuron survival^45^ and has been implicated in GWAS of neuroimaging phenotypes^14,39^ and white matter integrity^54^. Meanwhile, *TGFA* promotes proliferation and survival in epithelial and neural tissues during development^46,55^, and is expressed in the developing spinal cord^56^. *ROR2* is essential for axial skeletal development and vertebral patterning, with mutations causing autosomal recessive Robinow syndrome^47^ characterised by vertebral defects. At the gene set level, we found strong enrichment of heritability in pathways related to the development of kidney and other tissues (e.g., Metanephric glomerulus vasculature development with C1 eccentricity). Enrichment of kidney-related pathways may reflect shared developmental processes, as kidney morphogenesis genes are also involved in broader processes of embryonic patterning and tissue differentiation^57^.

We observed moderate negative phenotypic correlations between cervical spinal cord size and several neurological conditions, including MS and neuropathies. These findings were consistent with clinical evidence that spinal cord atrophy has prognostic value for MS progression and treatment response^58,59^, and may help distinguish MS subtypes^60^ supporting the prospect for further studies. We also observed negative correlations between cervical spinal cord size and mono- and polyneuropathies, consistent with spinal cord atrophy reported in both early and severe peripheral neuropathy^61,62^. Additionally, phenotypical correlations with viral infections, many of which are neurotropic^63,64^, musculoskeletal disorders such as osteoporosis and gout arthropathies, and essential hypertension were identified. Using spinal cord PRS in the non-imaging cohort, we validated 11 of 47 tested disease associations identified in the imaging cohort, indicating genetically predicted spinal cord measures can reproduce a subset of phenotypic associations. This aligns with prior findings that PRS only partially reconstruct imaging-derived phenotypes^65^. However, consistent patterns for metabolic and cardiovascular conditions suggest potential utility of PRS as proxies for spinal cord measures in larger cohorts with genetic data. We also noted reporting bias in viral infectious diseases, where a significant number of self-reported conditions, such as chickenpox, rubella, and measles, were added during the subsequent imaging visits, but not for participants who only attended the baseline visit. Building on these analyses, we further evaluated the genetic correlations between spinal cord metrics and a range of disease traits. While we observed evidence of genetic overlap across several conditions, MR analyses did not support robust causal effects.

One limitation of this study is that the discovery GWAS sample consists mainly of European individuals aged 45 years and older. Younger age cohorts may be needed to fully understand the dynamic range of spinal cord signals. Another limitation is that our findings in European populations may not generalise to individuals of other ancestries. We examined whether SNPs identified in the European-ancestry GWAS of spinal cord shape metrics were also associated with the same traits in non-European UKB individuals. However, these analyses were limited in scale and substantially underpowered, with all non-European ancestry groups comprising fewer than 900 individuals (maximum n = 893, in the C/SA group at the C1 level). Given this limited sample size, the absence of statistically significant associations in this subgroup should not be interpreted as evidence against an effect but rather reflects limited power. Larger, ancestrally diverse cohorts will be needed for further study. While we observed a decline in both the number of GWAS associations (partially due to decreased sample size) and decreasing heritability from C1 to C3, these trends can be influenced by the worse image quality of the C3 level. Fully disentangling biological effects from data quality factors is challenging with the UKB data, interpretations of these inter-level trends should be approached with caution.

In summary, this study presents artificial intelligence-enabled large-scale GWAS to uncover novel genetic loci associated with spinal cord shape metrics, providing insights into the genetic architecture of spinal cord. Our findings, combining imaging and genetic evidence, highlight the importance of further investigating the spinal cord’s involvement in human health.

## Methods

### UK biobank

The UKB is a large-scale biomedical database containing in-depth genetic, imaging, and health information from over 500,000 participants. Access to all UKB data was granted on June 18th, 2019, application #36610. This study was conducted in accordance with the Declaration of Helsinki and complied with all relevant national and institutional regulations governing the use of human research participants. UKB has ethics approval from the North West Multi–centre Research Ethics Committee. This study was approved by the Walter and Eliza Hall Institute of Medical Research (WEHI), Human Research Ethics Committee (HREC reference 17/09LR).

### MRI acquisition and image sample

MRI image acquisition was done following the UKB imaging protocol (https://biobank.ctsu.ox.ac.uk/crystal/crystal/docs/brain_mri.pdf). In brief, brain imaging was acquired across four identical imaging centres in the UK. T1-weighted (T1-w) structural scans were acquired using a 3D MPRAGE protocol (voxel size = 1 × 1 × 1 mm^3^, matrix size = 208 × 256 × 256, inversion time (TI)/TR = 880/2000 ms, in-plane acceleration = 2, total scan time = 4 min 54 s).

We used the late 2023 release of brain MRI data (v15.1) for the discovery cohort. Additional data released in 2025 (v20.5) were included for replication. The UKB standard QC^66^ pipeline was applied to exclude any images of poor quality and severe artefacts (labelled as unusable). Although T1-w images were processed using the UKB image processing pipeline, our study used the unprocessed data with the largest field of view to preserve the spinal cord region. After the initial standard QC, a total of 46,366 (discovery), 35,530 (replication), and 4,578 repeat scans underwent further spinal cord processing.

### Spinal cord processing pipeline

We implemented an optimised deep learning-based spinal cord processing pipeline for upper spinal cord labelling (https://github.com/art2mri/Enigma-SC), designed for large-scale and reliable spinal cord shape analysis (Fig. 1a). The pipeline begins with spinal cord segmentation using the *sct_deepseg_sc* command from the Spinal Cord Toolbox (SCT)^25^, followed by vertebral labelling of C1–C3 using a pretrained nnu-Net deep learning model specifically tuned for the upper spinal cord^35^. From the resulting segmentation masks and vertebral labels, spinal cord shape metrics are extracted. This pipeline addresses several key challenges: (1) it enables fully automatic and accurate identification of C1–C3 without the need for manual correction; (2) robustness to pathological variations, neck angle differences, and partial coverage; and (3) it significantly improves the accuracy of C1 labelling by leveraging a training set curated with high-quality C1 annotations. In addition, CSA measurements were corrected for neck curvature using AP and RL angles derived from the shape analysis.

### Spinal cord phenotype QC

To address the problem of image quality variability within the same scan and to maximise sample size, we implemented a multi-step QC procedure (detailed in Supplementary Section 14). First, T1-weighted images with poor overall quality were excluded based on the standard UKB QC flag. Next, given spinal cord phenotypes had not been extracted in large-scale, we used a conservative manual QC approach (Supplementary section 14.2). Vertebral-level-specific QC was applied by manually inspecting all vertebral labels from C1 to C3 to exclude mislabelled or poor-quality regions. Finally, we removed a small number of outlier points with large discrepancies between metrics derived from the spinal cord segmentation mask vs. the vertebral labels, typically caused by segmentation inaccuracies (Supplementary section 14.3). This process enabled rigorous exclusion of inaccurate data while preserving as much high-quality information as possible to maintain statistical power for downstream GWAS.

### Phenotyping robustness and reproducibility evaluation

For the metric extraction step, we further considered an alternative processing method to ensure the derived spinal cord phenotypes are not prone to processing method and coordinate space choices. Atlas based analysis was performed by adding an additional registration step after vertebral labelling. In brief, the spinal cord of the PAM50 atlas^67^ was straightened and aligned to the native space. Shape metrics were extracted from the registered PAM50 vertebral level images. Correlations between the atlas-based method vs. the native-space spinal cord processing pipeline used in the current study are calculated (Supplementary Section 2.2).

Reproducibility evaluation was performed by calculating the correlations between repeat scans. A small subset of participants underwent repeat scan (1–7 years after the first scan). Follow-up data were available for *n* = 4374, 4270, and 3290 participants at C1, C2, and C3 respectively. Spearman’s correlations (robust to outliers) between the first and second scans were calculated to assess their repeatability, stratified by scan intervals (Supplementary Section 2.1).

### Spinal cord IDP and covariate selection

SCT outputs nine shape metrics per vertebral level: CSA, eccentricity, AP diameter, RL diameter, AP angle, RL angle, orientation, solidity, and total length. However, not all of these reflect biologically meaningful variation suitable for GWAS. We selected relevant phenotypes based on two key criteria: (1) stability across processing steps: metrics such as orientation, angles, sum length can be influenced by image straightening or rotation, making them modifiable during processing; and (2) repeatability across imaging sessions: measures that reflect transient conditions, such as head positioning during a specific scan, were excluded. Based on our robustness and repeatability analyses (Supplementary Section 2), we identified four phenotypes, CSA, eccentricity, AP diameter, and RL diameter, as consistently measurable and biologically interpretable, and thus selected them for GWAS to investigate their genetic architecture.

To confirm that these phenotypes captured both shared and distinct information across C1–C3, we assessed their interrelationships using linear regression models, adjusting for sex, age, height, ICV, imaging site, and genetic principal components 1–10. Principal component analysis was also conducted to explore the underlying structure of these shape metrics and to identify statistical outliers for exclusion (Supplementary Section 4).

Previous spinal cord studies suggested that age, sex, brain size and ICV were relevant covariates that were recommended to be included for normalisation^30^. We performed additional stepwise regression analysis including additional potential confounding factors including scanning site and T1 signal-noise-ratio (Data-Field 25734) to verify relevant covariates to be included in the genome wide association analysis (Supplementary Section 4). We also performed genome-wide association analysis without correcting for the effect of body height as a sensitivity analysis.

### Genotyping, quality control and imputation

Genotyping, QC and imputation for the UKB cohort were performed by UKB and have been described previously^68^. We used the imputed genetic dataset based on the TOPMed r2 reference panel (Data-Field: 21007), which includes 97,256 deeply sequenced human genomes and ~308 million genetic variants from the autosomal and X chromosomes^69^. All QC and analyses of genetic data were performed on the UKB Research Analysis Platform (RAP).

Filtering of the genetic data was undertaken using PLINK2 (v2.0.0-a.6.9)^70^. Variants were excluded if they had a MAF < 0.001 or deviated from Hardy–Weinberg equilibrium (*P* < 1 × 10⁻¹⁵). Imputation quality metrics were extracted from the VCF helper files, and variants identified for exclusion if they had low imputation quality, defined as R^2^ < 0.6, or empirical R^2^ < 0.6 (directly genotyped variants only). We conducted the analysis on the autosomes (chromosomes 1 to 22) and the X chromosome.

Sample QC was undertaken using metrics made available by UKB (Data Fields 22001; 22019; 22027; 22021). We excluded individuals who had discordant self-reported and genetically inferred sex, or sex chromosome aneuploidy; or were flagged by UKB prior to imputation for high heterozygosity, high missingness ( > 5%); or having an excessive number of relatives ( ≥ 10) in the cohort. Individuals who had withdrawn consent were also excluded. To reduce potential confounding, we further excluded individuals with conditions affecting the cervical spine, as identified by ICD-10 diagnostic codes (Data-Field 41270) and operative procedure (OPCS-4, Data-Field 41272), with detailed codes provided in Supplementary Data 1.

Genetic ancestry principal components (PCs) were obtained from the Pan-UK Biobank project (https://pan.ukbb.broadinstitute.org)^37^. Individuals of European, C/SA, AFR, and EAS ancestries were selected for inclusion in the analysis. Individuals from other ancestry groups were excluded due to limited small sample sizes ( < 200 individuals per group).

### Genetic association analyses for spinal cord shape metrics

GWAS were conducted for the 12 spinal cord shape metrics using REGENIE (v2.1.0)^38^. For the discovery GWAS, analyses were restricted to individuals of European genetic ancestries, who passed all genetic and imaging QC. All GWAS were adjusted for age, sex, age², genotyping batch, imaging site, ICV, height, inverted signal-to-noise ratio in T1-weighted images, and the first ten genetic PCs to account for population stratification. Rank inverse normal transformation was applied to all phenotypes, and an additive genetic model was assumed. The REGENIE method involves two steps. In Step 1, a whole-genome regression model was fitted, using only directly genotyped variants with MAF > 0.01% to generate polygenic predictions for each phenotype, which were further decomposed into 23 chromosome-specific predictions using a leave-one-chromosome-out (LOCO) scheme. In Step 2, imputed UKB genotype data were tested for association with the phenotype conditional upon the predictions from Step 1. For analyses of the X chromosome, hemizygous males were coded as homozygous. Quantile–quantile (Q–Q) plots, genomic inflation factor (λ), and LDSC^44^ regression intercept were used to evaluate potential systematic bias in the association results.

### Identification of significant independent SNPs

Significant independent SNPs were identified using the stepwise model selection procedure implemented in GCTA-COJO (v1.94.4)^71^, with LD estimated from the same European-ancestry discovery cohort. A 10 megabase (Mb) window was used to define regions of complete LD, and genome-wide significance was set at the conventional threshold of *P*  <  5 × 10⁻⁸.

To confirm all variants identified by GCTA-COJO were truly independent signals, we further performed conditional analyses using REGENIE^38^, retaining all parameters and Step 1 models from the unconditioned analysis. In Step 2, we conditioned on the variant with the lowest *P* value within a 1 Mb window to assess whether the association for each independent signal remained genome-wide significant (*P*  <  5 × 10⁻⁸).

### Reporting of significant SNPs and loci

To account for multiple testing across the 12 spinal cord shape metrics, a Bonferroni-corrected threshold of *P*  <  4.17 × 10⁻⁹ (5 × 10⁻⁸/12) was applied for final reporting and downstream follow-up of SNPs. All significant independent SNPs were annotated using Functional Annotation of Variants - Online Resource (FAVOR)^72^, with the variant function, and (nearest) gene, based on GENCODE annotations^73^.

To examine the overlap of findings across the 12 traits, we grouped independent SNPs into loci based on their proximity and linkage disequilibrium. Specifically, SNPs were considered part of the same locus if they were within 250 kilobase (kb) of each other and had a pairwise linkage disequilibrium of r² >0.1.

To determine whether our independent SNPs had been previously reported, we downloaded the GWAS Catalog association summary statistics (accessed 22 July 2025) and cross-referenced them with the GWAS Catalog. To improve the integration of GWAS Catalog phenotype categories, we used the EFO Ontology Trait Mappings (https://www.ebi.ac.uk/gwas/api/search/downloads/trait_mappings) to align the traits with their corresponding ontology terms and parent terms.

### SNP-based heritability

We used LDSC (v.1.0.1)^44^ to estimate SNP-based heritability (*h*^*2*^) for each spinal cord shape metric using GWAS summary data. Precomputed LD scores from the 1000 Genomes Project European reference population^74^ were used as the reference panel, and analyses were restricted to HapMap3 SNPs^75^.

### Sex-specific GWASs and sex interaction analyses

Sex was determined using genetic data. We obtained sex information from UK Biobank Data-Field 22001. Sex was included as a covariate in all analyses. For the primary analysis, x19,552 (52.7%) individuals were female, 17,516 (47.3%) were male. Detailed demographic characteristics are reported in Supplementary Data 35. To investigate sex differences in the genetic architecture of spinal cord shape metrics, we conducted sex-specific GWAS using REGENIE, and identified independent SNPs using GCTA-COJO as in the sex-combined discovery analyses. We defined sex-specific signals as independent SNPs meeting *P* < 4.17 × 10⁻⁹ in one of the sex-specific analyses, but not in the corresponding sex-combined analysis. To confirm these sex-specific signals, we verified that the top SNP was not in LD (r² > 0.1) with any independent SNP identified through the sex-combined GWAS.

For all independent, significant SNPs identified through either the sex-combined, or sex-specific analyses, we sought evidence of SNP-by-sex interaction, by repeating REGENIE’s step 2, with the addition of the interaction flag. REGENIE’s geneby-environment interaction model uses a Wald test with sandwich estimators of variance for variants whose minor allele count (MAC) > 1,000, with a heteroskedastic linear model used for variants of lower frequencies^76^. We defined evidence of a SNP-by-sex interaction based on the interaction term *p* value meeting a Benjamini-Hochberg false discovery rate (FDR) adjusted threshold of 0.05.

### Associations in non-European ancestries

We assessed whether the independent significant SNPs identified in the European-ancestry GWAS of spinal cord shape metrics were also associated with the same traits in individuals of C/SA, AFR, and EAS ancestry from the UKB. Association analyses were conducted separately for each ancestry group using REGENIE, following the same procedures as in the discovery analysis. To focus on common variants, we excluded SNPs with a MAF < 0.05 within each respective population cohort.

Replication was defined as achieving significance at a Bonferroni-corrected threshold, adjusted for the number of loci tested (*P* < 0.05/179). We then compared effect sizes between the European and non-European groups by calculating Spearman’s Rank Correlation coefficients and the proportion of SNPs with concordant effect directions. A two-sided binomial test assessed whether observed concordance differed from that expected under random directionality.

### Gene-based tests

GWAS summary statistics for each of the 12 spinal cord traits were lifted over from genome build hg38 to hg19 using the UCSC LiftOver tool with the appropriate chain files.

### Magma

MAGMA (Multi-marker Analysis of GenoMic Annotation) was used to identify risk genes near genome-wide significant loci for the 12 spinal cord shape phenotypes. This approach assigns variants to genes based on their physical proximity to a defined set of genic coordinates. We mapped variants to protein coding genes obtained from the MAGMA website (https://cncr.nl/research/magma/). Genic coordinates extended 10 kb upstream and downstream to capture regulatory variation. After mapping variants to genes, gene-based *P* values were calculated using the SNP-wise mean model. We used samples from the 1000 Genomes Project (Phase 3) as the LD reference panel.

### mBAT-combo

We performed a gene-based analysis utilising the multivariate Set-Based Association Test (mBAT-combo) within GCTA version 1.94.1. This method enhances the detection power for multi-SNP associations, especially in the presence of masking effects, by combining mBAT and fastBAT test statistics through a Cauchy combination approach. This allows for the integration of different test statistics without prior knowledge of their correlation structure, thereby maximising overall power regardless of masking effects at specific loci. We used the European subsample (*n* = 503) from Phase 3 of the 1000 Genomes Project as the LD reference panel, applying the fastBAT default LD cut-off of 0.9. A gene list of 19,899 protein-coding genes was utilised to map base pair positions according to the genome build hg19.

### Transcriptome-wide association study

We performed a transcriptome-wide association study (TWAS) using the FUSION method, which integrates GWAS summary statistics with gene expression prediction models to identify associations between gene expression and the 12 spinal cord phenotypes. We utilised brain gene expression weights from the PsychENCODE project, which includes expression data from the dorsolateral prefrontal cortex. We incorporated LD information from the 1000 Genomes Project Phase 3 to estimate the genetic component of gene expression. This genetic component was then tested for association with each spinal cord phenotype using GWAS summary data. To correct for multiple testing, we applied the Bonferroni correction (i.e., 0.05/number of tests performed). We used the COLOC R package^77,78^, implemented using FUSION, to identify TWAS associations (i.e., trait-gene expression associations) that are likely to share a causal SNP. Colocalisation calculates posterior probabilities (PP) to assess whether individual lead SNPs within a significant TWAS locus are: (i) independent signals (PP3) (e.g., two causal SNPs in LD, one affecting transcription and the other affecting spinal cord morphology), or (ii) shared signals (PP4) (e.g., a single causal SNP affecting both transcription and spinal cord morphology).

### Summary-based Mendelian randomisation

We used Summary data-based Mendelian Randomisation (SMR) to integrate GWAS and expression quantitative trait loci (eQTL) data from human brain and blood samples. This method leverages summary-level data in a Mendelian Randomisation statistical framework to test for pleiotropic associations between gene expression and GWAS data. We used whole blood expression data from the eQTLGen consortium and brain expression data from the MetaBrain resource^79^. To assess the presence of linkage or pleiotropy, we performed the Heterogeneity in Dependent Instruments (HEIDI) test alongside SMR, evaluating effect size heterogeneity between the GWAS and eQTL summary statistics. The HEIDI test is designed to distinguish between pleiotropy and linkage by assessing heterogeneity in the association signals. A HEIDI *P* value threshold of 0.05 was used to determine the likelihood of linkage influencing the observed association.

### Aggregation of gene-based tests

Results from the six gene-based tests were aligned to a common gene reference (GENCODE v47) before aggregation. Multiple-testing correction was applied within each gene-based test and spinal cord phenotype separately. For the multi-tissue panels (FUSION TWAS across 13 GTEx v8 brain tissues, and the SMR panels) multiple testing correction was done on the number of gene-by-tissue tests, and the most significant tissue per gene was selected only after correction, so tissue selection does not inflate significance. Both Benjamini-Hochberg FDR and Bonferroni-adjusted thresholds of 0.05 were computed. Genes were then aggregated across the 12 phenotypes: a test was counted as implicating a gene if that gene reached the threshold for any of the four morphological measures at any cervical level. Similarly, per-measure counts implicated a gene if it was significant at any of C1-C3. Convergence was then quantified as the number of tests implicating each gene at Bonferroni significance. Genes passing the significance thresholds were further prioritised using the TWAS colocalisation filter (PP.H4 > 0.8) and the SMR HEIDI test (*P* > 0.05).

### Gene set enrichment analysis with GSA-MiXeR

Gene set analysis was conducted using GSA-MiXeR to quantify the enrichment of genetic signals across 12 spinal cord phenotypes within gene ontology (GO) categories. We used GSA-MiXeR rather than gene-set analysis of the convergent gene list to avoid biases arising from gene selection by the individual gene-based tests (such as gene length, SNP density and cis-eQTL discoverability, which are properties also correlated with pathway annotation) and because the tests share no common background appropriate for over-representation testing. GWAS summary statistics were analysed with GSA-MiXeR using default parameters as described by Frei et al. ^80^. The method employs a competitive approach to gene set analysis, estimating fold enrichment of partitioned heritability through maximum likelihood optimisation, accounting for LD, MAF, and polygenicity. We used the FDR (*P* < 0.05) to control for multiple comparisons in MAGMA analyses. Gene ontology sets were prioritised based on their fold enrichment, allowing assessment of biologically relevant gene sets associated with each spinal cord phenotype.

### Associations between spinal cord and other brain phenotypes

A total of 41 volumes were selected including four brainstem subregions: pons, medulla, midbrain, superior cerebellar peduncle (SCP) (Category 191); 9 subcortical regions: caudate, cerebellum (white matter [WM] + grey matter [GW]), hippocampus, pallidum, putamen, thalamus, ventral diencephalon, amygdala, accumbens (Category 191); 28 cortical BA volumes in the left and right hemisphere (Category 195). Phenotypic association with ICV was also calculated as a reference. Inverse normalisation transformation was applied to spinal cord shape metrics, then linear regression model was used to calculate the associations between spinal cord IDPs and other brain volumes, correcting for the effects of sex, age, and genetic principal components 1–10.

### Phenotypic Associations with health-related outcomes

ICD-10 outcomes were extracted from the UKB (Category 1712), incorporating data from multiple sources: primary care records, hospital inpatient admissions, death registries, and self-reported medical conditions. These ICD-10 codes were then mapped to the corresponding Phecode Map X. Because a single ICD-10 code can map to multiple PhecodeX entries, we selected the most appropriate PhecodeX based on the following criteria: (1) preserving the ICD-10 category, and (2) best aligning with the specificity of the original ICD-10 code (Supplementary Data 41). This mapping process yielded 778 unique PhecodeX disease codes from 1,165 ICD-10 codes for subsequent association analysis.

The extracted diagnostic data were converted into binary format, where 1 indicated the presence of a condition (cases) and 0 indicated absence (controls). To ensure adequate statistical power, we restricted analyses to diseases with at least 100 cases in the European samples, with spinal cord phenotype data available. With 100 cases, the power to detect an association at α = 1 × 10⁻⁵ is 61%, 93%, and 99% for odds ratios of 1.6, 1.8, and 2.0, respectively, per one standard deviation increase in the predictor.

Logistic regression models were used to assess associations between each spinal cord phenotype and PhecodeX conditions, adjusting for sex, age, height, intracranial volume (ICV), imaging site, and the first 10 genetic principal components. We also incorporated sex-specific information from PhecodeX, such that female-only and male-only diseases were analysed in the corresponding sex, while conditions applicable to both sexes were analysed jointly. A total of 3,996 tests were performed across 12 spinal cord phenotypes. An FDR corrected threshold of *P* < 0.05 was considered significant. Similar analyses were conducted in the non-European cohort. Due to limited sample sizes, we required a minimum of 20 cases per disease^81,82^, resulting in a total of 130 diseases tested for non-European samples.

### Polygenic risk scores in the non-imaging cohort

Using GWAS summary statistics from spinal cord imaging phenotypes, we constructed polygenic risk scores (PRS) and applied them to UK Biobank participants who did not undergo brain MRI (N = 353,322 European ancestry). Genome-wide PRS were constructed using PRS-CS^83^, a Bayesian regression framework that applies continuous shrinkage priors to adaptively estimate SNP effect sizes while accounting for linkage disequilibrium (LD). The GWAS summary statistics were quality controlled as follows: (i) variants with imputation INFO ≤ 0.8 or minor allele frequency ≤1% were excluded; (ii) SNPs with duplicated rsIDs and indels were removed; (iii) variants on the X chromosome were excluded; and (iv) summary statistics were lifted over to the hg19 reference build. The UKB European LD reference panel (ldblk_ukbb_eur; https://github.com/getian107/PRScs) was used. We applied the PRS-CS auto model, which infers the global shrinkage parameter directly from the summary statistics, with the following default settings: γ–γ prior parameters a  =  1 and b  =  0.5, 1000 total Markov chain Monte Carlo (MCMC) iterations, 500 burn-in iterations, and a thinning factor of 5.

### Validation of phenotypic disease associations using spinal cord PRS

These PRS allowed us to evaluate whether genetic proxies of spinal cord traits could reproduce disease associations observed in the phenotypic associations. Previous work has demonstrated that PRS generated from imaging-derived phenotypes can uncover links with diverse complex traits and diseases in non-imaging samples^84–86^. In a similar manner, we used this analysis to assess whether the disease associations observed with imaging-derived traits can be reproduced using PRS.

We focused on diseases that showed significant associations with spinal cord phenotypes in the imaging cohort (defined as at least two phenotypes with FDR-corrected *P* < 0.05). This set of diseases was then tested for association with spinal cord PRS. We first removed related individuals by identifying those within third-degree relatedness (kinship coefficient >0.0625). To maximise the inclusion of disease cases, related individuals were selected by prioritising the retention of rarer disease cases among this set of diseases. After this step, *n* = 317,730 participants with spinal cord PRS remained for analysis. Association testing was performed using logistic regression, with each disease as the outcome and the relevant PRS as the predictor, adjusted for sex, age at baseline, and the first 10 genetic principal components.

### Genetic correlations with other brain phenotypes and diseases

We used cross-trait LDSC to estimate genetic correlations within spinal cord shape metrics and between these metrics and a range of brain IDPs, including measures from the cortical, subcortical, and brainstem regions, as well as various diseases. LDSC accounts for sample overlap and population stratification, providing robust estimates of shared genetic architecture. Phenotypes were selected based on prior evidence of relevance to spinal cord structure, observed phenotypic associations, and data availability. GWAS summary statistics used in these analyses are listed in Supplementary Data 42. Multiple testing correction for all cross-trait analyses was performed using the Benjamini–Hochberg procedure to control the FDR (*P* < 0.05).

### Mendelian randomisation

For the traits which showed significant genetic correlations with spinal cord metrics, and had a sufficient number (≥3) of associated genetic variants to act as instruments, we conducted Mendelian randomisation (MR) to explore potential causal effects on spinal cord shape metrics. MR is a form of instrumental variable analysis whereby genetic variants (“instruments”) are used to examine the causal relationship between one trait (“exposure”) and another (“outcome”). There are three core assumptions central to MR: 1) the genetic instruments must be significantly associated with exposure of interest; 2) no confounding should exist between the genetic instruments and the outcome; and 3) the genetic instruments must not be associated with the outcome, via any pathway, other than through the exposure (i.e., no horizontal pleiotropy).

We utilised a two-sample MR approach, which is intended to leverage GWAS summary statistics from non-overlapping samples for exposure and outcome. For all exposure traits (i.e., the non-spinal cord phenotypes), we used the same published GWAS summary statistics as in the genetic correlation analyses (Supplementary Data 42). We note the GWAS for these traits were large meta-analyses which, with the exception of ADHD, included UKB samples. This has the potential to lead to biased estimates from two-stage MR methods, depending on the level of overlap^87,88^.

Our primary MR analyses were performed using the inverse-variance weighted (IVW) method^48^, as implemented in the TwoSampleMR R package^89,90^. Genetic instruments for each exposure were obtained using LD-clumping, using a 10,000 kb window, a clumping *P* value threshold of 5 × 10⁻⁸, an r² cut-off of 0.001, and LD estimated from 1000 Genomes European samples. When extracting instruments from the outcome (spinal cord phenotypes), GWAS summary statistics, proxies were not considered in place of unavailable SNPs. Results passing the Benjamini–Hochberg procedure to control the FDR at *P*  <  0.05 were considered significant.

### Mendelian randomisation sensitivity analyses

In addition to the standard IVW approach, we repeated the MR analyses using a series of other methods and approaches, allowing us to evaluate the validity of the genetic instruments and assess potential violations of the core MR assumptions.

First, we repeated the MR analyses using the MR-Egger^49^, weighted median^91^, and weighted mode^92^ methods, all implemented in the TwoSampleMR R package^89,90^. These methods provide consistent estimates, even where not all of the genetic instruments are valid. We examined the MR-Egger intercept to test for directional pleiotropy; a non-zero intercept suggests the presence of directional horizontal pleiotropy, which violates assumption 3, and could bias the MR estimate. We also assessed heterogeneity of instrument effects using Cochran’s Q statistic; significant heterogeneity may also be indicative of horizontal pleiotropic effects.

As an alternative approach, we implemented the Causal Analysis Using Summary Effect estimates (CAUSE)^50^ model (v1.2.0, https://github.com/jean997/cause). CAUSE accounts for shared genetic effects between traits and provides estimates robust to both correlated and uncorrelated horizontal pleiotropy. CAUSE can also account for shared genetic effects that might arise from sample overlap. Briefly, in step 1 we estimated the nuisance parameters, using the default settings, and 1 million randomly selected genome-wide variants. In step 2 (estimation of causal effects), we used filtered and pruned variants (*r²*  <  0.001 within 10 Mb windows) based on two *P* value thresholds: *P*  <  0.001 and *P*  <  5 × 10⁻⁸.

Finally, to investigate potential bidirectional relationships, we conducted reverse MR analyses. In these analyses, we used genetic instruments for spinal cord phenotypes as the exposure, with our original exposure traits as the outcome. Spinal cord genetic instruments were selected as those identified in the COJO analysis at a significance threshold of *P*  <  5 × 10⁻⁸/12. Reverse MR was carried out for inverse variance weighting, MR-Egger, weighted median, and weighted mode methods.

### Reporting summary

Further information on research design is available in the Nature Portfolio Reporting Summary linked to this article.

## Supplementary information

Supplementary Information

Description of Additional Supplementary File

Supplementary Data 1

Supplementary Data 2

Supplementary Data 3

Supplementary Data 4

Supplementary Data 5

Supplementary Data 6

Supplementary Data 7

Supplementary Data 8

Supplementary Data 9

Supplementary Data 10

Supplementary Data 11

Supplementary Data 12

Supplementary Data 13

Supplementary Data 14

Supplementary Data 15

Supplementary Data 16

Supplementary Data 17

Supplementary Data 18

Supplementary Data 19

Supplementary Data 20

Supplementary Data 21

Supplementary Data 22

Supplementary Data 23

Supplementary Data 24

Supplementary Data 25

Supplementary Data 26

Supplementary Data 27

Supplementary Data 28

Supplementary Data 29

Supplementary Data 30

Supplementary Data 31

Supplementary Data 32

Supplementary Data 33

Supplementary Data 34

Supplementary Data 35

Supplementary Data 36

Supplementary Data 37

Supplementary Data 38

Supplementary Data 39

Supplementary Data 40

Supplementary Data 41

Supplementary Data 42

Reporting Summary

Transparent Peer Review file

## Source data

Source Data

## Data Availability

The data used in the study are obtained from UKB Resource under Application Number 36610. All genetics, health related information, and imaging data described in this paper are publicly available to registered researchers through the UKB data-access protocol. Access to UK Biobank data is restricted to approved researchers with a valid application; additional information about registration for access to the data are available at: (https://www.ukbiobank.ac.uk/enable-your-research/apply-for-access). The raw spinal cord phenotype data generated in this study will be returned to UK Biobank, and researchers can access them through UK Biobank upon approved application. Supplementary data are provided as Supplementary Data files uploaded with this submission. The summary statistics from the spinal cord morphology discovery GWAS are available in the GWAS Catalog under accession numbers GCST90994711-GCST90994722. Other GWAS summary statistics generated in this study have been made available to download from https://osf.io/9s6gk. Source data are provided with this paper.

## Code availability

The spinal cord image processing pipeline is publicly available at: https://github.com/art2mri/Enigma-SC

No custom code was used in this study. Publicly available software tools were used to perform genetic analyses and are referenced throughout the paper.
