# Supplementary material for: Genetic architecture of MRI-derived cervical spinal cord morphology reveals sensory-motor axis and systemic disease associations
Source: Nat Commun. 2026 Sep 16;17:10904. doi: 10.1038/s41467-026-77419-x (PMC13677749; doi:10.1038/s41467-026-77419-x)
Supplement: Supplementary file 1 [file 41467_2026_77419_MOESM1_ESM.pdf]

Supplementary Materials

Section 1: Descriptive summary of spinal cord phenotypes **1-2**

Supplementary figure 1-2: Phenotype summary *1-2*

Section 2: Phenotyping robustness and reproducibility evaluation **3-7**

Supplementary figure 3-4: Repeatability evaluation *3-4*
Supplementary figure 5-8: Evaluation of method robustness *5-7*

Section 3: Genome-wide association (GWA) results in the European ancestry **8-13**

Supplementary figure 9-12: Manhattan and Q-Q plots *8-11*
Supplementary figure 13: Loci summary for 12 spinal cord metrics *12*
Supplementary figure 14: MAF vs. effect size for significant variants *13*

Section 4: GWAS covariate selection **14**

Supplementary figure 15 Summary of GWAS covariates selection *14*

Section 5: GWA results (Excluded height as a covariate) **15-18**

Supplementary figure 16-19: Manhattan and Q-Q plots (without correcting for height) *15-18*

Section 6: Heritability sensitivity analysis for spinal cord shape metrics **19**

Supplementary figure 20: Heritability estimation of spinal cord shape metrics *19*

Section 7: Sex-specific GWA results **20-25**

Supplementary figure 21-24: Miami plots *20-23*
Supplementary figure 25: Sex-specific SNP heritability correlation *24*
Supplementary figure 2*6*: Significant SNP-by-sex interaction effects *25*

Section 8: Association in the replication GWAS **26-31**

Supplementary figure 27-30: Manhattan and Q-Q plots *26-30*
Supplementary figure 31: Effect size comparison: European discovery vs. European replication *31*

Section 9: GWA results in non-European ancestries **32-34**

Supplementary figure 32-34: Effect size comparison: European vs. non-European cohorts *32-34*

Section 10: Gene-based findings **35**

Supplementary figure *35*: Gene-based findings *35*

Section 11: Correlations between spinal cord phenotypes and brain structures **36**

Supplementary figure 36: Correlations between spinal cord shape metrics and brain volumes *36*

Section 12: Polygenic risk associations with disease outcomes **37-39**

Supplementary figure 37-38: Phenotypic vs. PRS disease associations *37-38*
Supplementary figure 39: Reporting sources for rubella viral infection *39*

Section 13: Mendelian randomization **40**

Supplementary figure 40: Mendelian randomization *40*

Section 14: Spinal cord image quality control (QC) **41-44**

Supplementary figure 40: Image QC overview *41*
Supplementary figure 41-42: Manual QC on vertebral labels *42-43*
Supplementary figure 43: Additional removal of noise between processing steps *44*

References **45**

Section 1: Descriptive summary of spinal cord phenotypes

a

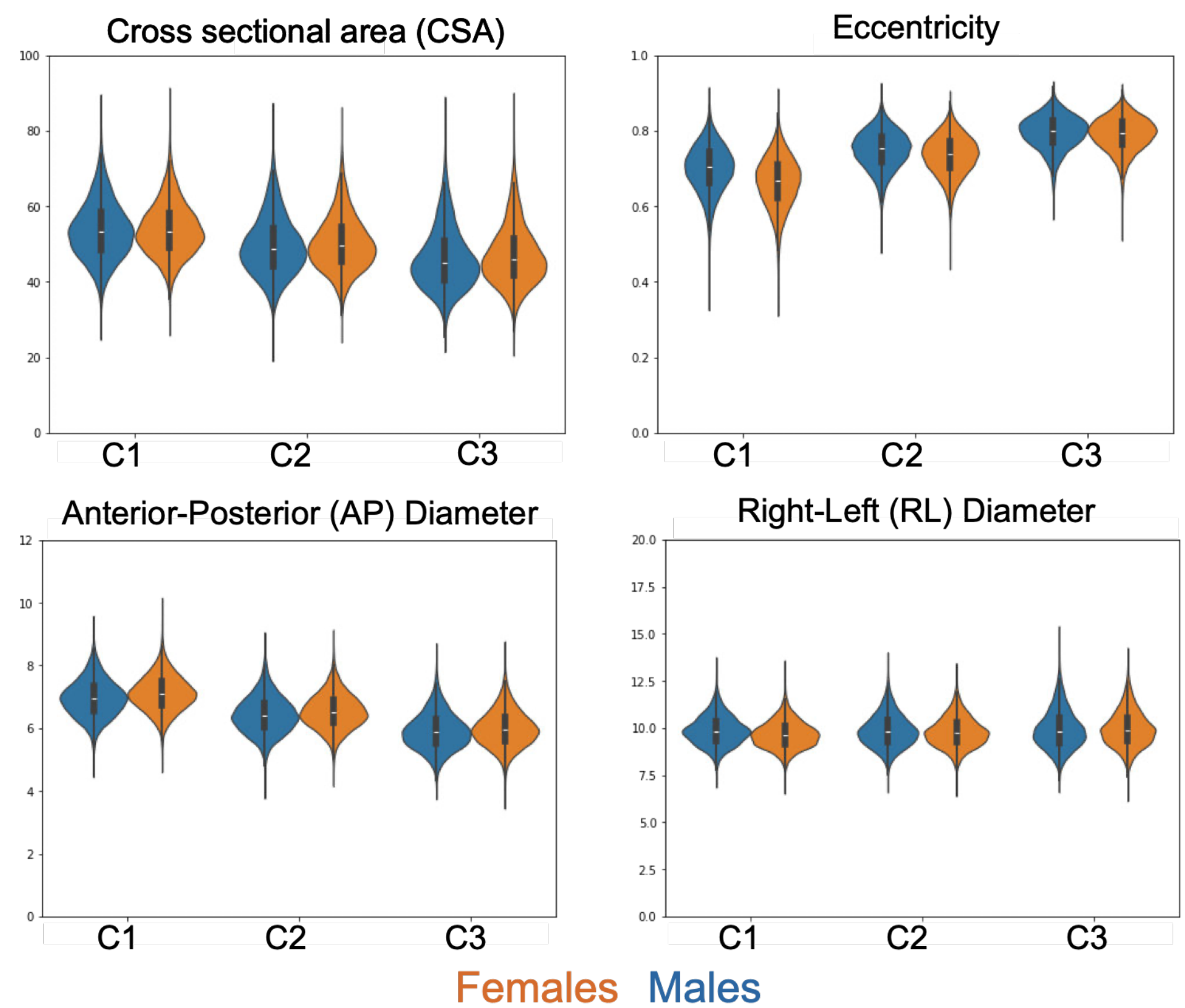

b

	C1	C2	C3
# sample	45684	44737	40024
CSA	53.87 ± 7.37	50.01 ± 7.53	46.65 ± 7.98
diameter AP	7.04 ± 0.59	6.49 ± 0.58	5.96 ± 0.59
diameter RL	9.77 ± 0.76	9.83 ± 0.83	9.96 ± 0.99
eccentricity	0.68 ± 0.07	0.74 ± 0.05	0.79 ± 0.04
sum(length)	11.65 ± 0.97	14.32 ± 1.03	15.41 ± 1.56

Supplementary Figure 1: Distribution of spinal cord phenotypes across cervical vertebral levels. (a) Violin plots of spinal cord phenotypes after imaging QC at each vertebral level, stratified by sexes (orange: female; blue: male). Violin width represents the kernel density estimate of the phenotype distribution. The overlaid box plot shows the median (center line), interquartile range (box bounds, 25th–75th percentile), and whiskers extending to 1.5× the interquartile range beyond the box. Sample sizes (n, independent UK Biobank participants) for each level and sex are provided in panel b. (**b**) The corresponding sample size, mean and standard deviation of each spinal cord phenotype across C1-C3 in units of mm for diameters and mm^2 for cross-sectional area (CSA). Source data are provided as a Source Data file.

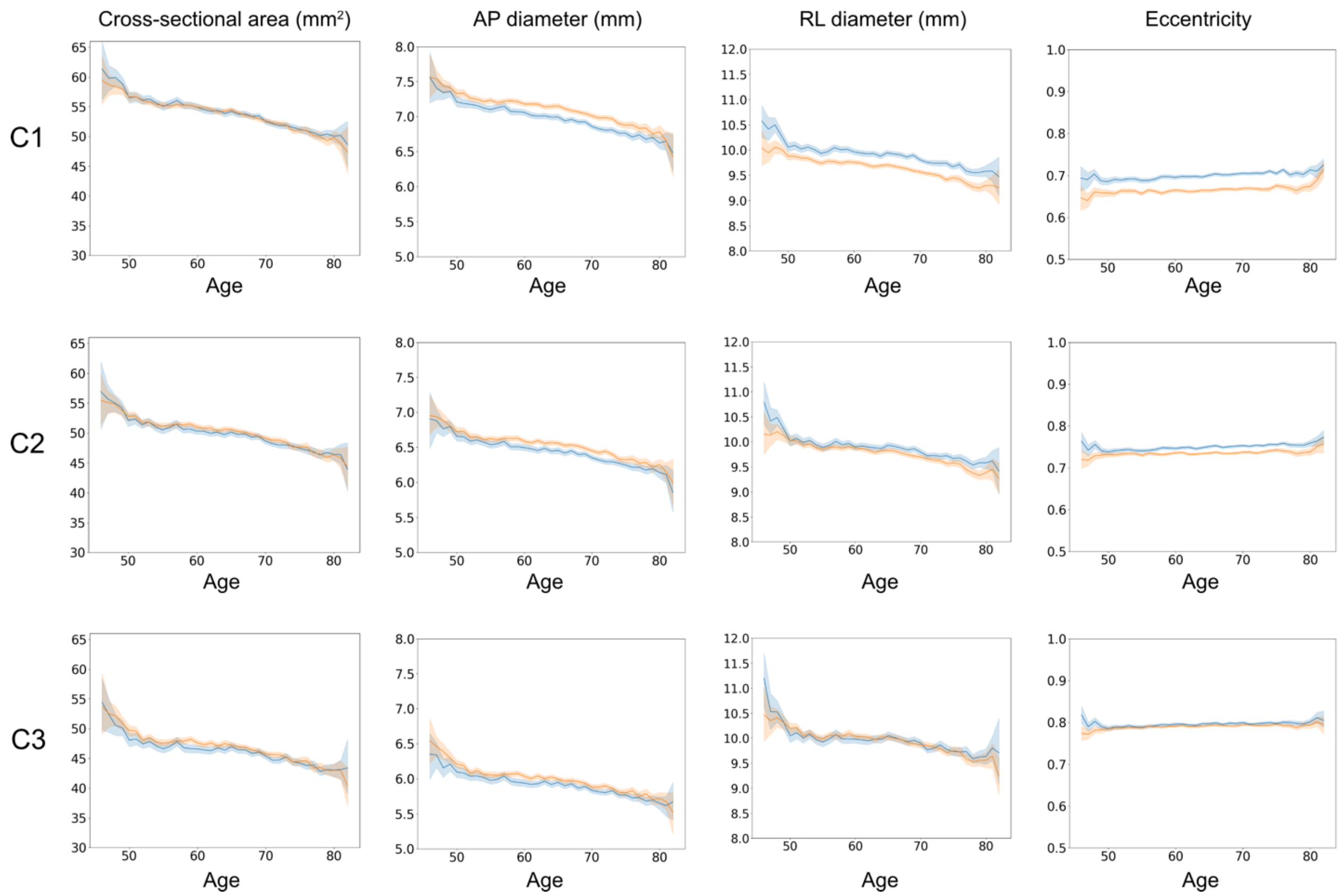

Supplementary Figure 2: Line plot showing the mean spinal cord phenotype across age stratified by sex (orange: female; blue: male). The x-axis represents age (years), and the y-axis indicates the average value of the phenotype (CSA in mm²; AP and RL diameter in mm; eccentricity unitless). Shaded regions denote the 95% confidence interval (CI) of the mean estimate at each age from 45 to 83. CSA, cross-sectional area; AP, anterior-posterior; RL, right –left. Source data are provided as a Source Data file.

Section 2: Phenotyping robustness and reproducibility evaluation

2.1 Repeatability evaluation

To ensure cross-scan consistency, we conducted a repeatability evaluation using data from participants who underwent repeat imaging. Only data from the first scan session was used in the primary GWAS analysis. Following the same QC procedure, we obtained 4374, 4270, and 3290 spinal cord IDPs for C1, C2, and C3 respectively for reproducibility analysis. The average age at the first scan was 61.5 ± 7.5 years, and 64.2 ± 7.3 years at the second scan (Supplementary Figure 3). Image quality was slightly lower in the repeat scans, resulting in a higher failure rate during QC especially at the C3 level. This is potentially due to age-related imaging artifacts in older participants.

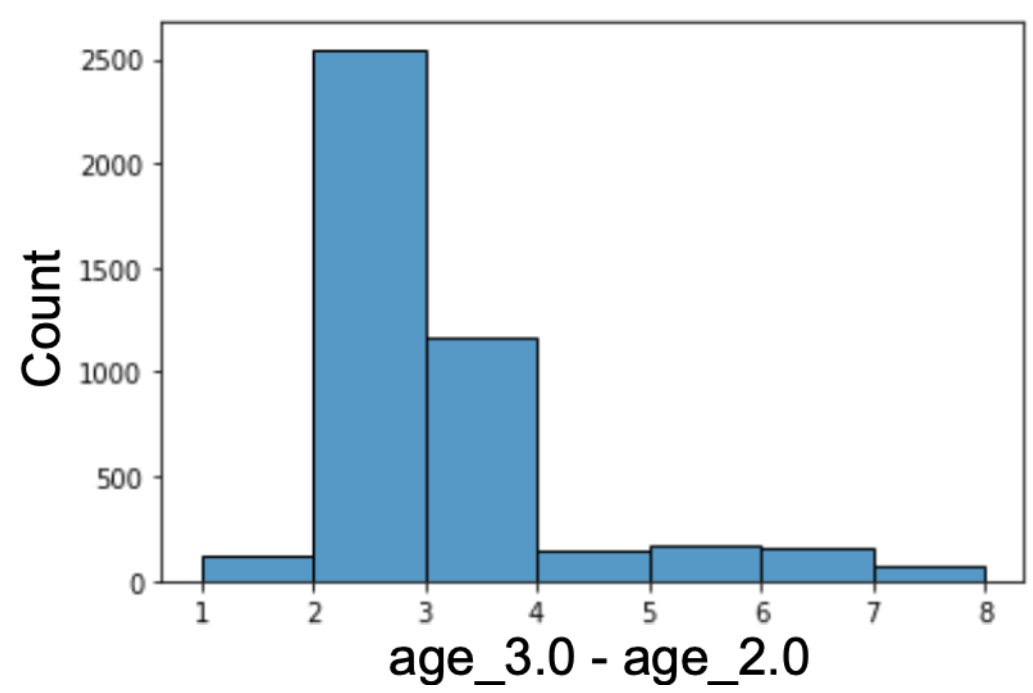

Supplementary Figure 3. Histogram showing the time intervals (in years) between the first and second scan among participants who underwent repeat imaging. Age_2.0 recorded the age at the first scan session, and age_3.0 recorded the age at the second scan session. Age_2.0 recorded the age at the first scan session; age_3.0 recorded the age at the second scan session. The interval was calculated as age_3.0 minus age_2.0 for each participant. Bin width = 1 year. n = 3290 independent participants with repeat imaging after imaging quality check. Source data are provided as a Source Data file.

We assessed scan-to-scan consistency using Spearman's rank correlation coefficient, stratifying results by the time interval between the two scans (x-axis) (Supplementary Figure 4). We observed age-related degeneration in spinal cord cross-sectional area and diameters, as indicated by decreasing correlations with increasing scan intervals. In contrast, eccentricity measurements showed consistently high correlations regardless of the age gap, suggesting greater stability of this phenotype over time.

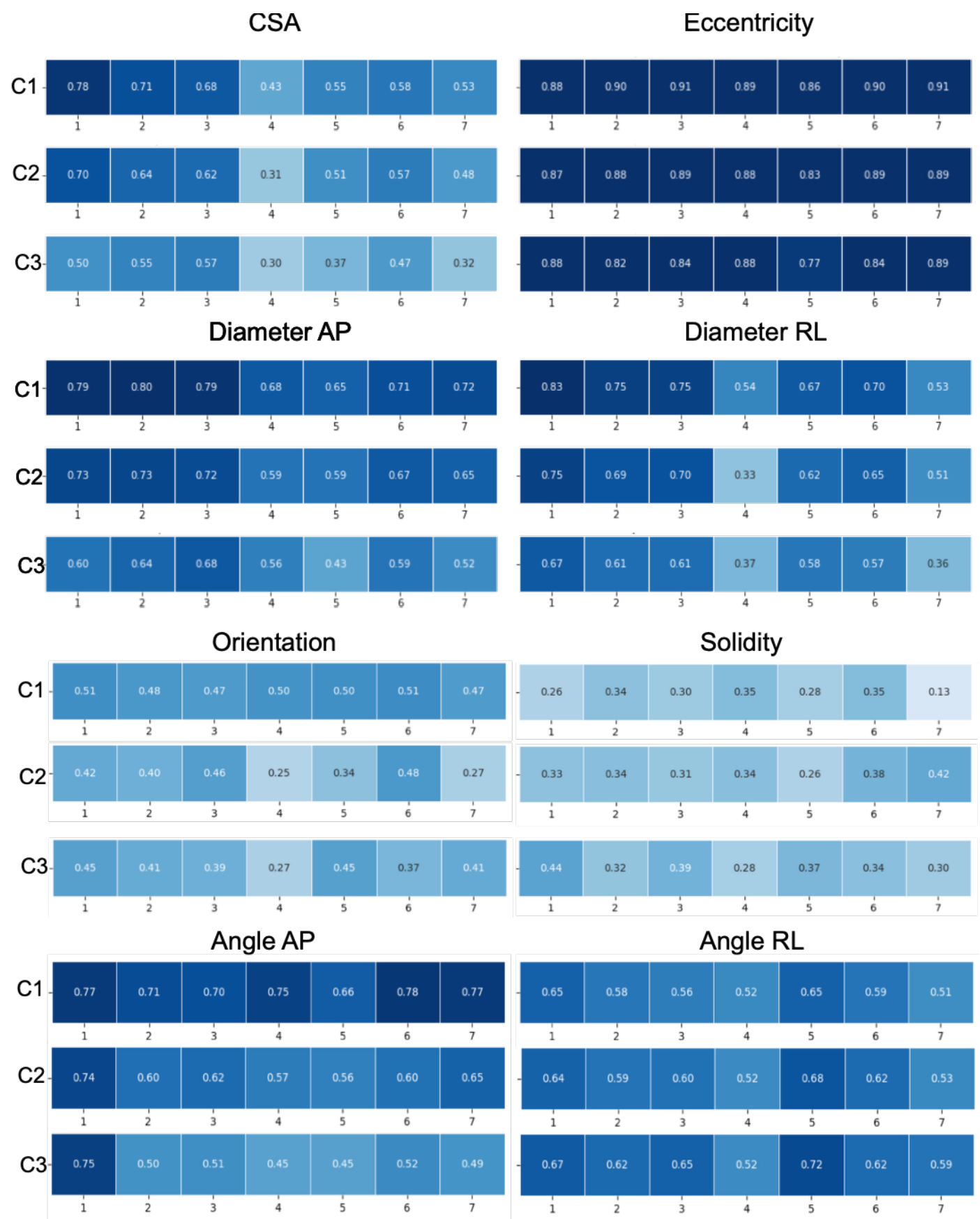

Supplementary Figure 4. Heatmaps of the Spearman's rank correlation coefficients between the first and second scan for each spinal cord phenotype. Correlation values are plotted against the between scans intervals (years). Cell colour intensity reflects the magnitude of the correlation coefficient (dark blue, higher; light blue, lower), with the numeric value overlaid on each cell. Source data are provided as a Source Data file. n = 3290 independent participants with repeat imaging after imaging quality check.

2.2 Evaluation of method robustness: atlas-based vs. native-space analysis

2.2.1 Alternative processing method

To assess the robustness of spinal cord metrics, we compared two processing pipelines: native-space and atlas-based analysis (Supplementary Figure 5). While the current study used the native-space approach, the atlas-based method was evaluated as an alternative to ensure the spinal cord shape metrics can be reliably extracted across processing pipelines. Both approaches began with segmentation and vertebral labelling of C1–C3. In the native-space method, metrics were extracted directly from the labelled segmentation (seg_labeled.nii). In contrast, the atlas-based pipeline included an additional registration step to using the PAM50 atlas[2], with metrics derived from the warped atlas labels (PAM50_levels.nii.gz).

Image registration was performed using vertebral labels from the deep learning model. The centres of C2 and C3 were used to guide the affine transformation. Two-point registration was preferred over three-point registration in this case because the latter would require a non-affine transform and is error-prone due to degraded image quality at lower levels. C2 and C3 were the most accurately labelled regions and provided robust reference for alignment. However, since C1 was not used in registration, its label remains similar to those generated by SCT's sct_label_vertebrae command, not benefiting from the trained vertebral labelling model. This comparison in a way describes an improvement of the C1 labelling. For implementation details, please refer to https://github.com/art2mri/Enigma-SC.

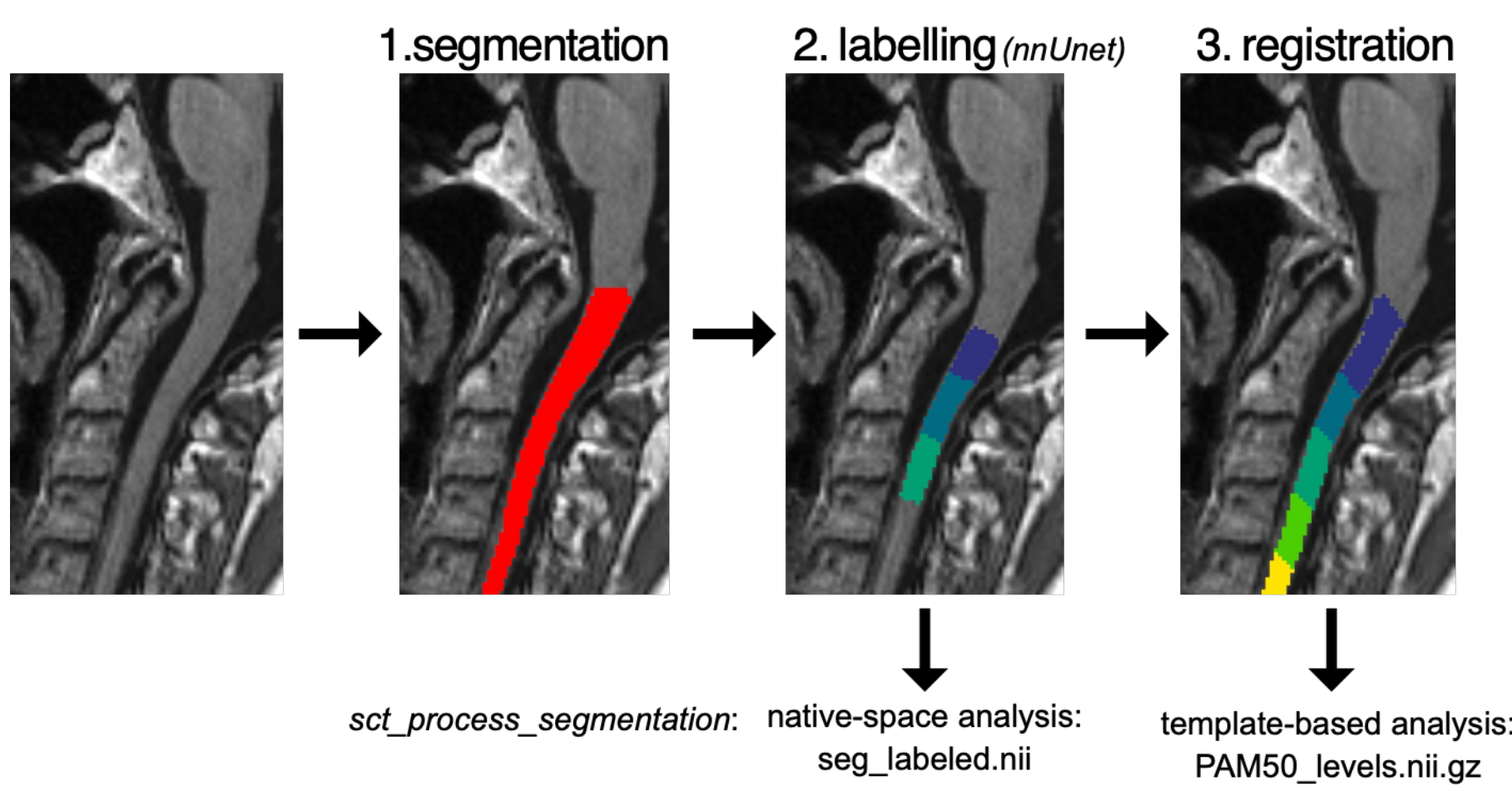

Supplementary Figure 5. Comparison of native-space vs. atlas-based spinal cord analysis pipelines.

2.2.2 Metric agreement between processing methods

SCT shape analysis outputs nine shape metrics: cross-sectional area, diameter AP, diameter RL, eccentricity, orientation, solidity, sum length, angle AP, and angle RL. Supplementary Figure 6 shows violin plots for each of these nine phenotypes at the C1–C3 vertebral levels, along with scatter plots comparing measurements derived from the two processing choices. Spearman's rank correlation (R^2) is reported to quantify the agreement between methods. All method testing in the following sections used images that passed only this initial visual inspection of seg_labeled.nii

images. Additional errors were deliberately retained to enable better evaluation, comparison, and optimisation of the pipeline.

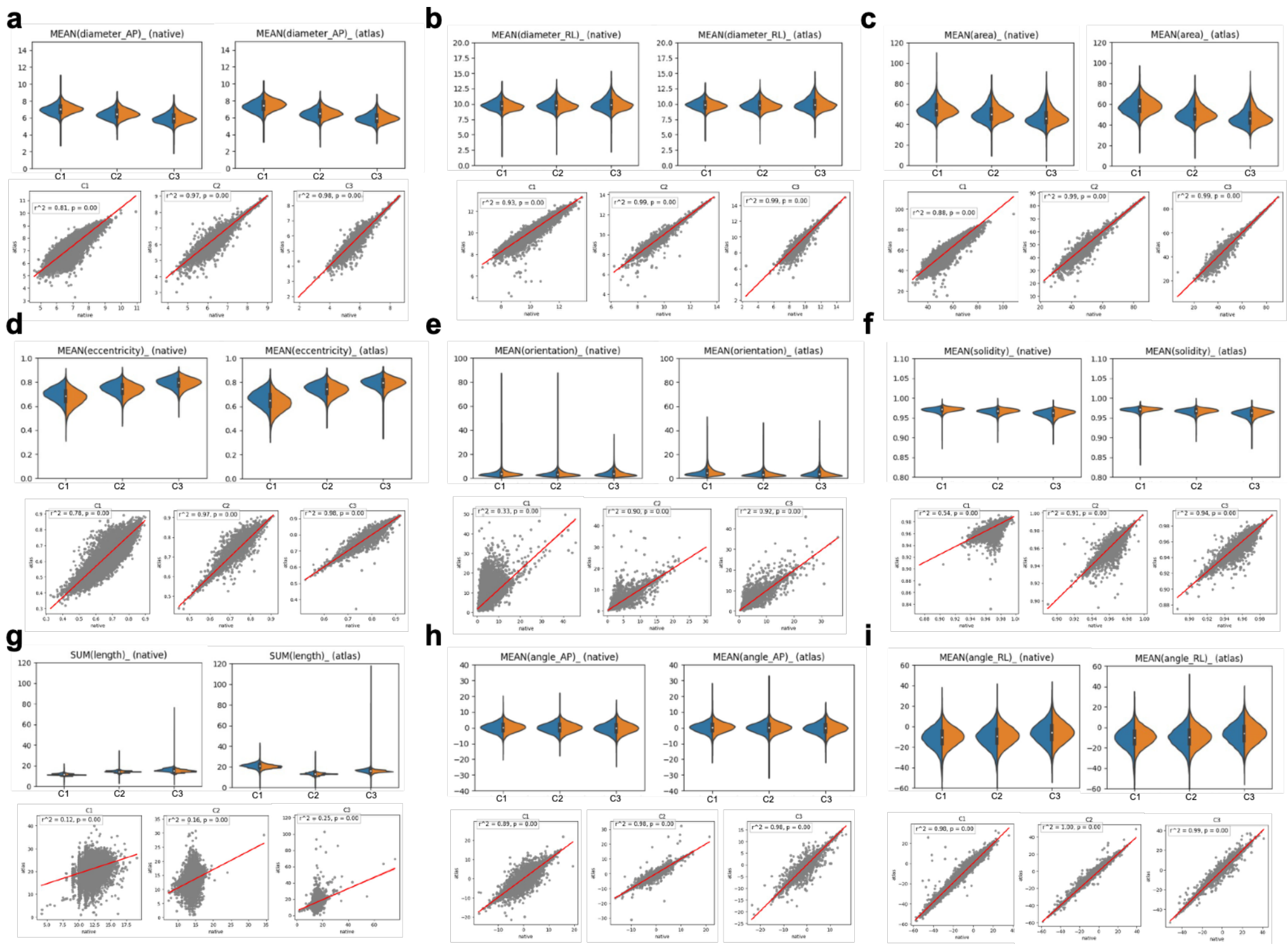

Supplementary Figure 6. Violin plots of all nine SCT shape metrics including (a) diameter AP, (b) diameter RL, (c) cross-sectional area, (d) eccentricity, (e) orientation, (f) solidity, (g) sum length, (h) angle AP, and (i) angle RL at vertebral levels C1–C3 (left), and scatter plots below showing the scatterplots of the metrics derived by the two processing pipelines. Spearman's R^2 values and p-values are annotated in each panel.

C1 measurements were noticeably different between processing methods across several metrics. Sum length showed poor agreement, with a Spearman's correlation of only 0.12 between atlas-based and native-space analyses (Supplementary Figure 6g), suggesting that the two methods identified substantially different regions. A known issue is the unreliable detection of C1, which often leads to over-segmentation where C1 spans the anterior portion of the entire spinal cord mask (Supplementary Figure 7). When compared to relative spinal segment lengths previously reported in human anatomical studies: 1.6%, 2.2%, and 3.5% of total spinal cord length for C1, C2, and C3 respectively[2]. The atlas-based method produced a C1 length almost double that of C2, while the native-space approach aligned more closely with anatomical references. Orientation and solidity also showed poor agreement, with R^2 values of only 0.33 and 0.54, respectively (Supplementary Figure 6e-f). Diameters, cross-sectional area, and eccentricity showed moderate to high agreement in C1, with R^2 ranging from 0.78 to 0.93 (Supplementary Figure 6 a-d).

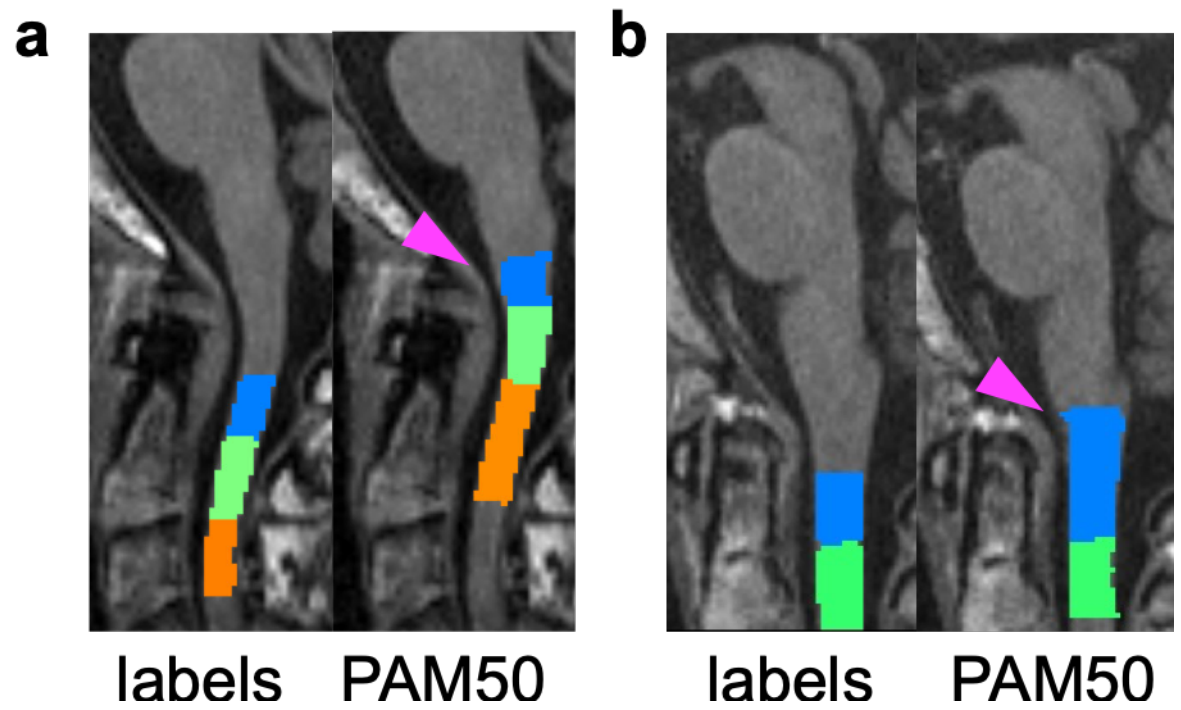

Supplementary Figure 7. Examples of C1 labelling using the native vs. atlas-based analysis. (a) inaccurate C1 detection (b) over segmentation of C1.

We found strong consistency between native-space and atlas-based methods at the C2 and C3 levels (Supplementary Figure 4). Cross-sectional area, diameter AP, diameter RL, eccentricity, angle AP, and angle RL had very high agreement ($R^2 = 0.97–0.99$), indicating these traits are highly consistent across processing methods in C2 and C3 (Supplementary Figure 6 a-d, h-i). Orientation and solidity had slightly lower correlations ($R^2 = 0.90–0.94$), suggesting some variability between the two methods (Supplementary Figure 6 e-f).

2.2.3 Registration errors

Another limitation of the atlas-based analysis is registration errors, particularly in images with poorer quality, which resulted in discontinuous spinal cord segmentation masks (Supplementary Figure 8b). Although these discontinuities are often corrected during the vertebral labelling step, errors may still propagate into the subsequent atlas registration. We examined the correlation between C1 cross-sectional area (CSA) from native-space and atlas-based analyses (Supplementary Figure 8a). Samples with large residuals were manually reviewed, and the majority were confirmed to have registration issues that led to inaccurate phenotype estimates.

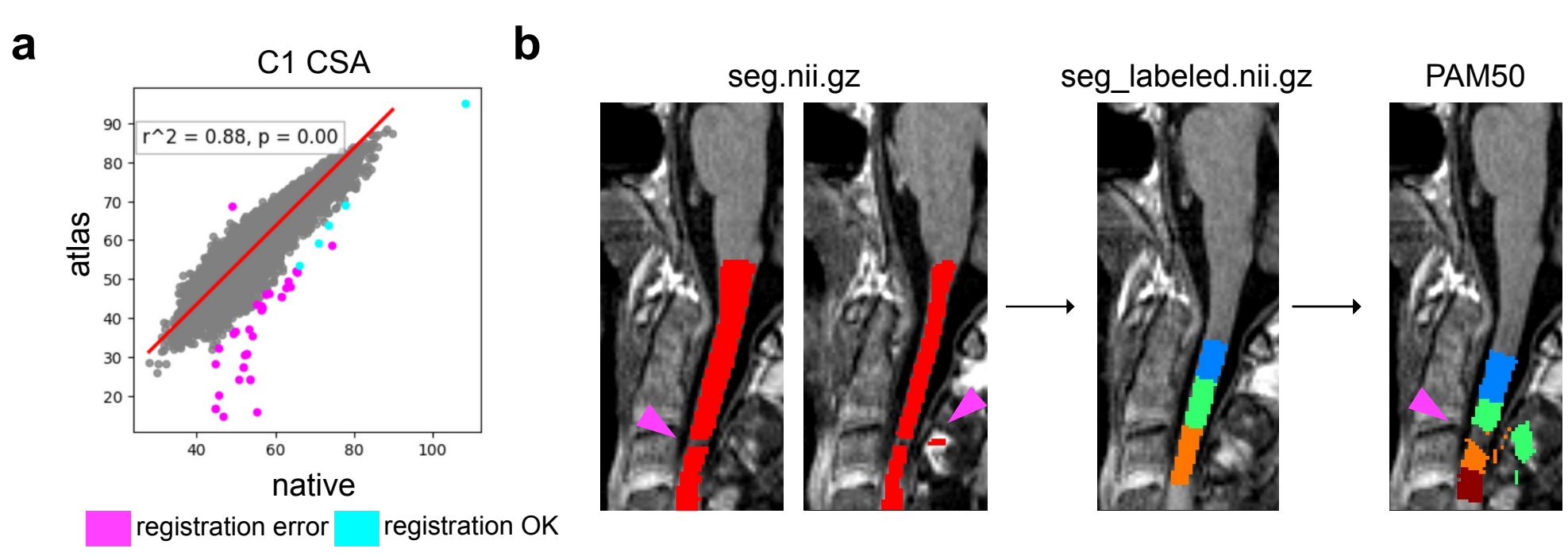

Supplementary Figure 8. Registration errors in atlas-based spinal cord analysis. (a) Spearman's correlation of C1 cross-sectional area between native-space and atlas-based analyses. Points with large residuals ($|z|/std(z) > 5$, $n = 34$) were manually reviewed; magenta points highlight confirmed registration errors ($n = 29$). (b) Example of a registration failure resulting in anatomically incorrect atlas warping.

Section 3: Genome-wide association (GWA) results in the European ancestry

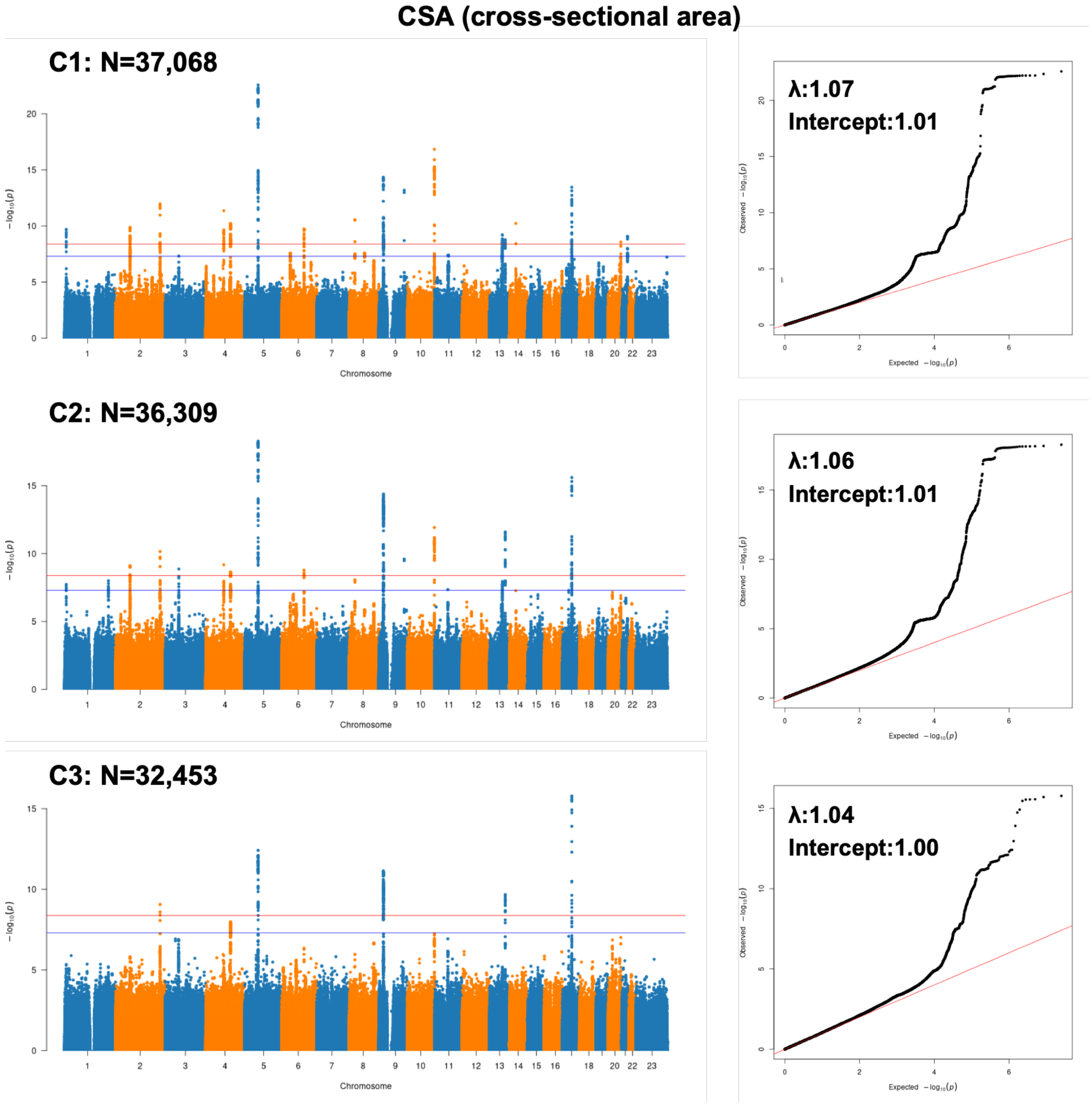

Supplementary Figure 9. Manhattan and quantile–quantile (Q–Q) plots for cross-sectional area at the C1, C2, and C3 vertebral levels in the European sample. In Manhattan plots, each point represents a single genetic variant according to its genomic position (x-axis) and $-\log_{10}P$ (two-sided P values). The blue horizontal line indicates the conventional genome-wide significance threshold ($P < 5 \times 10^{-8}$), while the red line marks the Bonferroni-corrected threshold used in this study ($P < 4.2 \times 10^{-9}$), accounting for 12 phenotypes. In the Q–Q plots, the red diagonal line represents the expected null distribution. Genomic inflation factors (λ) and LD Score regression intercepts are labelled within each plot. This analysis included n = 37, 068 (C1), n = 36,309 (C2), and n = 32,453 (C3) independent UK Biobank participants.

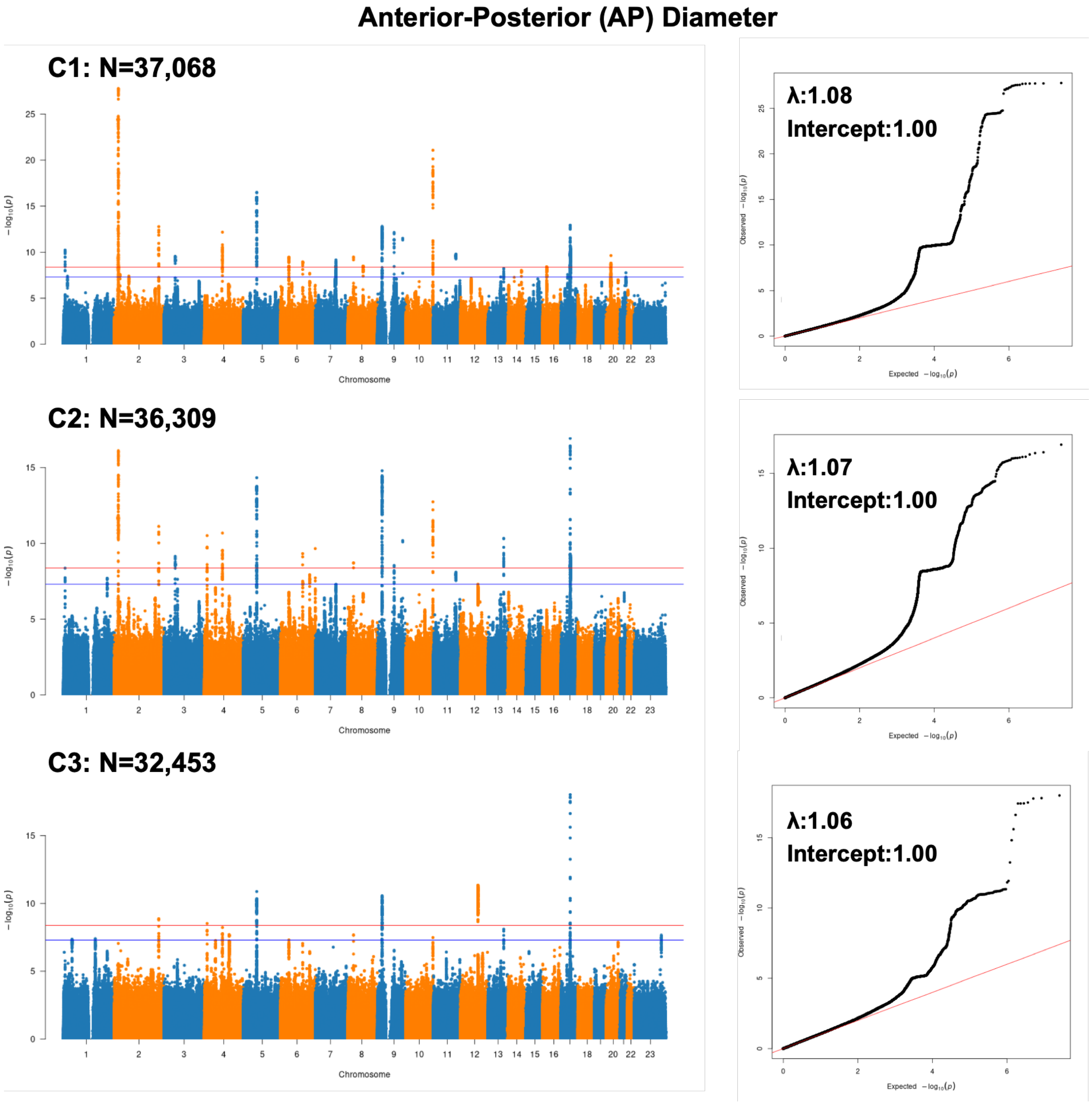

Supplementary Figure 10. Manhattan and quantile–quantile (Q–Q) plots for anterior-posterior diameter at the C1, C2, and C3 vertebral levels in the European sample. In Manhattan plots, each point represents a single genetic variant according to its genomic position (x-axis) and $-\log_{10}$P (two-sided P values). The blue horizontal line indicates the conventional genome-wide significance threshold ($P < 5 \times 10^{-8}$), while the red line marks the Bonferroni-corrected threshold used in this study ($P < 4.2 \times 10^{-9}$), accounting for 12 phenotypes. In the Q–Q plots, the red diagonal line represents the expected null distribution. Genomic inflation factors (λ) and LD Score regression intercepts are labelled within each plot. This analysis included n = 37, 068 (C1), n = 36,309 (C2), and n = 32,453 (C3) independent UK Biobank participants.

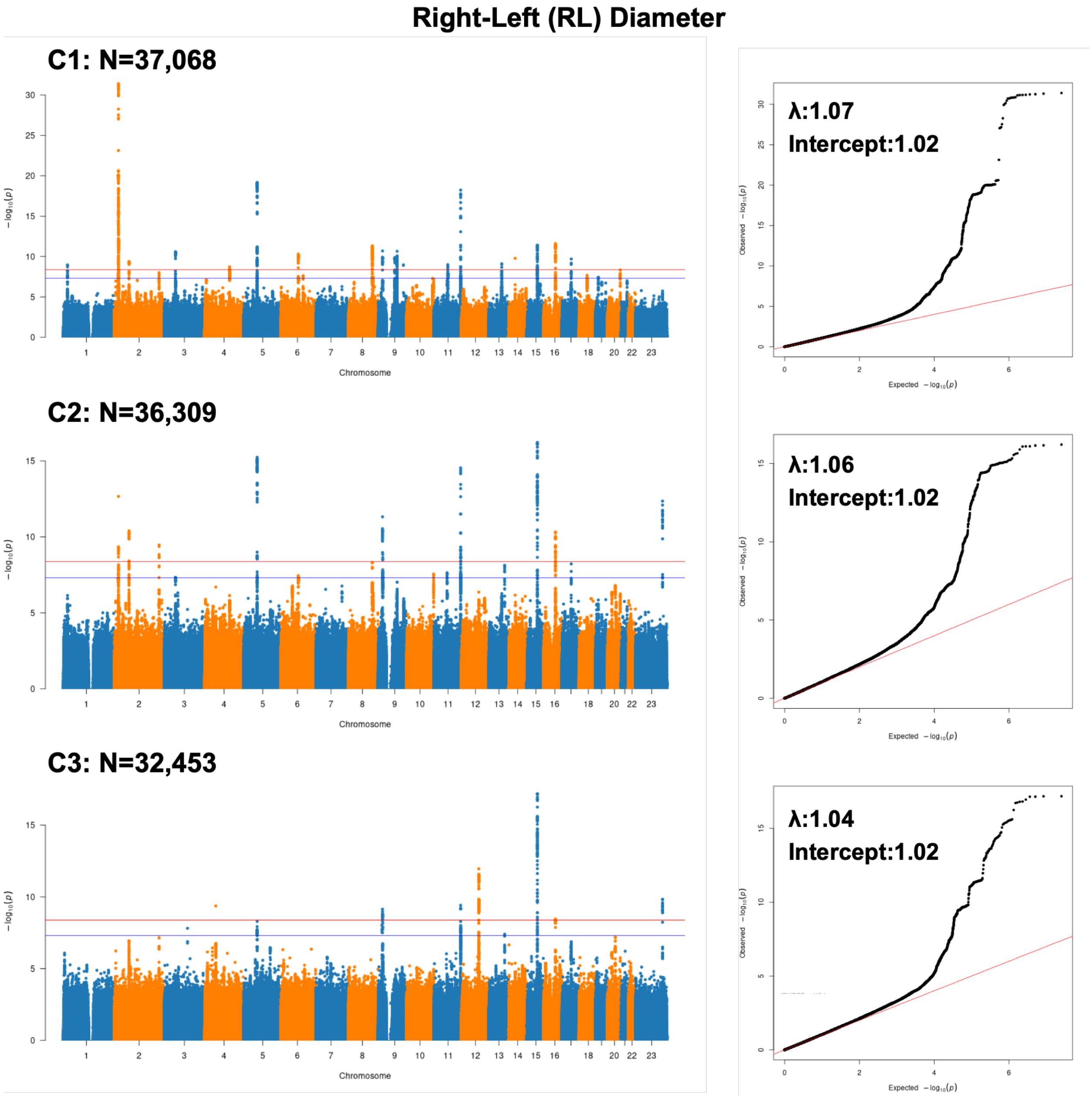

Supplementary Figure 11. Manhattan and quantile–quantile (Q–Q) plots for right-left diameter at the C1, C2, and C3 vertebral levels in the European sample. In Manhattan plots, each point represents a single genetic variant according to its genomic position (x-axis) and $-\log_{10}P$ (two-sided P values). The blue horizontal line indicates the conventional genome-wide significance threshold ($P < 5 \times 10^{-8}$), while the red line marks the Bonferroni-corrected threshold used in this study ($P < 4.2 \times 10^{-9}$), accounting for 12 phenotypes. In the Q–Q plots, the red diagonal line represents the expected null distribution. Genomic inflation factors (λ) and LD Score regression intercepts are labelled within each plot. This analysis included n = 37, 068 (C1), n = 36,309 (C2), and n = 32,453 (C3) independent UK Biobank participants.

Eccentricity

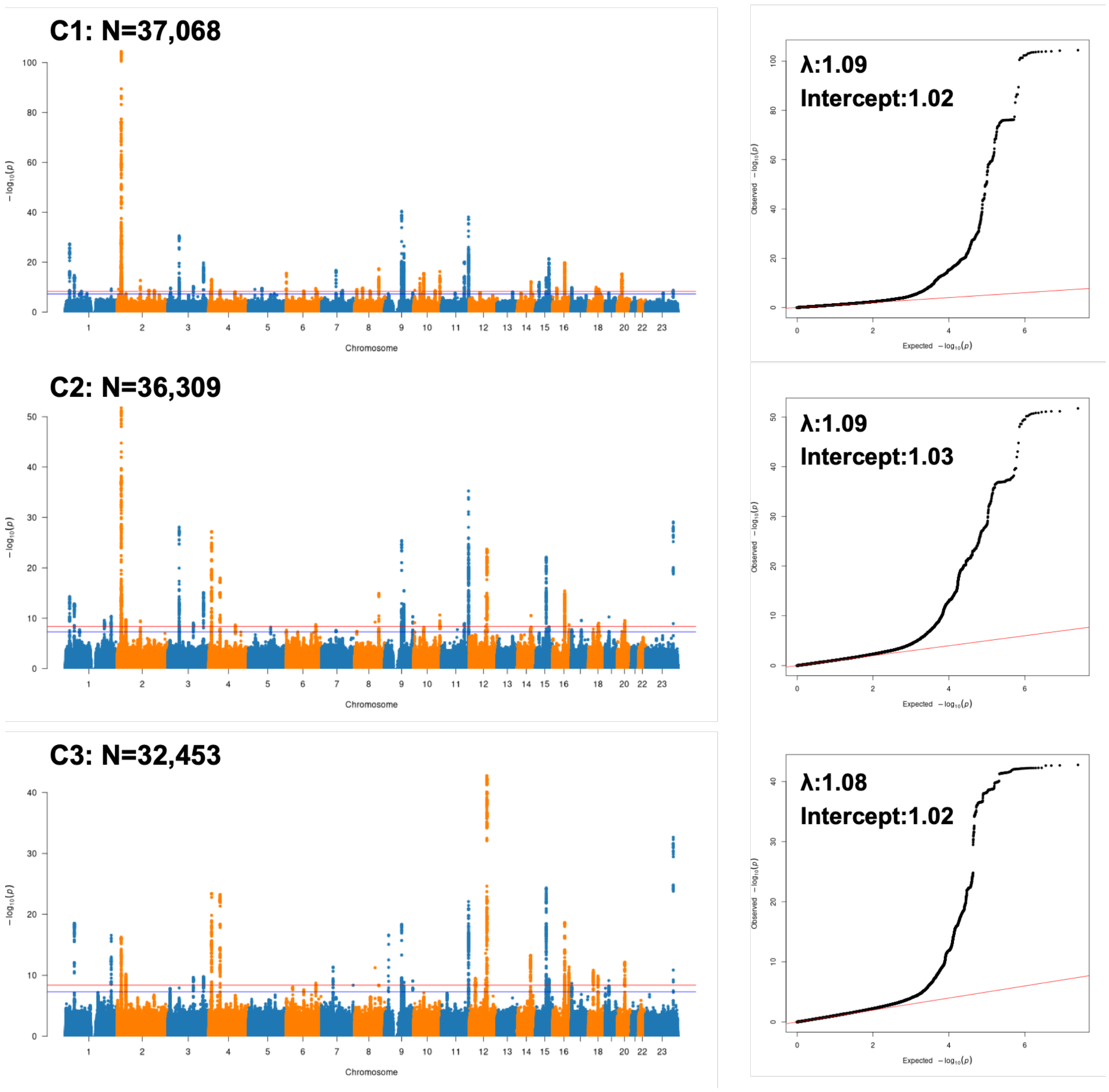

Supplementary Figure 12. Manhattan and quantile–quantile (Q–Q) plots for **eccentricity** at the C1, C2, and C3 vertebral levels in the European sample. In Manhattan plots, each point represents a single genetic variant according to its genomic position (x-axis) and $-\log_{10}P$ (two-sided P values). The blue horizontal line indicates the conventional genome-wide significance threshold ($P < 5 \times 10^{-8}$), while the red line marks the Bonferroni-corrected threshold used in this study ($P < 4.2 \times 10^{-9}$), accounting for 12 phenotypes. In the Q–Q plots, the red diagonal line represents the expected null distribution. Genomic inflation factors (λ) and LD Score regression intercepts are labelled within each plot. This analysis included n = 37, 068 (C1), n = 36,309 (C2), and n = 32,453 (C3) independent UK Biobank participants.

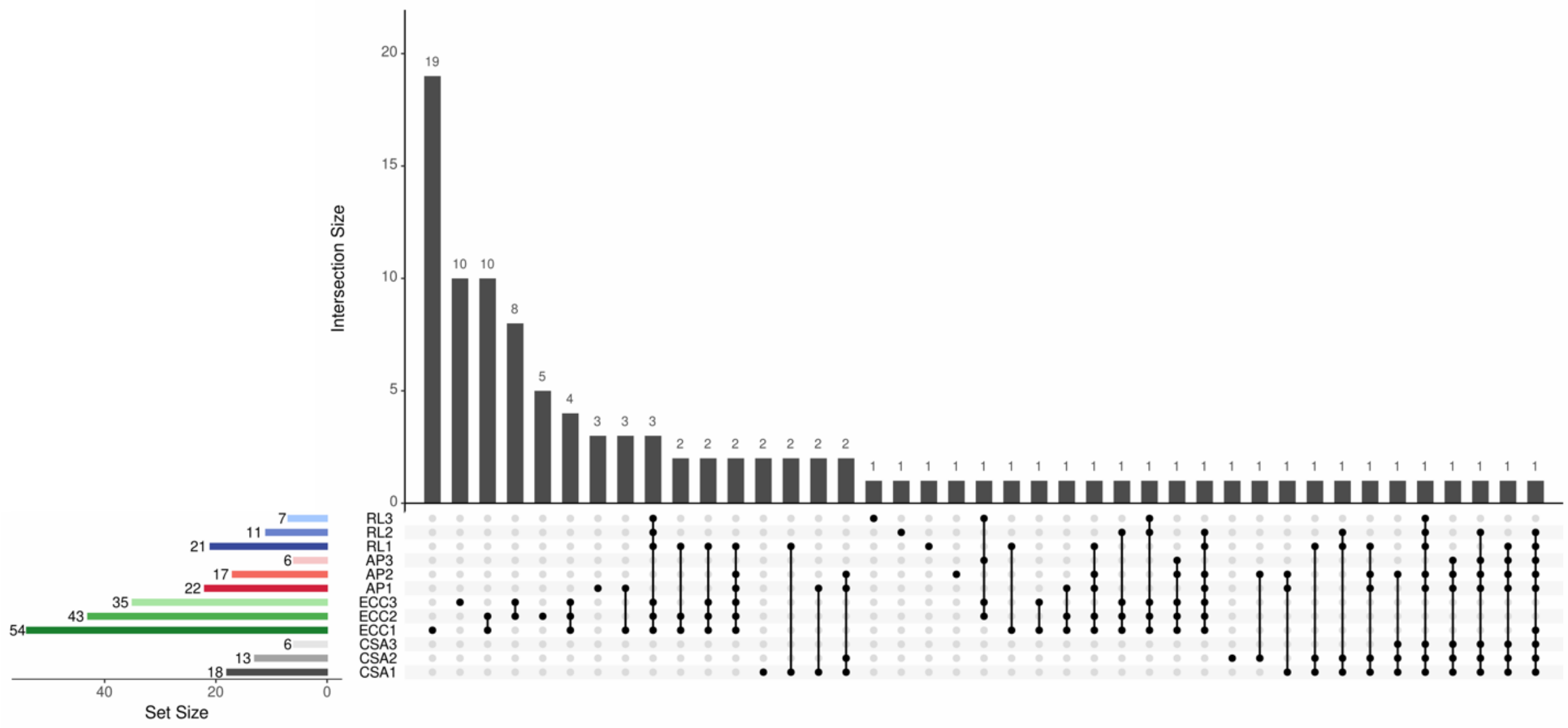

Supplementary Figure 13. Summary of loci identified for 12 spinal cord shape metrics. Loci were defined by merging independent SNPs with $P < 4.17 \times 10^{-9}$ that were within 250 kilobases of each other and had pairwise linkage disequilibrium $R^2 > 0.1$. A full list of the independent loci is included in Supplementary Data 2.

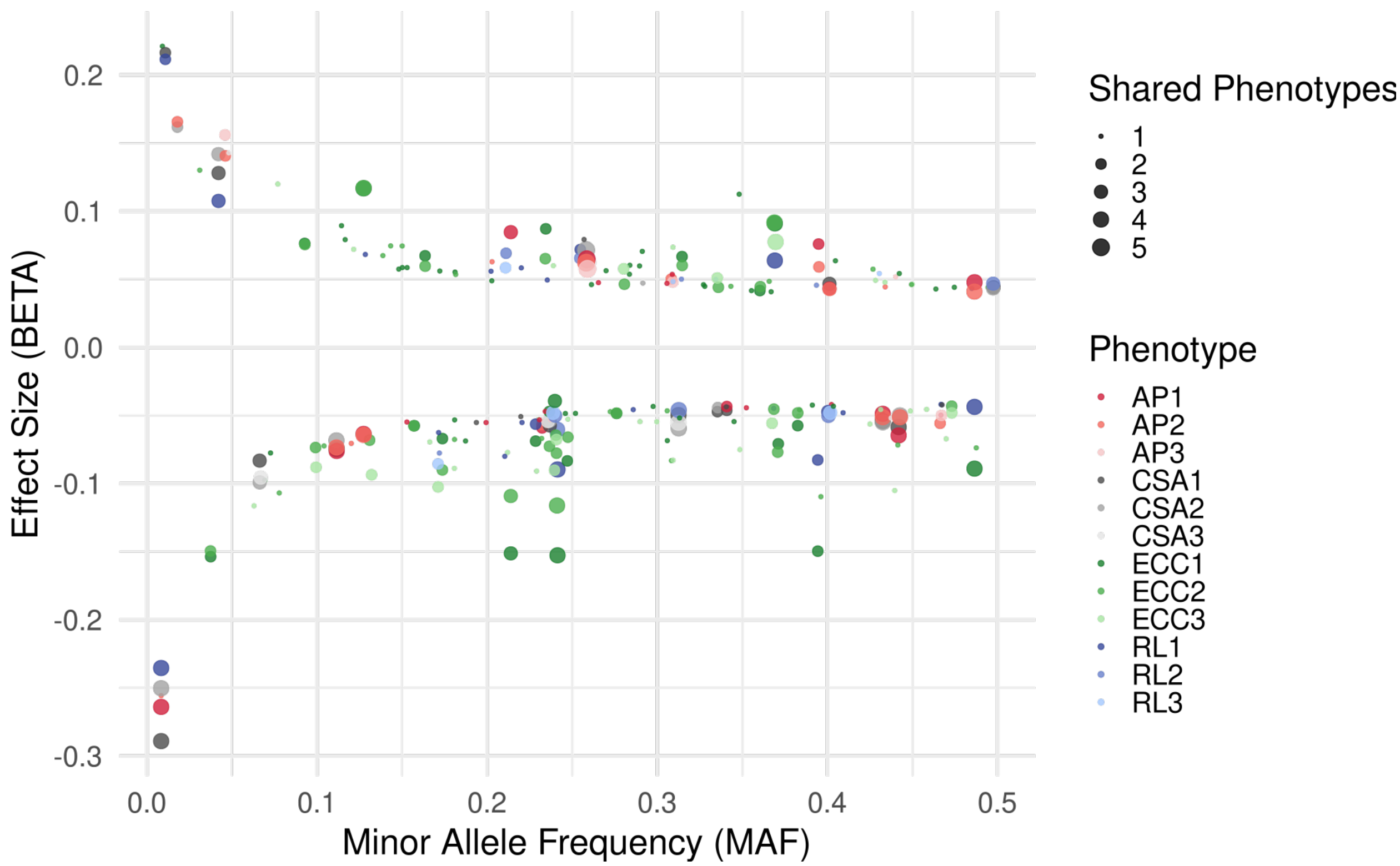

Supplementary Figure 14. Minor allele frequency (MAF) versus effect size (BETA) for genome-wide significant variants. Each point represents a variant that reached genome-wide significance ($P < 4.2 \times 10^{-9}$) in the GWAS. Shapes indicate whether SNPs are shared across multiple phenotypes, colors represent the four spinal cord shape metrics, and shading reflects the vertebral level (C1 to C3). The exact MAF and BETA values are in Supplementary Data 2.

Section 4: GWAS covariate selection using stepwise regression

To validate the choice of covariates, we performed stepwise regression guided by principal component analysis (PCA). PCA was conducted on the four selected spinal cord phenotypes (cross-sectional area (CSA), eccentricity, anterior-posterior diameter, right-left diameter) separately for each vertebral level (C1–C3). Across all levels, the first two principal components (PC1 and PC2) accounted for over 99% of the variance. PC1 was primarily driven by CSA and diameter measures, while PC2 was mainly influenced by eccentricity and diameter measures. We tested sex, age, height, intracranial volume (ICV), imaging site, and T1-weighted signal-to-noise ratio (SNR) as candidate covariates in stepwise regression models and selected the best-fitting model based on the lowest Akaike Information Criterion (AIC). Results indicated that all tested covariates, except for imaging site, were significant contributors. However, imaging site was still included as a routine covariate to account for potential batch effects in GWAS.

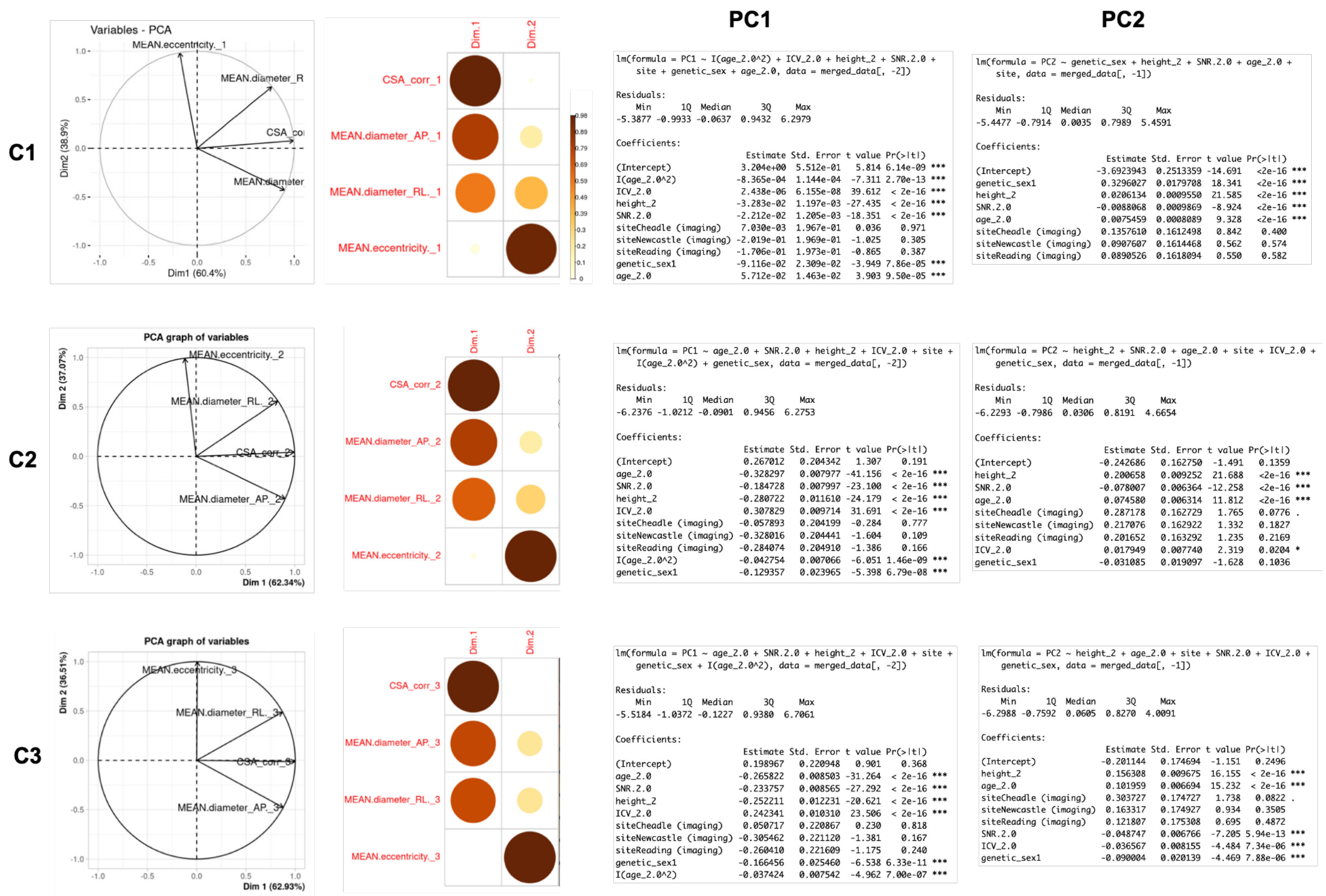

Supplementary Figure 15: Principal component analysis (PCA) of four spinal cord phenotypes (cross-sectional area (CSA), eccentricity, anterior-posterior diameter, right-left diameter) at each vertebral level (C1–C3). The plots show the correlations of PC1 and PC2 with each phenotype. Summary of covariates selected through stepwise regression based on models with the lowest AIC.

Section 5: GWA results in the European ancestry (Excluded height as a covariate)

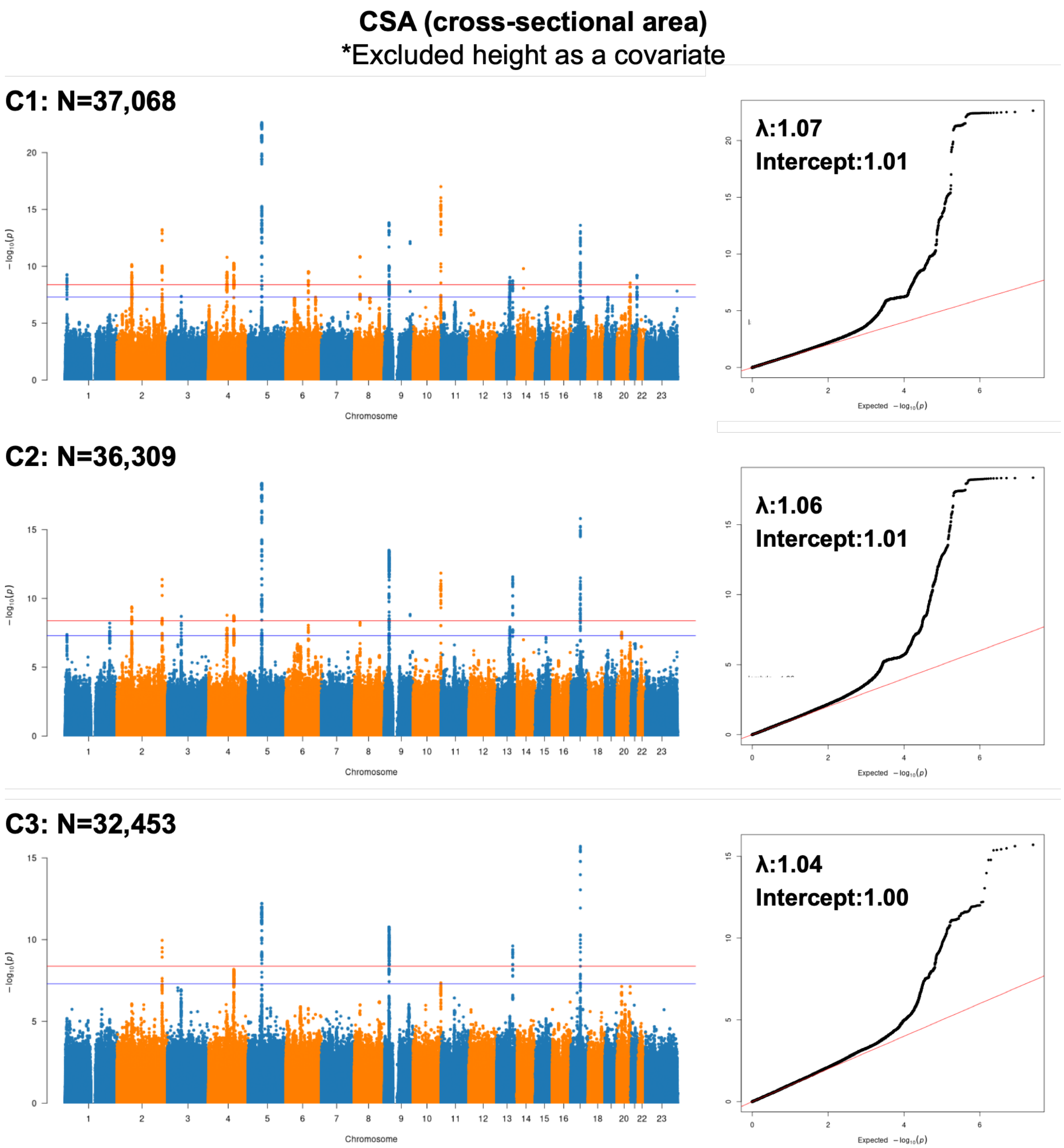

Supplementary Figure 16. Manhattan and quantile–quantile (Q–Q) plots for cross-sectional area at the C1, C2, and C3 vertebral levels in the European sample, without height as a covariate. In Manhattan plots, each point represents a single genetic variant according to its genomic position (x-axis) and $-\log_{10}P$ (two-sided P values). The blue horizontal line indicates the conventional genome-wide significance threshold ($P < 5 \times 10^{-8}$), while the red line marks the Bonferroni-corrected threshold used in this study ($P < 4.2 \times 10^{-9}$), accounting for 12 phenotypes. In the Q–Q plots, the red diagonal line represents the expected null distribution. Genomic inflation factors (λ) and LD Score regression intercepts are labelled within each plot. This analysis included n = 37, 068 (C1), n = 36,309 (C2), and n = 32,453 (C3) independent UK Biobank participants.

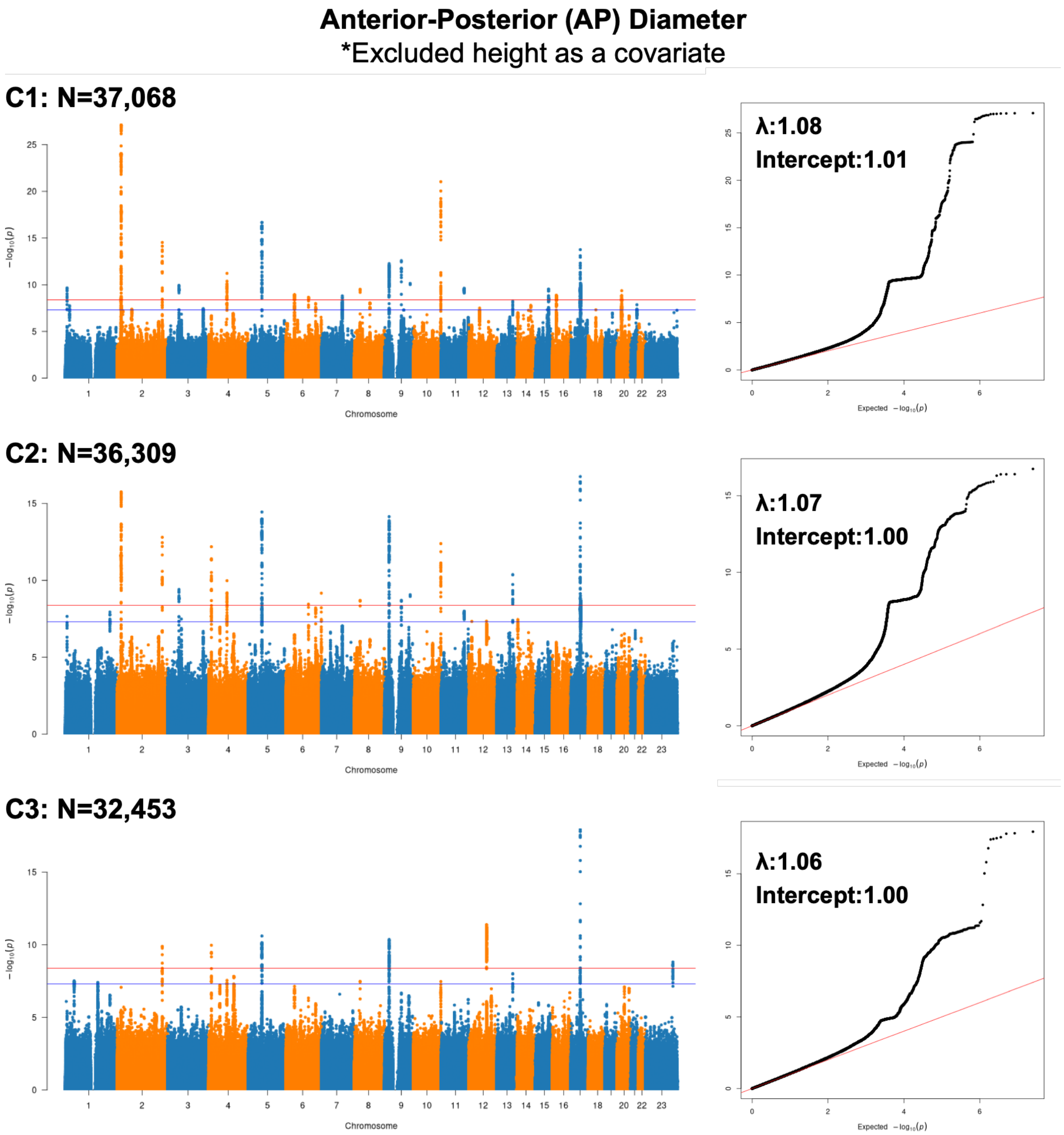

Supplementary Figure 17. Manhattan and quantile–quantile (Q–Q) plots for anterior-posterior diameter at the C1, C2, and C3 vertebral levels in the European sample, without height as a covariate. In Manhattan plots, each point represents a single genetic variant according to its genomic position (x-axis) and $-\log_{10}P$ (two-sided P values). The blue horizontal line indicates the conventional genome-wide significance threshold ($P < 5 \times 10^{-8}$), while the red line marks the Bonferroni-corrected threshold used in this study ($P < 4.2 \times 10^{-9}$), accounting for 12 phenotypes. In the Q–Q plots, the red diagonal line represents the expected null distribution. Genomic inflation factors (λ) and LD Score regression intercepts are labelled within each plot. This analysis included n = 37, 068 (C1), n = 36,309 (C2), and n = 32,453 (C3) independent UK Biobank participants.

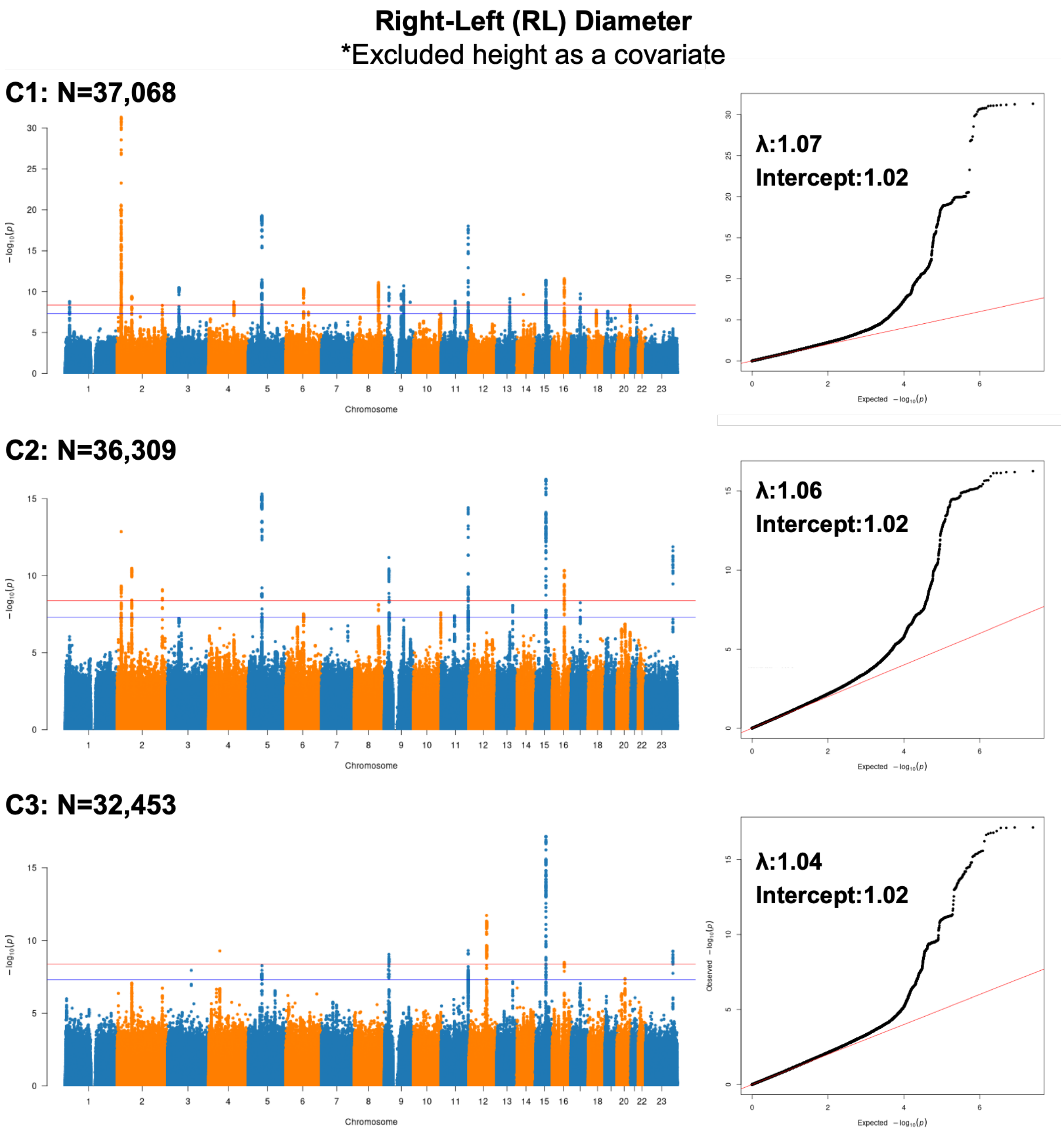

Supplementary Figure 18. Manhattan and quantile–quantile (Q–Q) plots for right-left diameter at the C1, C2, and C3 vertebral levels in the European sample, without height as a covariate. In Manhattan plots, each point represents a single genetic variant according to its genomic position (x-axis) and $-\log_{10}P$ (two-sided P values). The blue horizontal line indicates the conventional genome-wide significance threshold ($P < 5 \times 10^{-8}$), while the red line marks the Bonferroni-corrected threshold used in this study ($P < 4.2 \times 10^{-9}$), accounting for 12 phenotypes. In the Q–Q plots, the red diagonal line represents the expected null distribution. Genomic inflation factors (λ) and LD Score regression intercepts are labelled within each plot. This analysis included n = 37, 068 (C1), n = 36,309 (C2), and n = 32,453 (C3) independent UK Biobank participants.

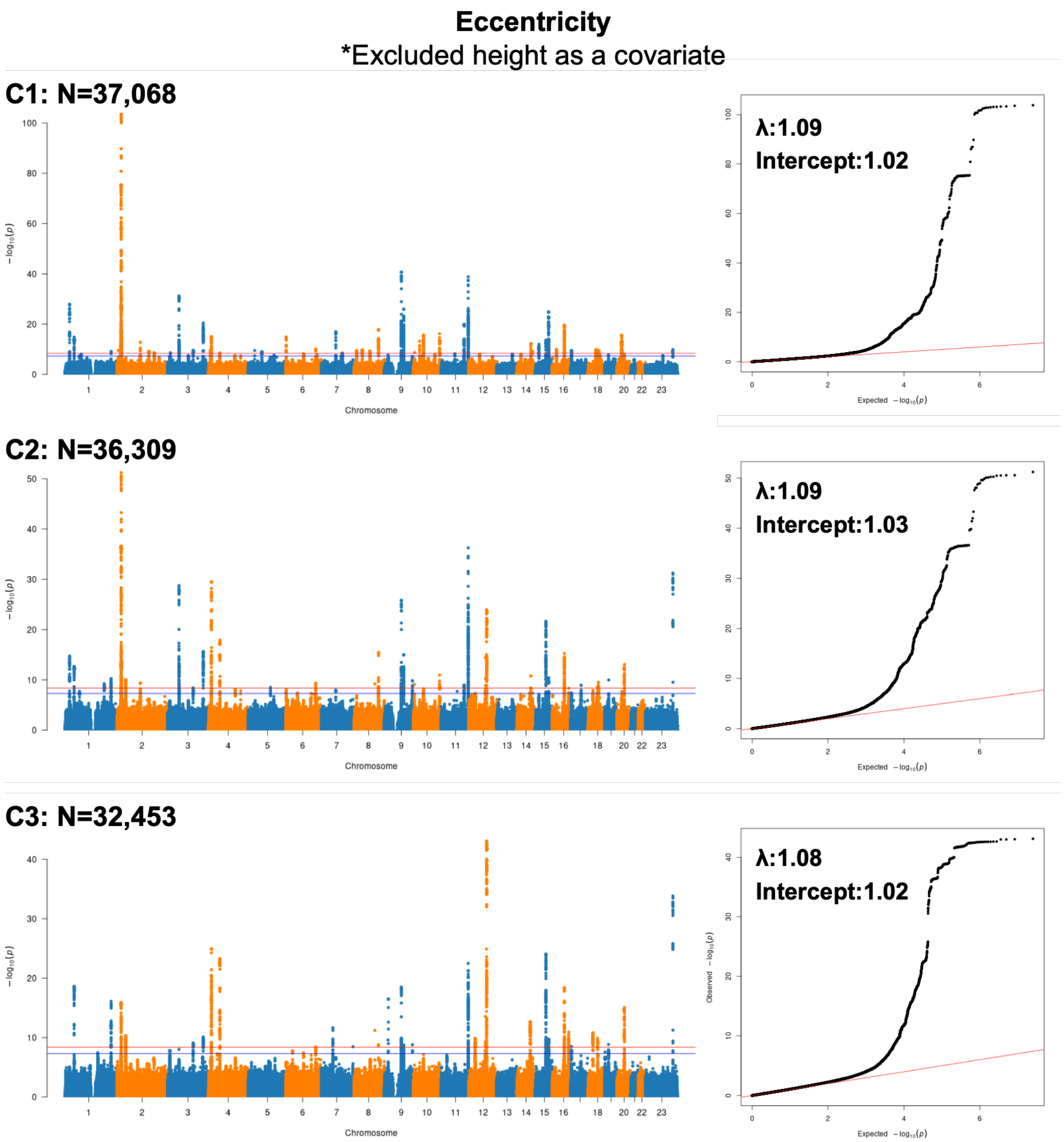

Supplementary Figure 19. Manhattan and quantile–quantile (Q–Q) plots for eccentricity at the C1, C2, and C3 vertebral levels in the European sample, without height as a covariate. In Manhattan plots, each point represents a single genetic variant according to its genomic position (x-axis) and $-\log_{10}P$ (two-sided P values). The blue horizontal line indicates the conventional genome-wide significance threshold ($P < 5 \times 10^{-8}$), while the red line marks the Bonferroni-corrected threshold used in this study ($P < 4.2 \times 10^{-9}$), accounting for 12 phenotypes. In the Q–Q plots, the red diagonal line represents the expected null distribution. Genomic inflation factors (λ) and LD Score regression intercepts are labelled within each plot. This analysis included n = 37, 068 (C1), n = 36,309 (C2), and n = 32,453 (C3) independent UK Biobank participants.

Section 6: Heritability sensitivity analysis for spinal cord shape metrics

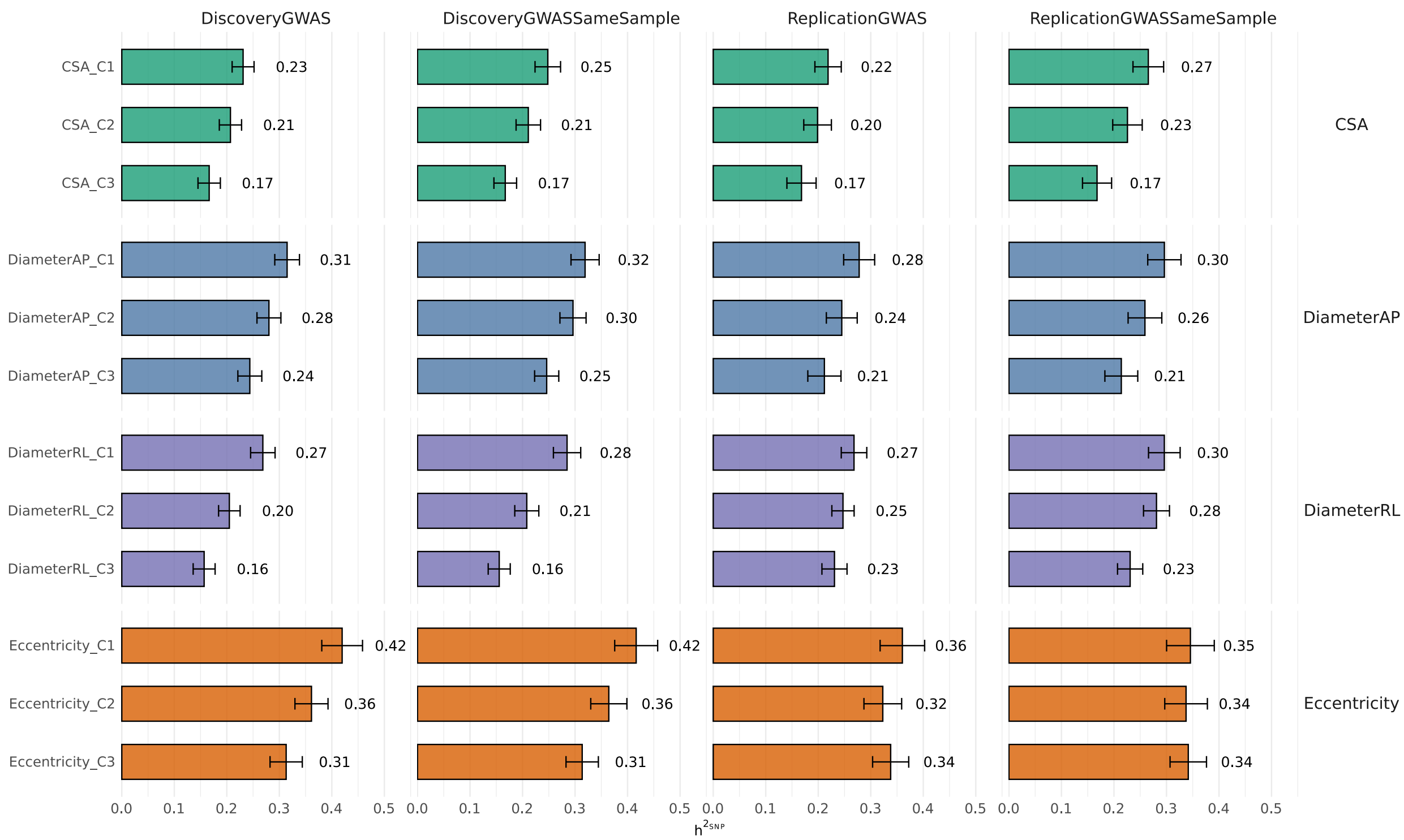

Supplementary Figure 20. Heritability estimation of spinal cord shape metrics. SNP-based heritability estimates for spinal cord shape metrics across four analyses: discovery, discovery restricted to the same sample (n=32,332), replication, and replication restricted to the same sample (n=22,366). SNP-heritability estimates are in Supplementary Data 7.

Section 7: Sex-specific GWAS results

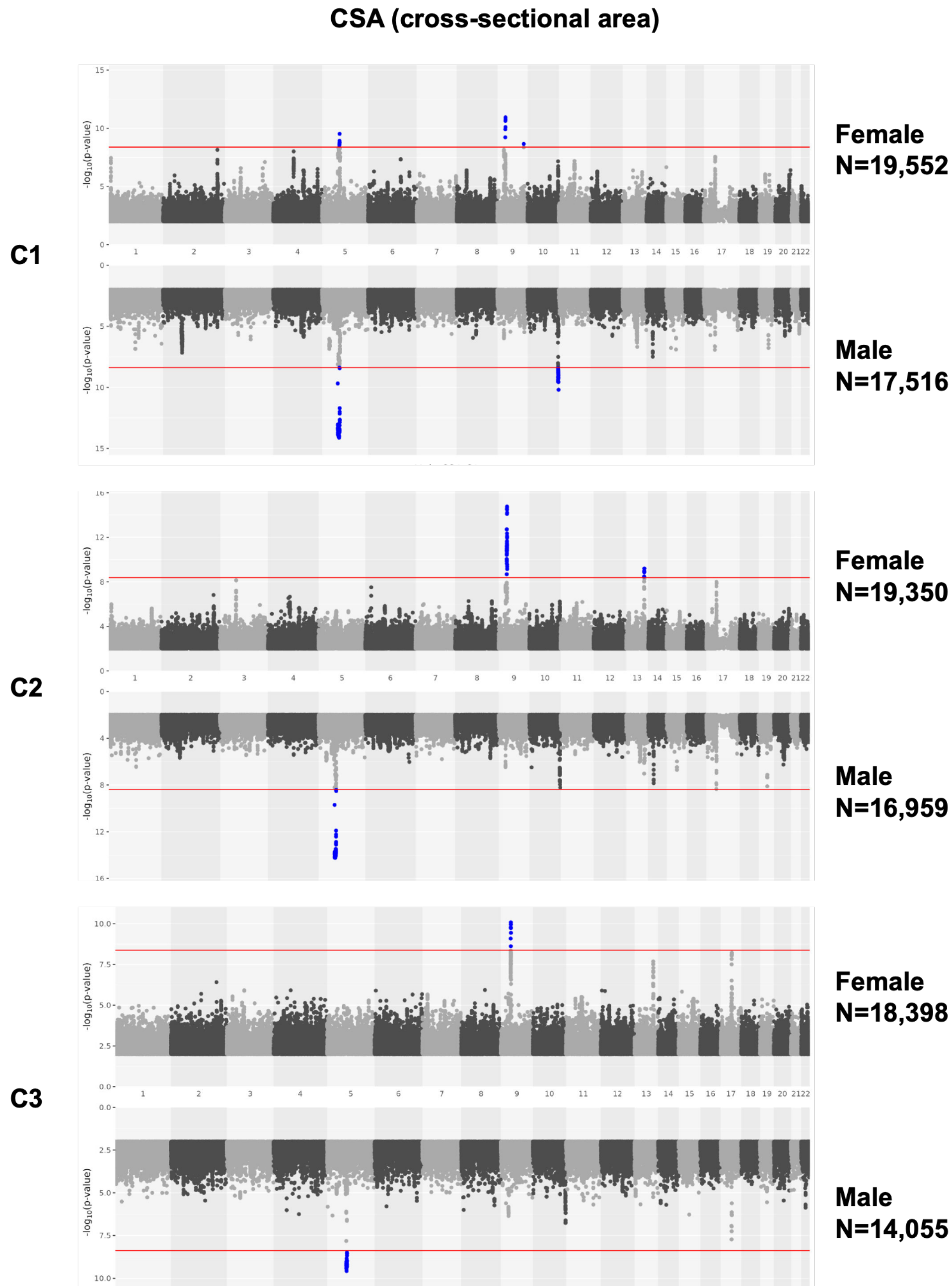

Supplementary Figure 21. Miami plot of sex-stratified GWAS for **cross-sectional area**. Results for females are shown in the upper panel and for males in the lower panel. The genome-wide significance threshold ($P < 4.2 \times 10^{-9}$) is indicated by the red horizontal line. Genome-wide significant variants are highlighted in blue. SNPs with $-\log_{10}(P) < 2$ are not displayed. Sex-stratified GWAS were performed at each vertebral level, with sample sizes of n = 19,552 females and n = 17,516 males at C1; n = 19,350 females and n = 16,959 males at C2; and n = 18,398 females and n = 14,055 males at C3.

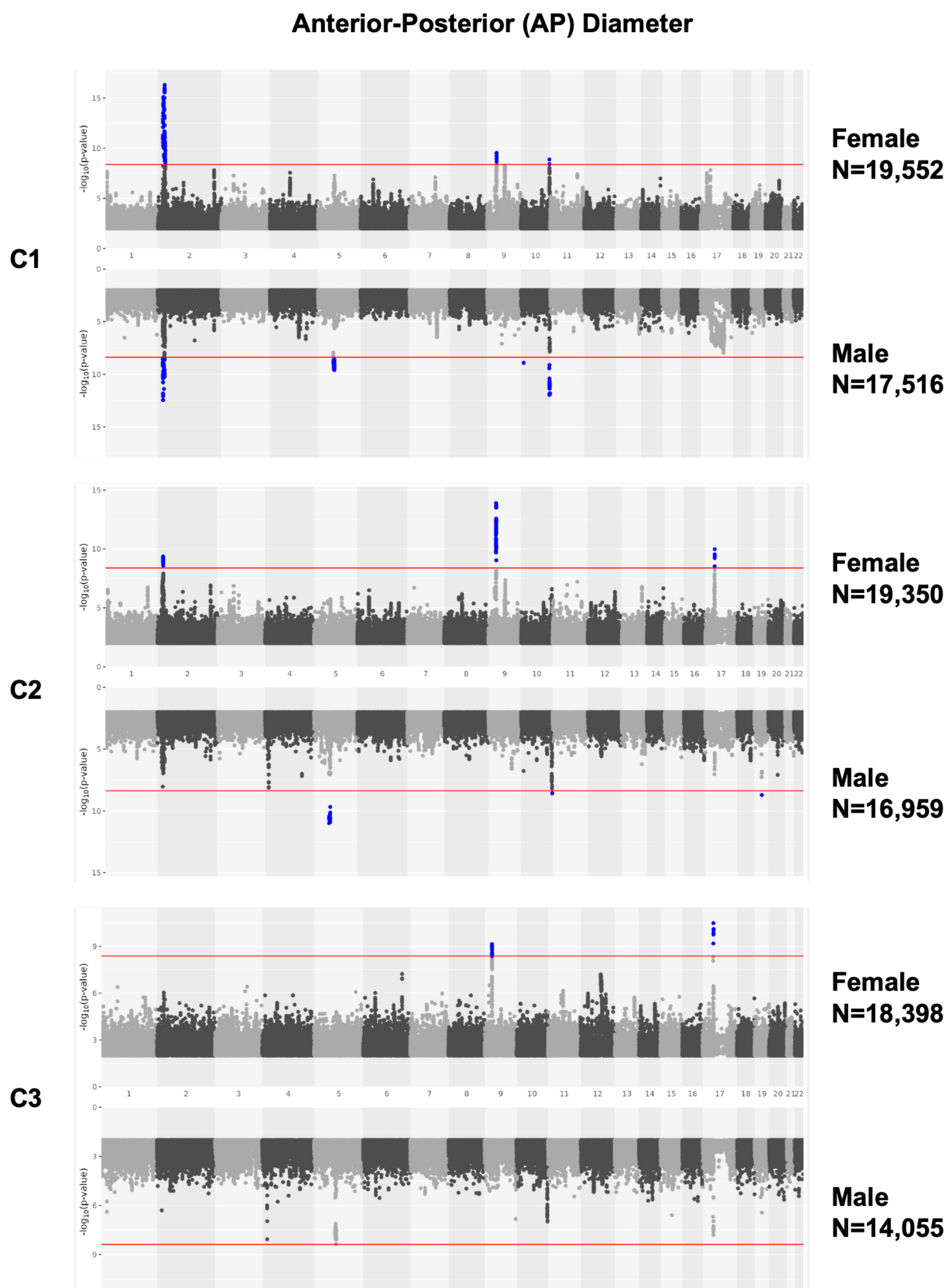

Supplementary Figure 22. Miami plot of sex-stratified GWAS for anterior-posterior diameter. Results for females are shown in the upper panel and for males in the lower panel. The genome-wide significance threshold ($P < 4.2 \times 10^{-9}$) is indicated by the red horizontal line. Genome-wide significant variants are highlighted in blue. SNPs with $-\log_{10}(P) < 2$ are not displayed. Sex-stratified GWAS were performed at each vertebral level, with sample sizes of n = 19,552 females and n = 17,516 males at C1; n = 19,350 females and n = 16,959 males at C2; and n = 18,398 females and n = 14,055 males at C3.

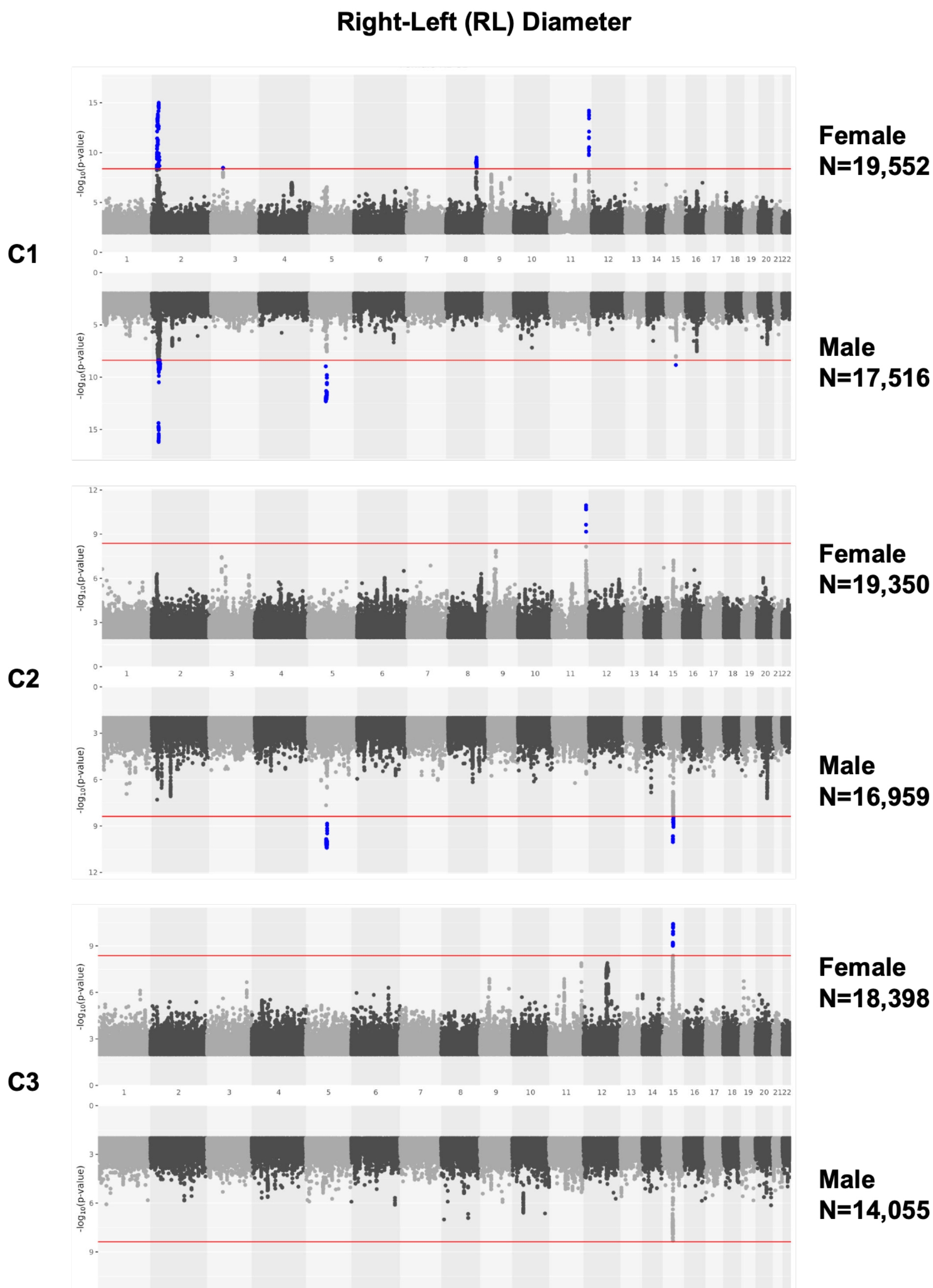

Supplementary Figure 23. Miami plot of sex-stratified GWAS for right-left diameter. Results for females are shown in the upper panel and for males in the lower panel. The genome-wide significance threshold ($P < 4.2 \times 10^{-9}$) is indicated by the red horizontal line. Genome-wide significant variants are highlighted in blue. SNPs with $-\log_{10}(P) < 2$ are not displayed. Sex-stratified GWAS were performed at each vertebral level, with sample sizes of n = 19,552 females and n = 17,516 males at C1; n = 19,350 females and n = 16,959 males at C2; and n = 18,398 females and n = 14,055 males at C3.

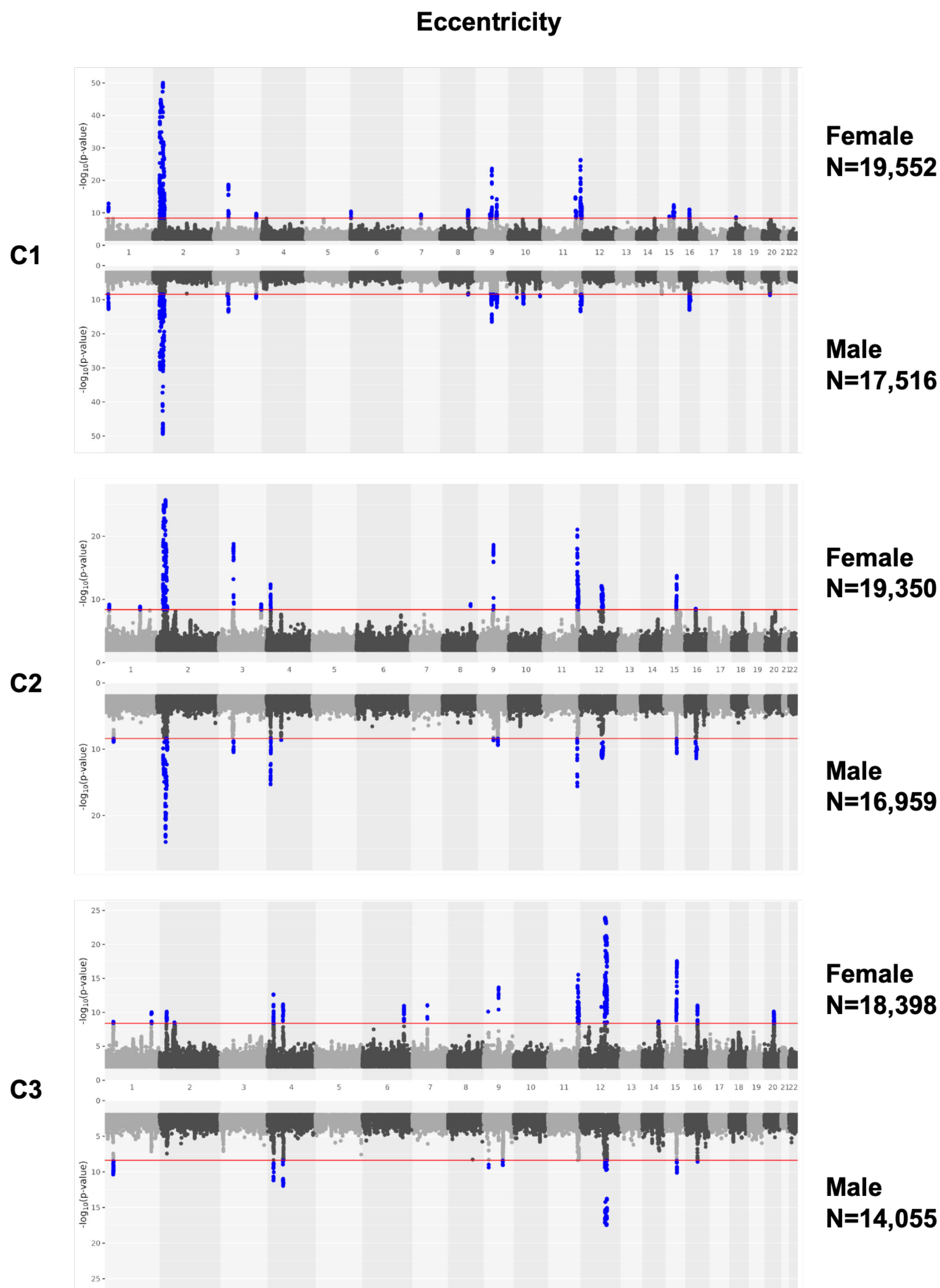

Supplementary Figure 24. Miami plot of sex-stratified GWAS for eccentricity. Results for females are shown in the upper panel and for males in the lower panel. The genome-wide significance threshold ($P < 4.2 \times 10^{-9}$) is indicated by the red horizontal line. Genome-wide significant variants are highlighted in blue. SNPs with $-\log_{10}(P) < 2$ are not displayed. Sex-stratified GWAS were performed at each vertebral level, with sample sizes of n = 19,552 females and n = 17,516 males at C1; n = 19,350 females and n = 16,959 males at C2; and n = 18,398 females and n = 14,055 males at C3.

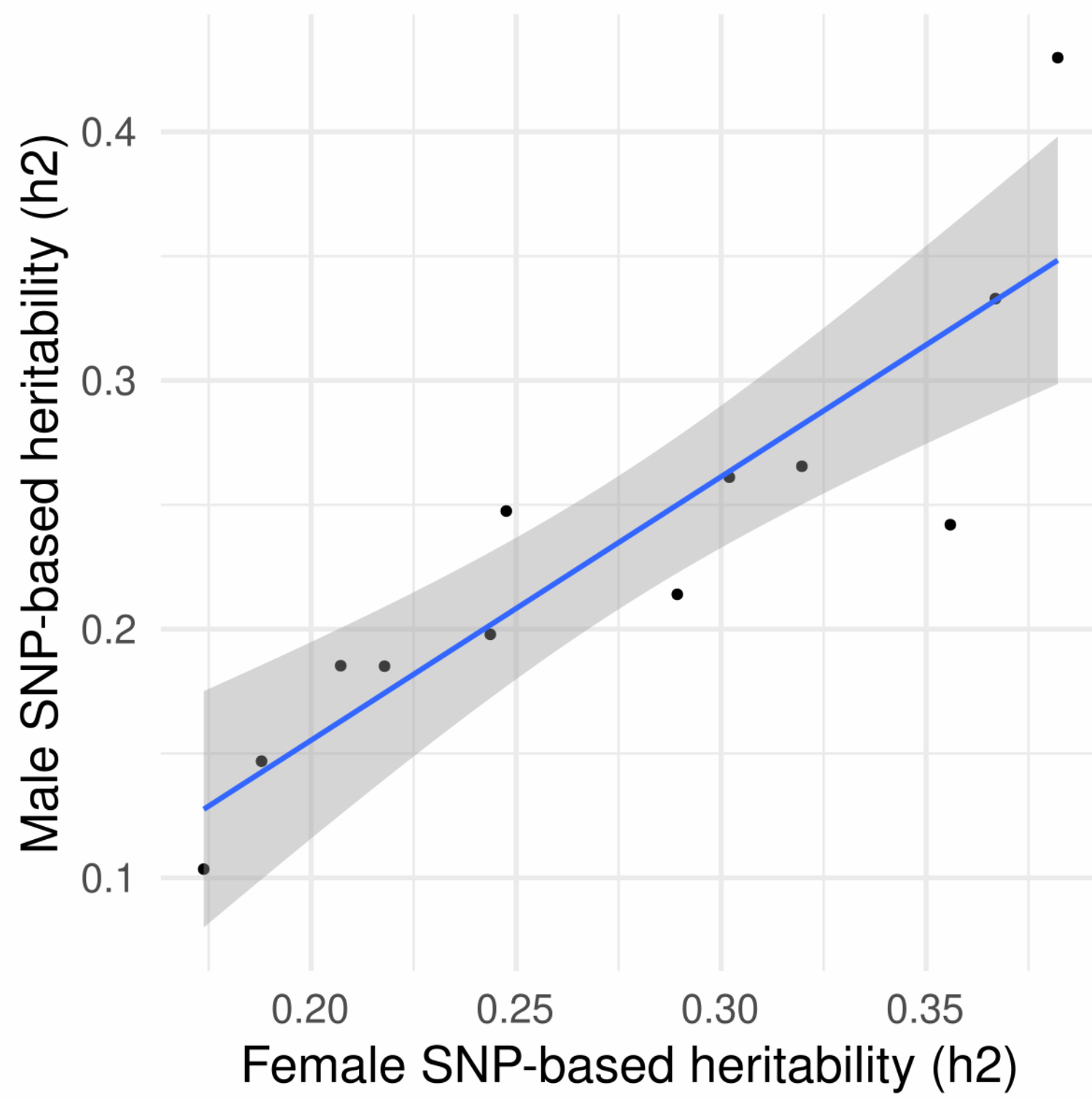

Supplementary Figure 25. Correlation of sex-specific SNP-based heritability estimates. The scatter plot shows the correlation between male- and female-specific SNP-based heritability estimates across 12 spinal cord shape metrics. Each point represents one phenotype. The source data for this figure is in Supplementary Data 7.

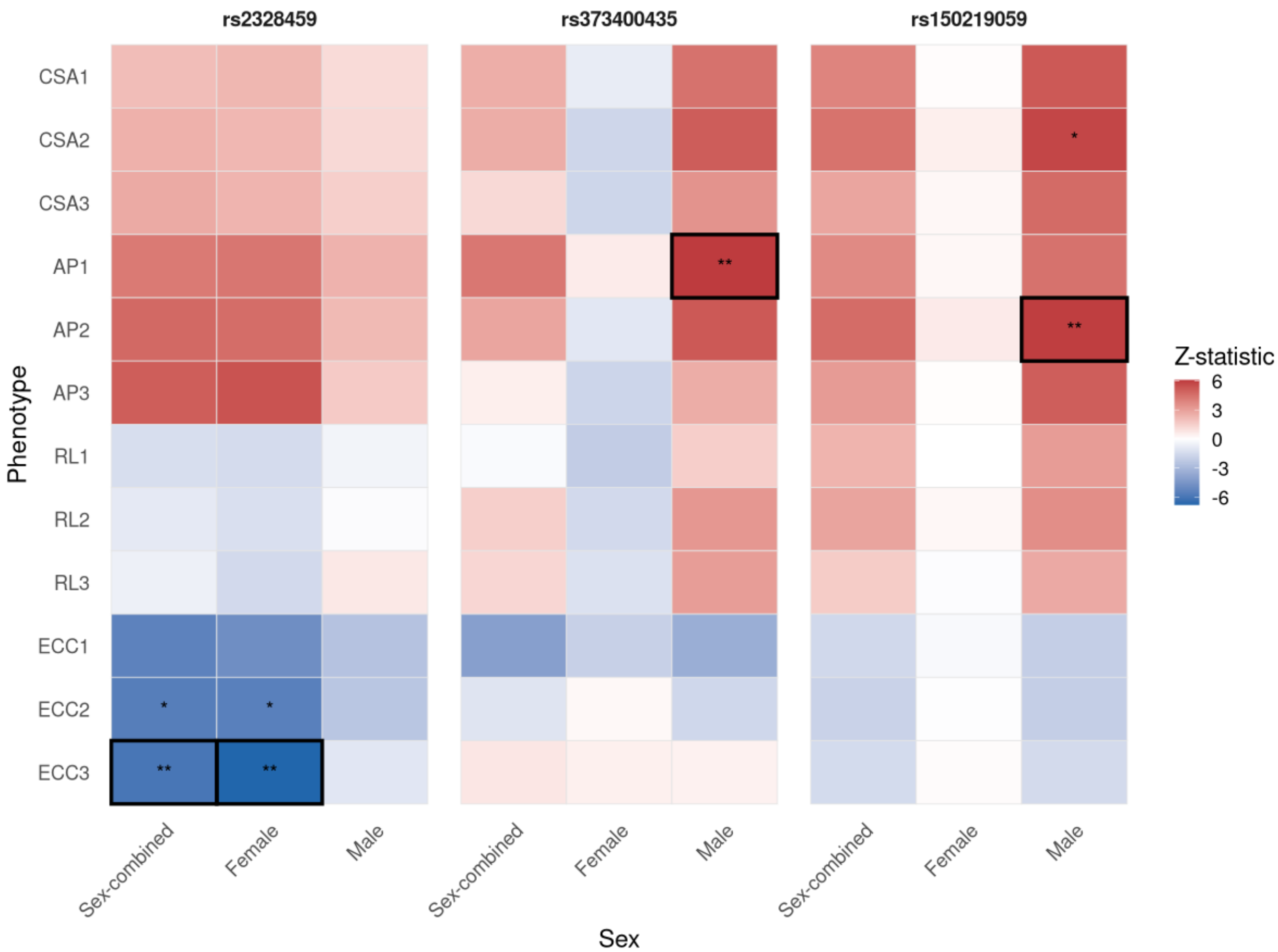

Supplementary Figure 26. Heatmap showing three SNPs (rs2328459, rs373400435, rs150219059) with significant SNP-by-sex interaction effects. Colour represents the z-statistic (beta/standard error) from sex-combined and sex-specific GWAS. A single asterisk (*) indicates $P < 5 \times 10^{-8}$, and a double asterisk (**) indicates $P < 4.2 \times 10^{-9}$. The source data for this figure is in Supplementary Data 10.

Section 8: Association in the replication GWAS

For replication, we performed GWAS in a European-ancestry cohort within the UK Biobank using additional individuals from the imaging release v20.5 (2025), applying the same analytical framework as in the discovery analysis. The replication population included 27,073 individuals at the C1 spinal level, 26,234 at C2, and 22,450 at C3.

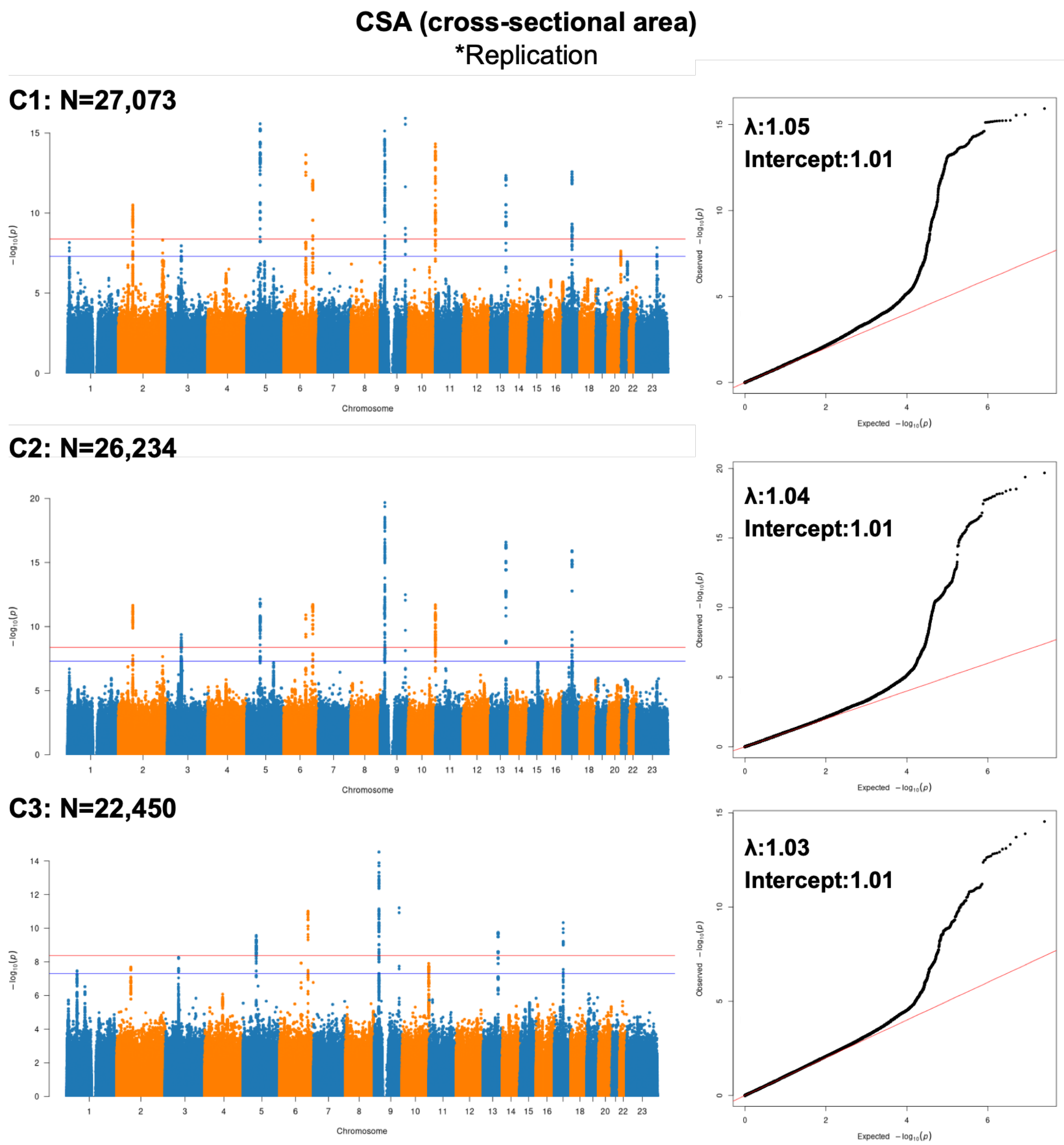

Supplementary Figure 27. Manhattan and quantile–quantile (Q–Q) plots for cross-sectional area at the C1, C2, and C3 vertebral levels in the European replication sample. In Manhattan plots, each point represents a single genetic variant according to its genomic position (x-axis) and $-\log_{10}P$ (two-sided P values). The blue horizontal line indicates the conventional genome-wide significance threshold ($P < 5 \times 10^{-8}$), while the red line marks the Bonferroni-corrected threshold used in this study ($P < 4.2 \times 10^{-9}$), accounting for 12 phenotypes. In the Q–Q plots, the red diagonal line represents the expected null distribution. Genomic inflation factors (λ) and LD Score regression intercepts are labelled within each plot. This analysis included n = 27,073 (C1), n = 26,234 (C2), and n = 22,450 (C3) independent UK Biobank participants.

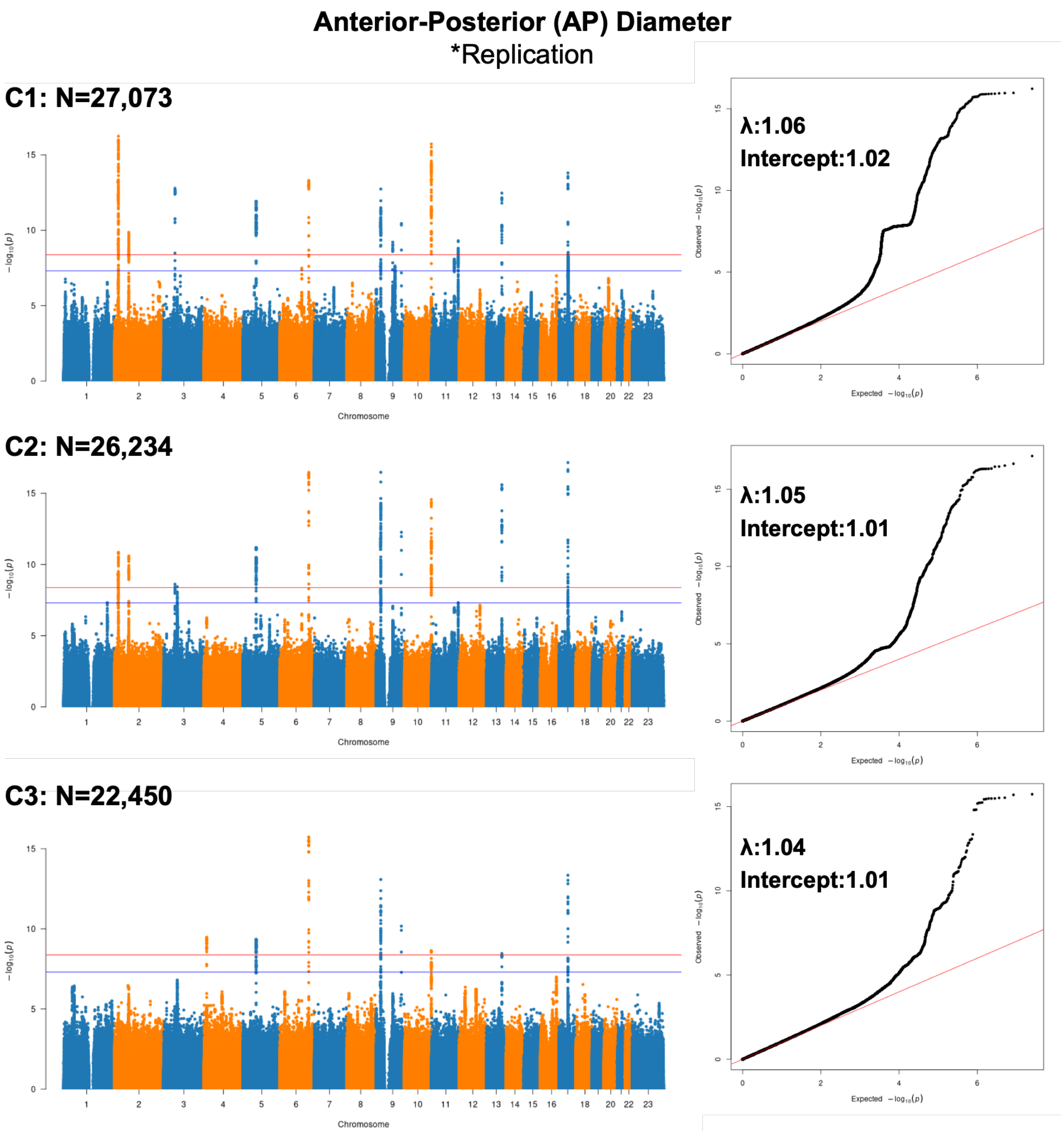

Supplementary Figure 28. Manhattan and quantile–quantile (Q–Q) plots for anterior-posterior diameter at the C1, C2, and C3 vertebral levels in the European replication sample. In Manhattan plots, each point represents a single genetic variant according to its genomic position (x-axis) and $-\log_{10}P$ (two-sided P values). The blue horizontal line indicates the conventional genome-wide significance threshold ($P < 5 \times 10^{-8}$), while the red line marks the Bonferroni-corrected threshold used in this study ($P < 4.2 \times 10^{-9}$), accounting for 12 phenotypes. In the Q–Q plots, the red diagonal line represents the expected null distribution. Genomic inflation factors (λ) and LD Score regression intercepts are labelled within each plot. This analysis included n = 27,073 (C1), n = 26,234 (C2), and n = 22,450 (C3) independent UK Biobank participants.

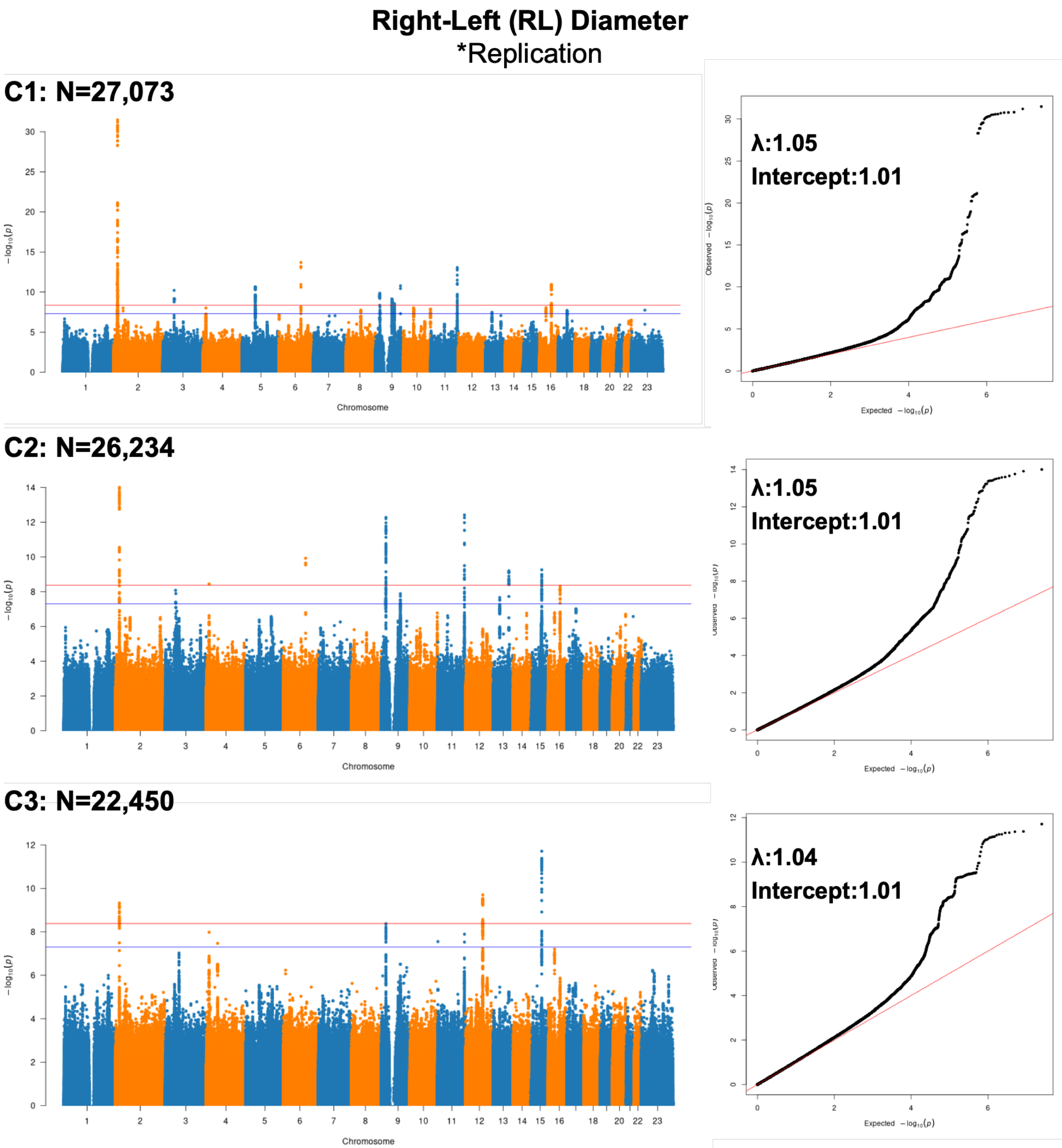

Supplementary Figure 29. Manhattan and quantile–quantile (Q–Q) plots for right-left diameter at the C1, C2, and C3 vertebral levels in the European replication sample. In Manhattan plots, each point represents a single genetic variant according to its genomic position (x-axis) and $-\log_{10}P$ (two-sided P values). The blue horizontal line indicates the conventional genome-wide significance threshold ($P < 5 \times 10^{-8}$), while the red line marks the Bonferroni-corrected threshold used in this study ($P < 4.2 \times 10^{-9}$), accounting for 12 phenotypes. In the Q–Q plots, the red diagonal line represents the expected null distribution. Genomic inflation factors (λ) and LD Score regression intercepts are labelled within each plot. This analysis included n = 27,073 (C1), n = 26,234 (C2), and n = 22,450 (C3) independent UK Biobank participants.

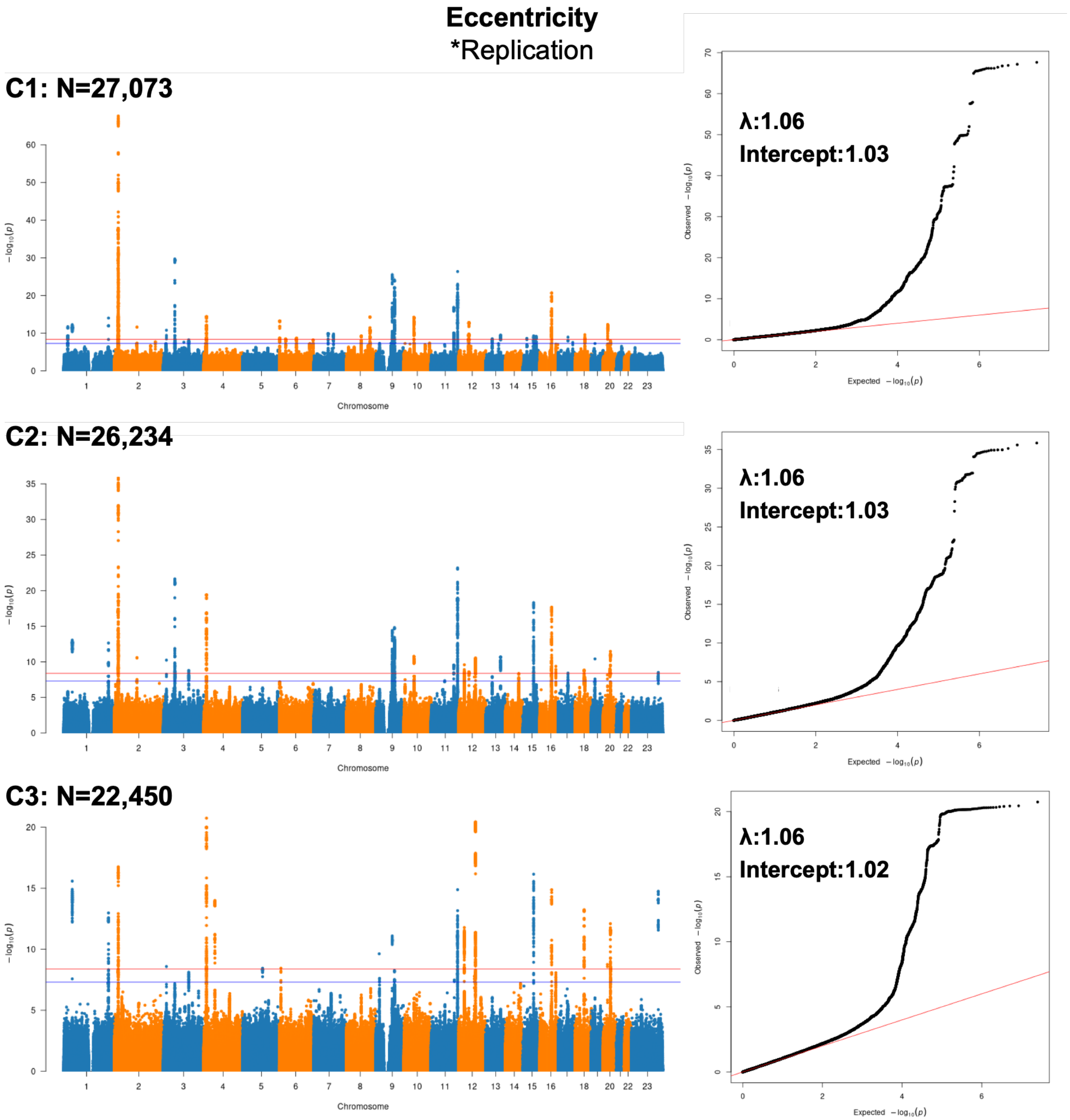

Supplementary Figure 30. Manhattan and quantile–quantile (Q–Q) plots for eccentricity at the C1, C2, and C3 vertebral levels in the European replication sample, without height as a covariate. In Manhattan plots, each point represents a single genetic variant according to its genomic position (x-axis) and $-\log_{10}P$ (two-sided P values). The blue horizontal line indicates the conventional genome-wide significance threshold ($P < 5 \times 10^{-8}$), while the red line marks the Bonferroni-corrected threshold used in this study ($P < 4.2 \times 10^{-9}$), accounting for 12 phenotypes. In the Q–Q plots, the red diagonal line represents the expected null distribution. Genomic inflation factors (λ) and LD Score regression intercepts are labelled within each plot. This analysis included n = 27,073 (C1), n = 26,234 (C2), and n = 22,450 (C3) independent UK Biobank participants.

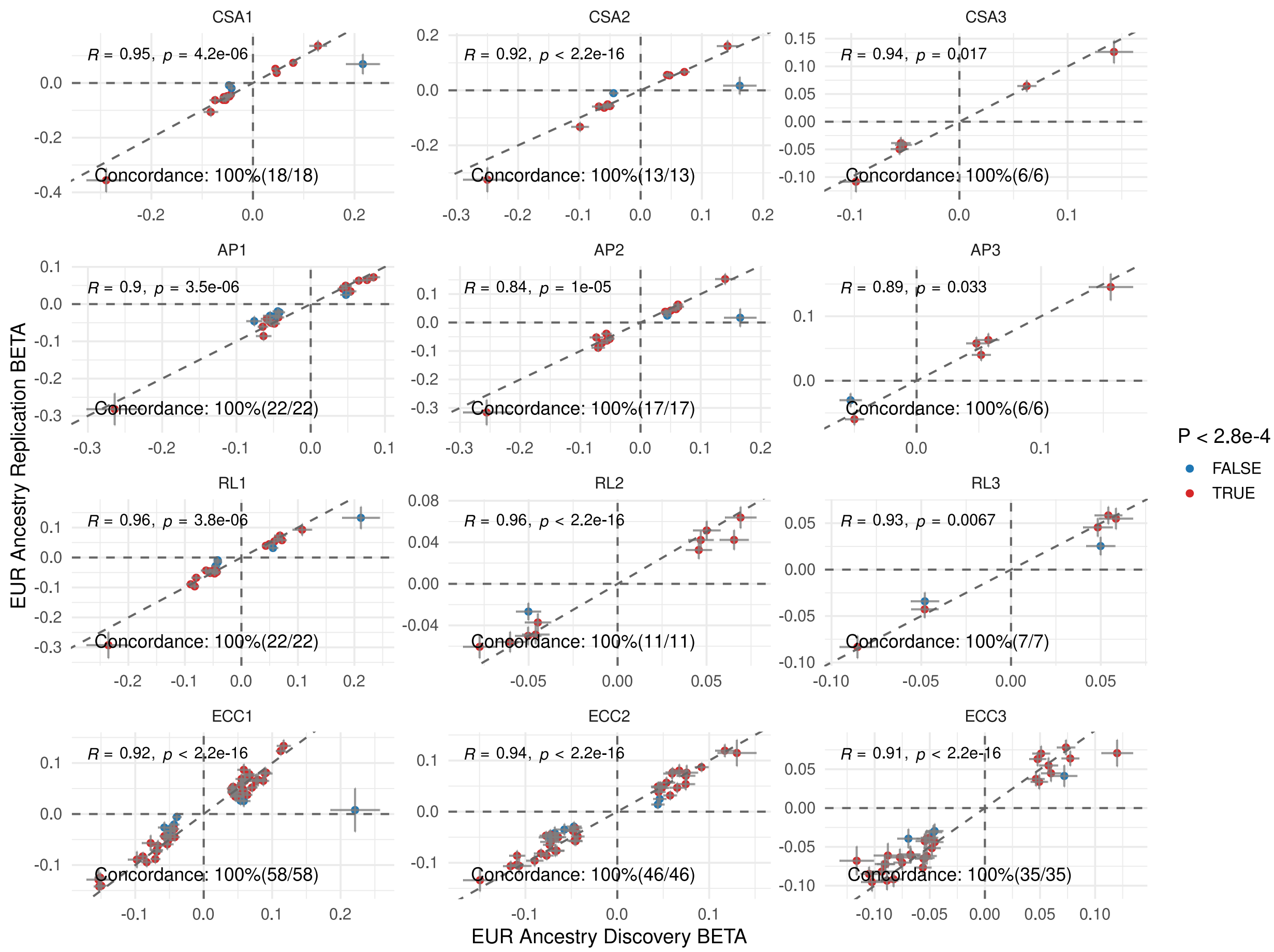

Supplementary Figure 31: Comparison of effect sizes between the European (EUR) discovery and EUR replication cohorts. The plot includes 179 genome-wide significant independent SNPs identified in the EUR GWAS. Spearman's rank correlation coefficient and directional concordance are indicated. Each point represents the per-allele effect size, with error bars showing the 95% confidence interval. Across the 12 traits, 71.4% - 90.0% loci reached Bonferroni-corrected significance ($P < 0.00028$, correcting for 179 tests). The source data for this figure is in Supplementary Data 13.

Section 9: Association in the non-European ancestries

To evaluate the transferability of association signals across ancestries, we assessed whether the independent significant SNPs identified in the European-ancestry GWAS of spinal cord shape metrics were also associated in non-European individuals from the UK Biobank. These analyses were conducted in three cohorts: Central/South Asian (n = 893-807), AFR (n =463-423), and EAS (EAS; n = 389-358) participants across the C1 to C3 vertebral levels. Sample sizes slightly varied across levels due to progressively lower image quality from C1 to C3.

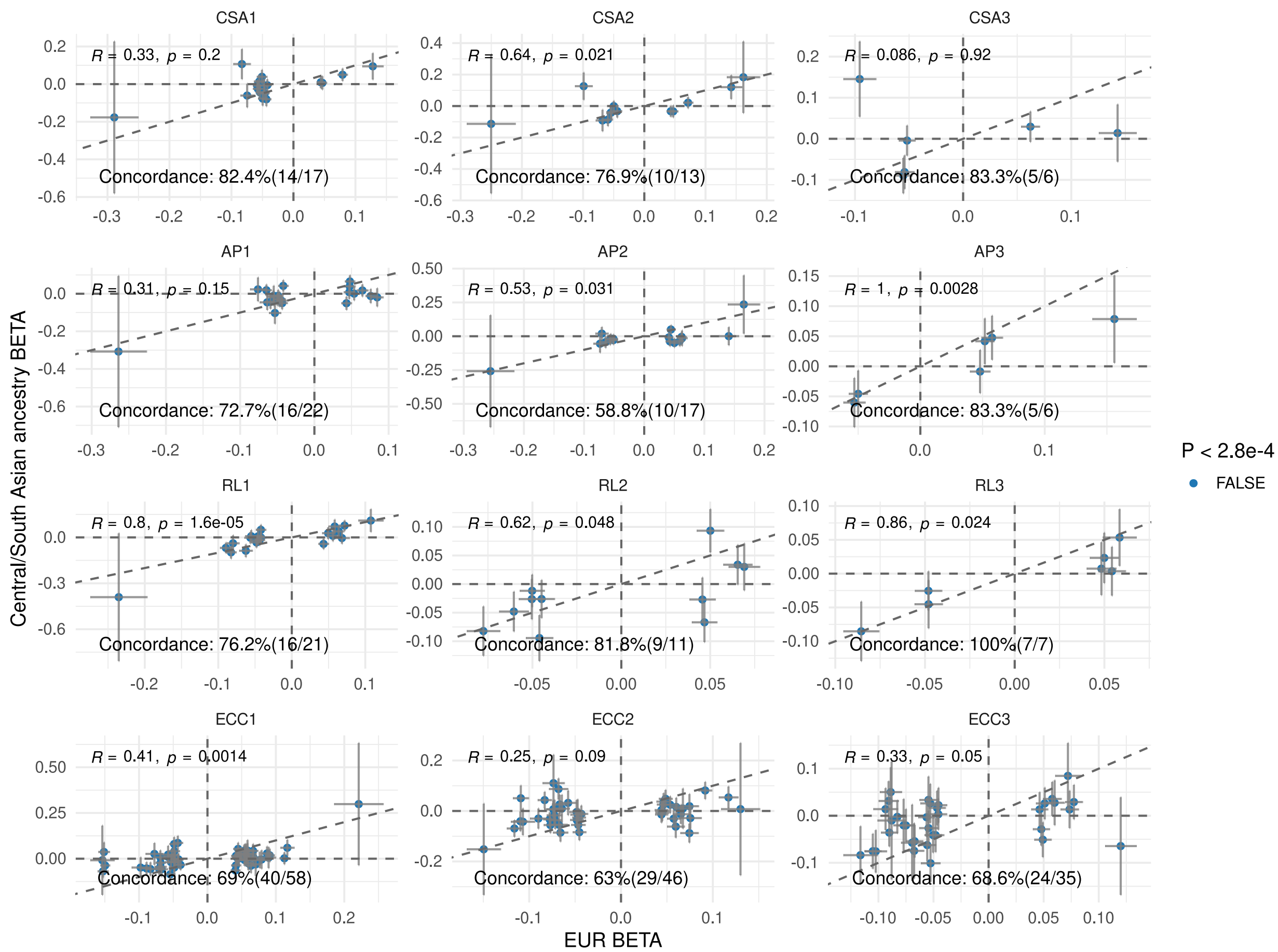

Supplementary Figure 32: Comparison of effect sizes between the European (EUR) and Central/South Asian cohorts. The plot includes 178 genome-wide significant independent SNPs (one SNP was unavailable in the CSA cohort) identified in the EUR GWAS. Spearman's rank correlation coefficient and directional concordance are indicated. A two-sided sign test assessed whether SNP effect directions were concordant beyond chance. Each point represents the per-allele effect size, with error bars showing the 95% confidence interval. No variants reached Bonferroni-corrected significance (P < 0.00028, correcting for 179 tests). This analysis included European (n = 37, 068; 36,309; 32,453) and Central/South Asian (n = 893, 878, 807) participants.

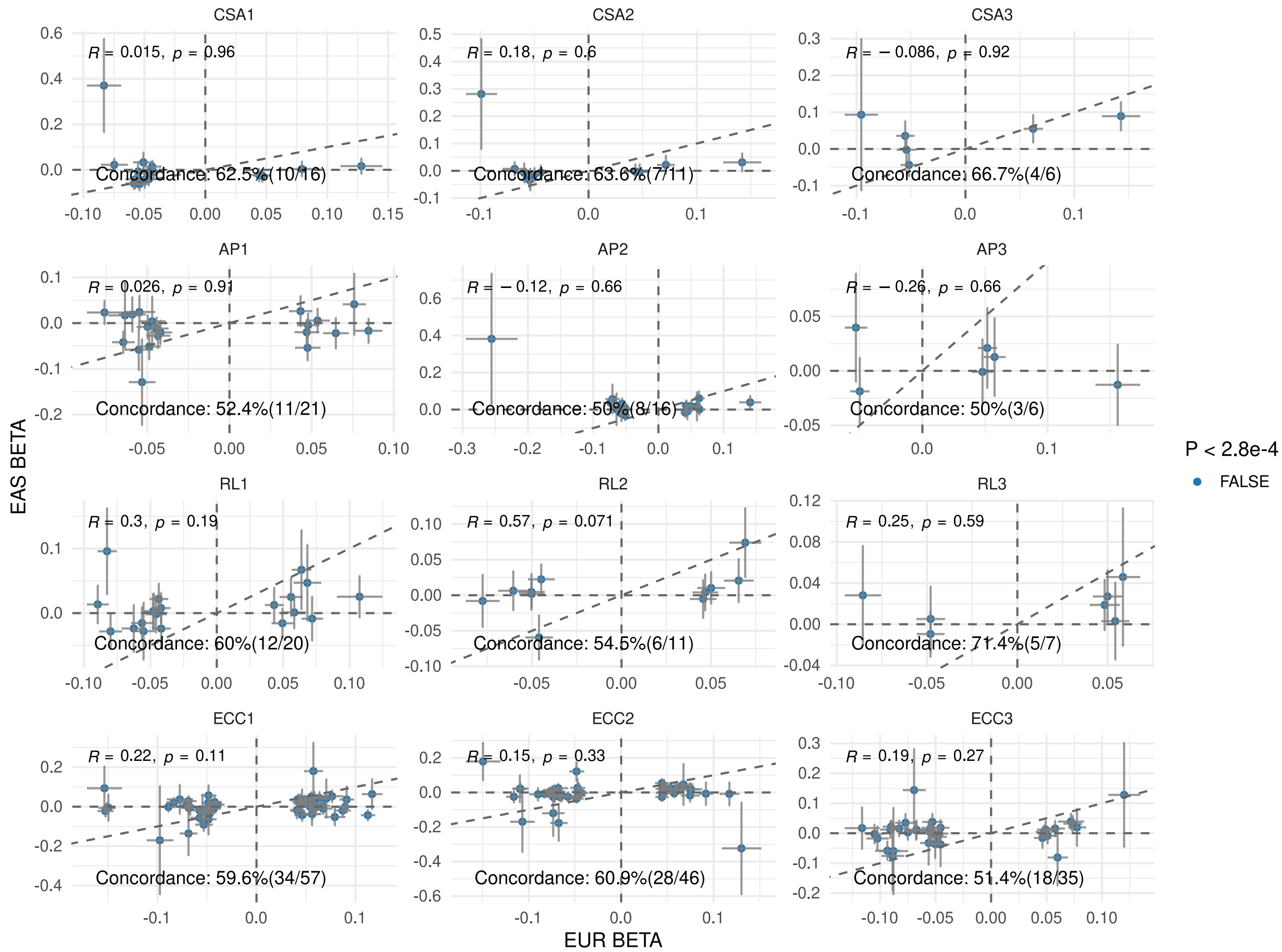

Supplementary Figure 33: Comparison of effect sizes between the European (EUR) and East Asian (EAS) cohorts. The plot includes 175 genome-wide significant independent SNPs (fourSNPs were unavailable in the EAS cohort) identified in the EUR GWAS. Spearman's rank correlation coefficient and directional concordance are indicated. A two-sided sign test assessed whether SNP effect directions were concordant beyond chance. Each point represents the per-allele effect size, with error bars showing the 95% confidence interval. No variants reached Bonferroni-corrected significance ($P < 0.00028$, correcting for 179 tests). This analysis included European (n = 37, 068; 36,309; 32,453) and EAS (EAS; n = 389, 380, 358) participants.

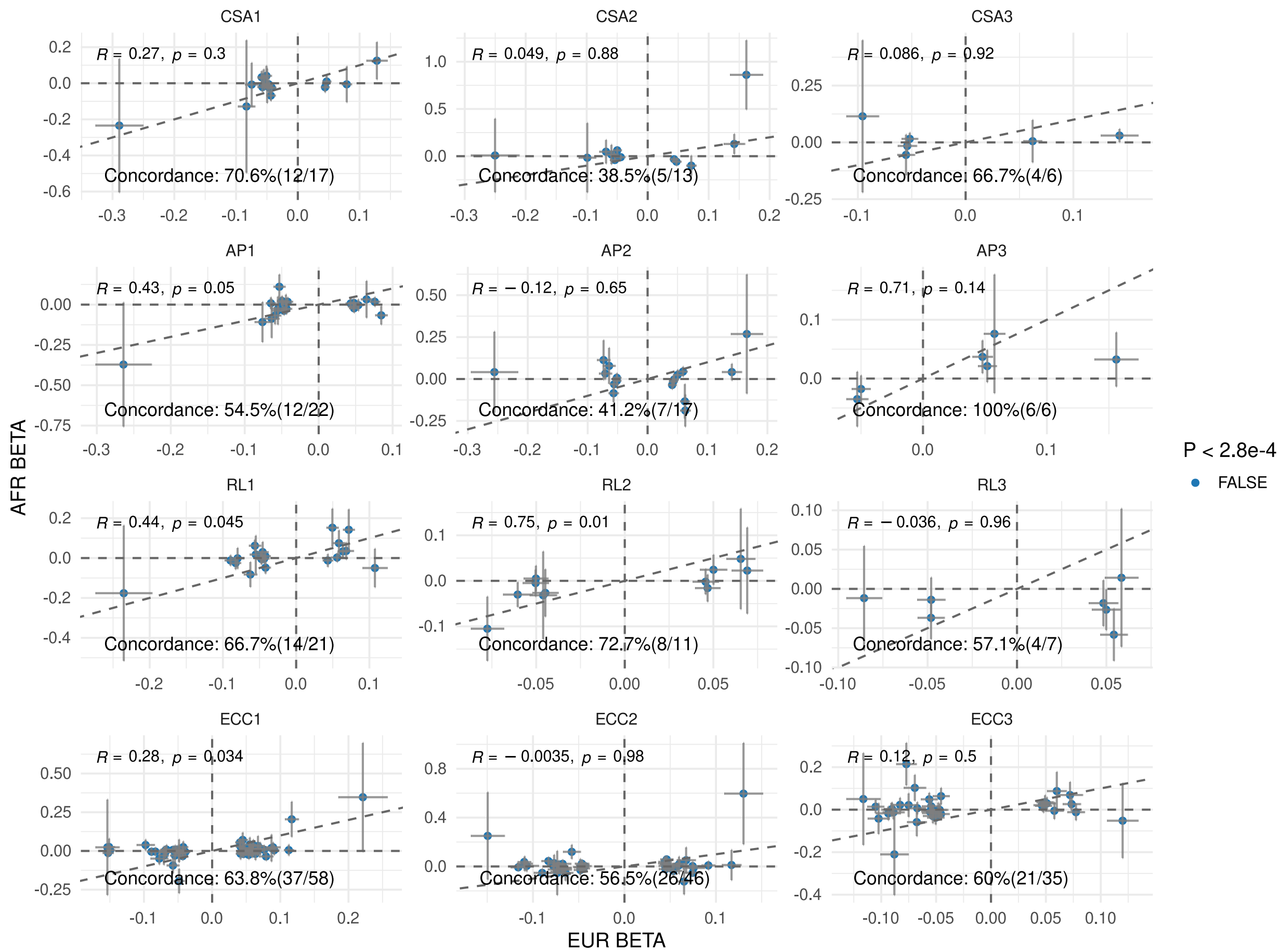

Supplementary Figure 34: Comparison of effect sizes between the European (EUR) and African (AFR) cohorts. The plot includes 178 genome-wide significant independent SNPs (one SNP was unavailable in the AFR cohort) identified in the EUR GWAS. Spearman's rank correlation coefficient and directional concordance are indicated. A two-sided sign test assessed whether SNP effect directions were concordant beyond chance. Each point represents the per-allele effect size, with error bars showing the 95% confidence interval. No variants reached Bonferroni-corrected significance (P < 0.00028, correcting for 179 tests). This analysis included European (n = 37, 068; 36,309; 32,453) and AFR (n =463, 450, 423) participants.

Section 10: Gene-based findings

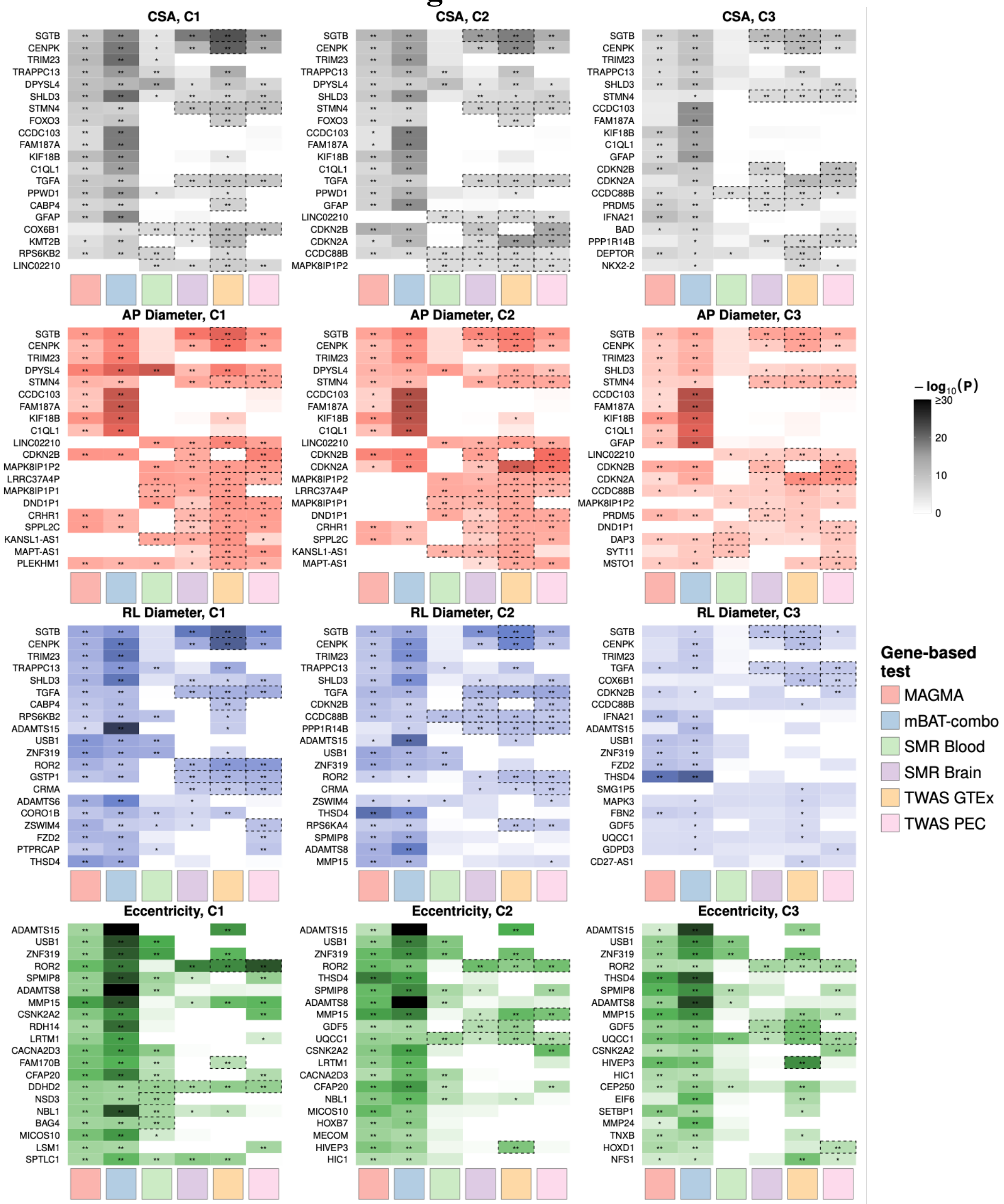

Supplementary Figure 35: Top 20 prioritised genes for each cervical spinal cord morphology phenotype. Gene-level association across six gene-based tests for the twelve spinal cord phenotypes (four morphological measures at cervical levels C1–C3; N = 37,068). Each panel shows the 20 highest-ranked genes for that phenotype, ordered by the mean $-\log_{10}(P)$ across the six tests. Asterisks denote significance after correction within each test and phenotype: ** Bonferroni-adjusted $P < 0.05$, * Benjamini–Hochberg FDR < 0.05. Dashed borders mark associations with additional functional support — TWAS associations with a colocalisation posterior probability PP.H4 > 0.80, or SMR associations with HEIDI P > 0.05 — in both cases together with Bonferroni significance; this annotation does not apply to MAGMA or mBAT-combo, which do not yield these statistics. Cells without fill were either not evaluated by that test or showed no association.

Section 11: Genetic and phenotypic correlations between spinal cord phenotypes and other brain structures

To assess phenotypic correlations between spinal cord and brain structures, we selected a range of brain regions available in the UK Biobank imaging dataset. Specifically, we included brainstem subregion volumes (Freesurfer subsegmentation, Category 191), subcortical structure volumes (Freesurfer ASEG, Category 190), and cortical region volumes defined by the Brodmann Area Maps (Freesurfer BA exvivo, Category 195). These regions were chosen to capture structural variation across multiple levels of the central nervous system. Genetic correlations were calculated from summary statistics from previous GWAS studies listed in Supplementary Data 42.

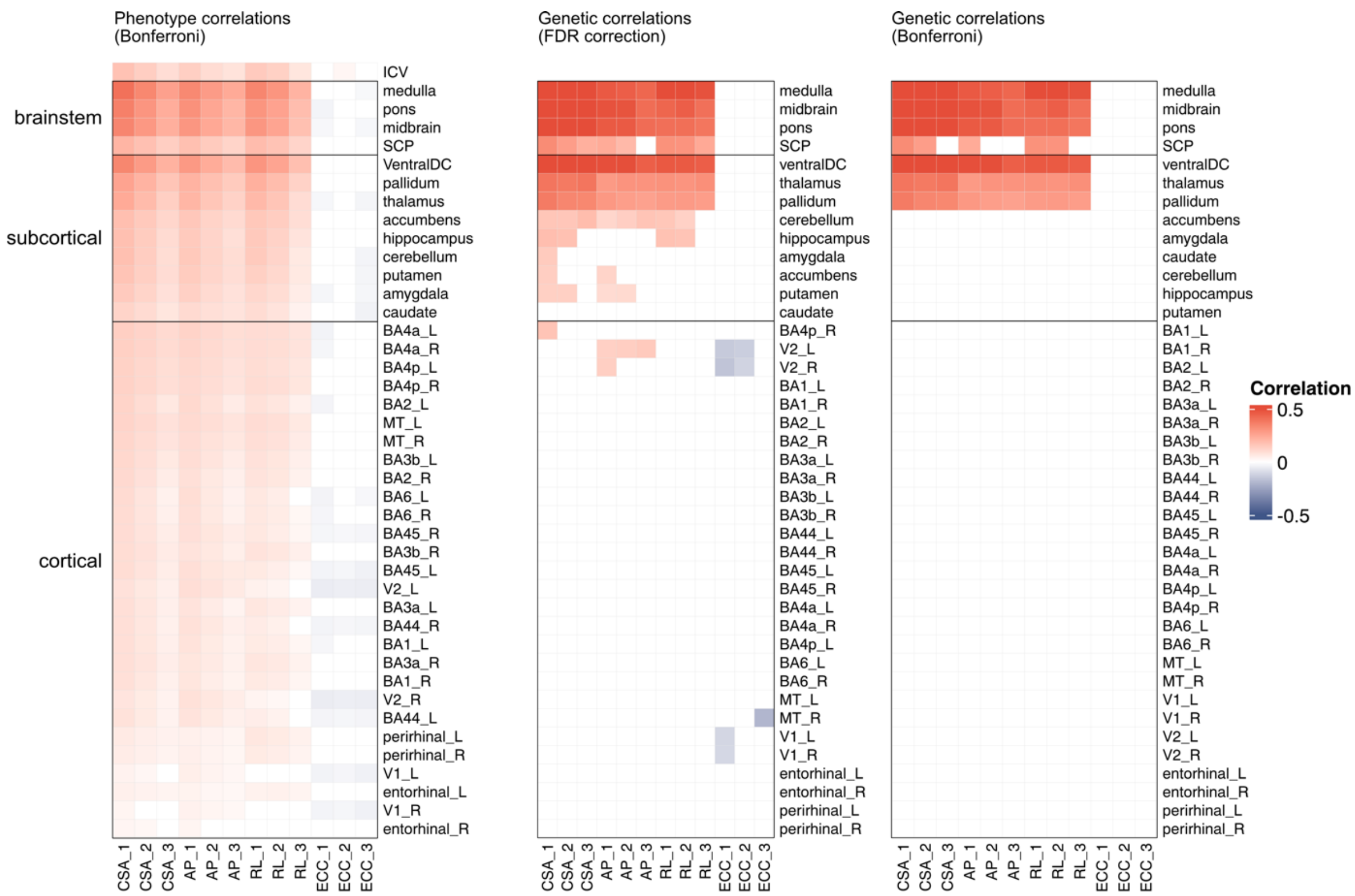

Supplementary Figure 36: Heatmaps showing correlations between spinal cord shape metrics and brain volumes. (a) Phenotypic correlations between spinal cord cross-sectional area (CSA), anterior-posterior (AP) diameter, right–left (RL) diameter, eccentricity, and brainstem, subcortical, and cortical brain volumes, including the intracranial volume (ICV). ICV was only included in the phenotypic correlations as it was used as a covariate in the GWAS. Phenotypic correlations (two-sided, significant after Bonferroni correction for multiple comparisons, $P < 0.05/504$). (b) Genetic correlations (FDR-corrected significance threshold < 0.05). (c) Genetic correlations (significant after Bonferroni correction for multiple comparisons, $P < 0.05/492$). Source data for this figure is in Supplementary Data 32 and 33.

Section 12: Polygenic risk associations with disease outcomes

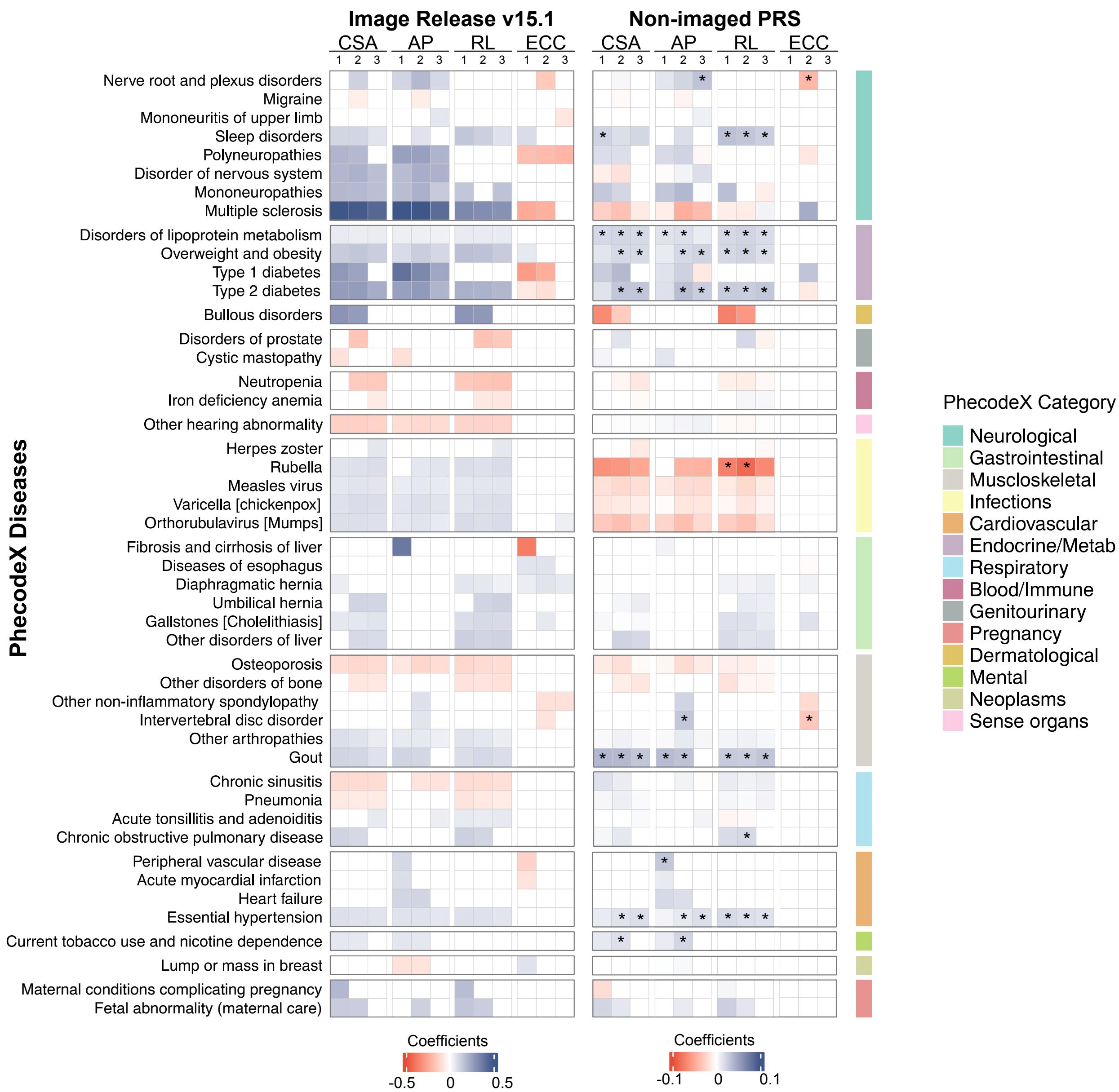

Supplementary Figure 37: Comparison of phenotypic associations and spinal cord polygenic risk scores (PRS) associations with PheCodeX diseases. The left column shows 47 diseases with significant associations (FDR adjusted P-values < 0.05) with at least two spinal cord phenotypes (also presented in Fig. 6a). The right column shows associations between spinal cord PRS in non-imaging samples and these same 47 diseases. Only diseases significant in the left column are displayed. Asterisks (*) indicate associations significant after FDR correction. Colours represent regression coefficients. Note viral infections have reporting bias among non-imaged participants. . Source data for this figure is in Supplementary Data 34, 36.

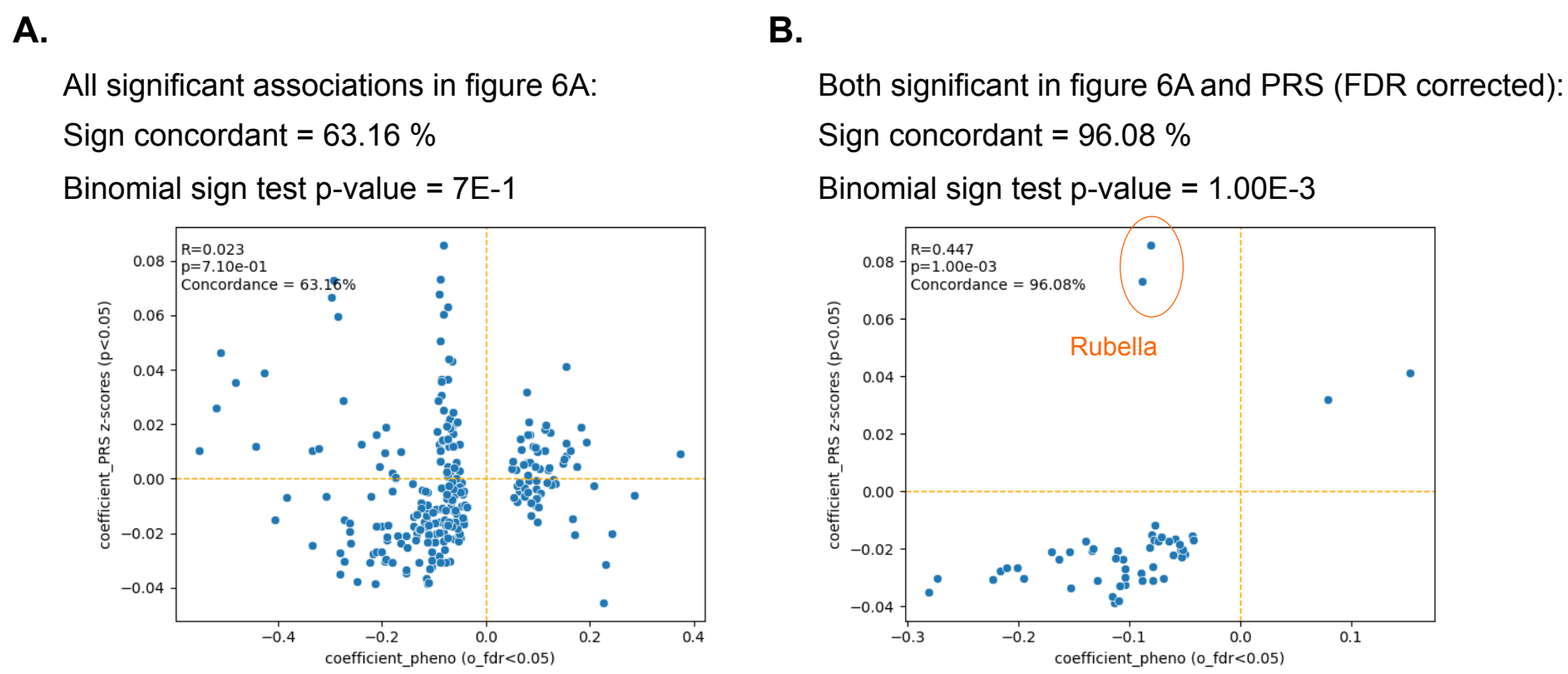

Supplementary Figure 38: Concordance of phenotypic and spinal cord PRS associations with disease. Scatter plots compare regression coefficients from phenotypic associations (x-axis) and PRS–disease associations (y-axis). (A) All FDR-corrected significant phenotypic associations are shown together with PRS associations regardless of significance (sign concordance = 63.16% of tests; Pearson's R = 0.023). (B) FDR-corrected significant phenotypic associations and FDR-corrected significant PRS associations are shown (sign concordance = 96.08%). Discordant cases are circled and correspond to rubella. Source data for this figure is in Supplementary Data 34, 36.

ID_067 Rubella

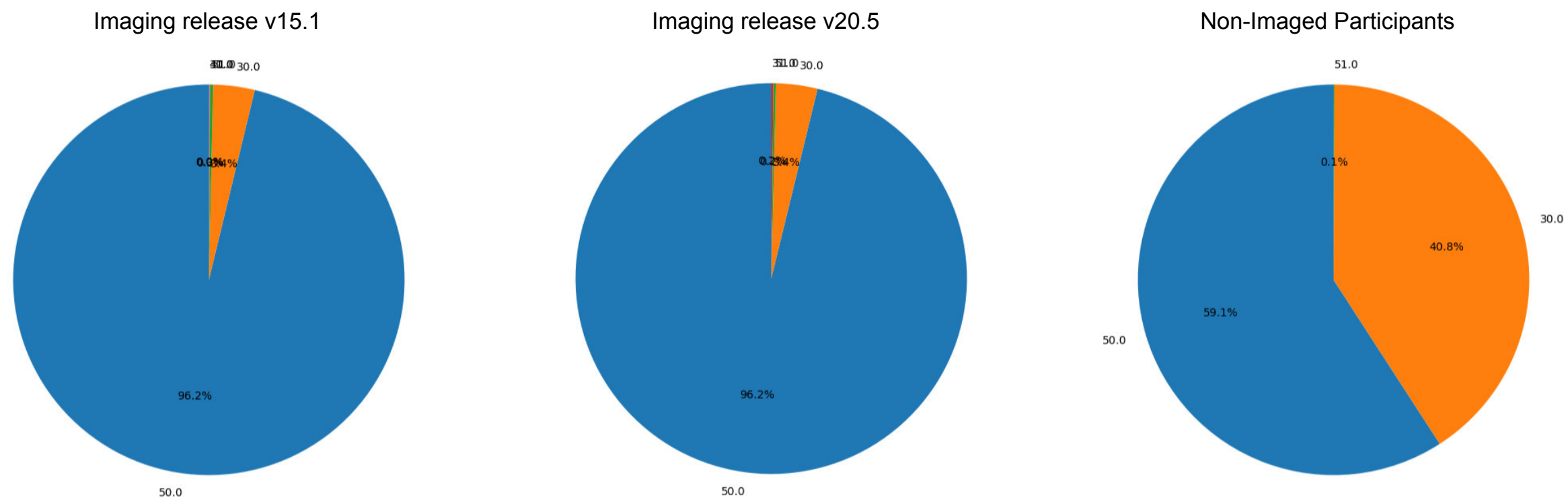


```
Self-report only: 2168
Primary care only: 77
Self-report and other sources: 6
Primary care and other sources: 1
Hospital only: 1
```

```
Self-report only: 1718
Primary care only: 61
Self-report and other sources: 4
Primary care and other sources: 3
```

```
Self-report only: 936
Primary care only: 646
Self-report and other sources: 1
```

Supplementary Figure 39: Reporting sources for rubella viral infection. Pie charts compare the distribution of health record sources between imaged and non-imaged participants. Blue indicates "self-report only" and orange indicates "primary care only". The higher proportion of self-reported cases in the imaged cohort reflects a reporting bias, where a significant number of records were added during subsequent imaging visits. Source data for this figure is in Supplementary Data 35.

Section 12: Mendelian randomization

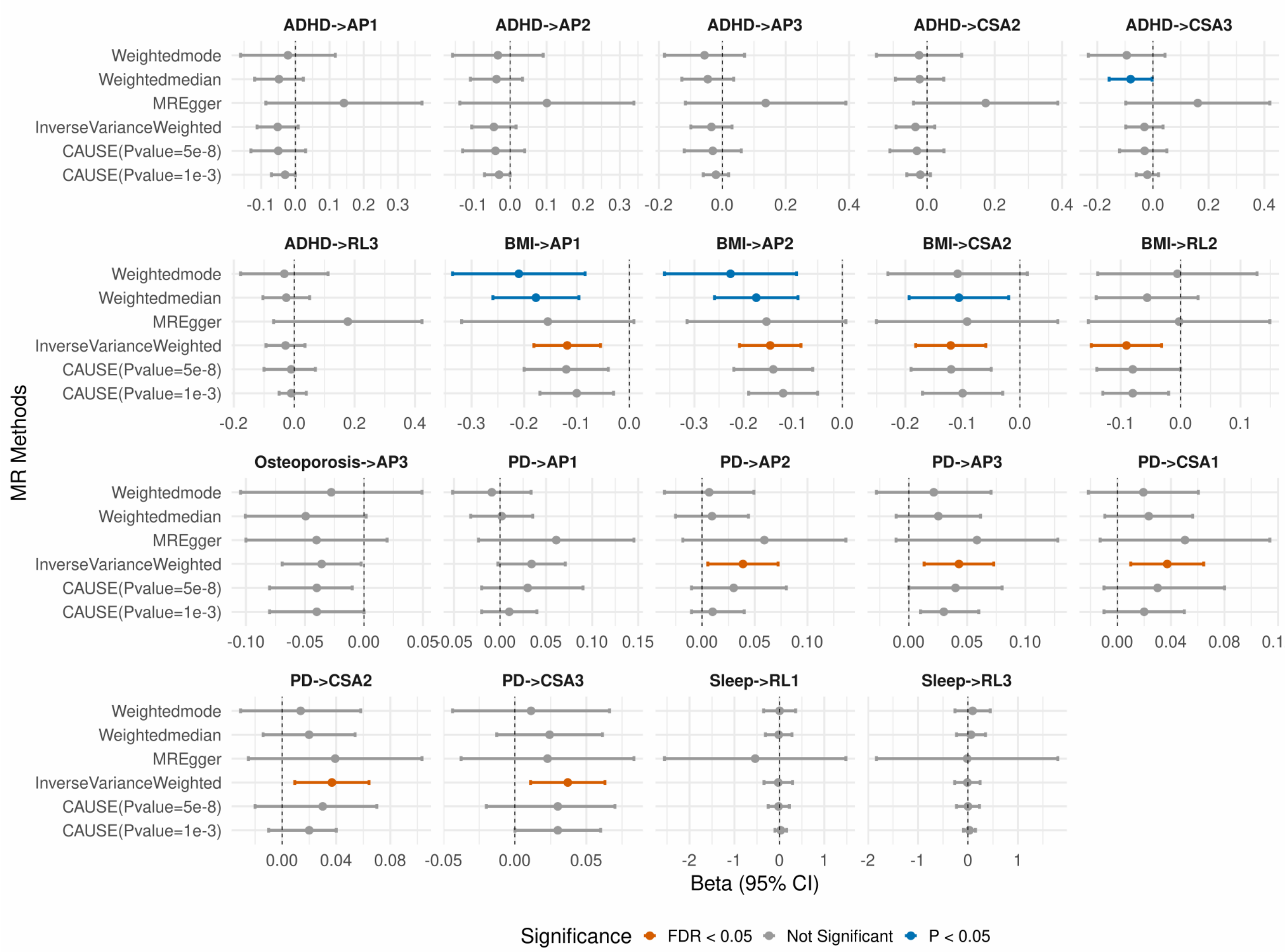

Supplementary Figure 40: Forward Mendelian randomization (MR) analysis assessing the causal effect of spinal cord phenotypes on various traits. The x-axis represents effect size estimates (Beta) with 95% confidence intervals (CI); the y-axis represents MR methods. Colored by statistical significance: nominal (P < 0.05) - orange, not significant - grey, or FDR < 0.05 - blue, based on the inverse-variance weighted method. Source data for this figure is in Supplementary Data 38, 39.

Section 14: Spinal cord image quality control

14.1 Image QC overview

A key QC challenge is the inconsistent image quality across the C1 to C3 regions in the same image, primarily due to signal drop-off near the edges of the scan. To address this, we implemented a novel and rigorous QC procedure. First, T1-weighted images with poor overall quality and severe artifacts (flagged as unusable by the standard UK Biobank QC protocol[3]), were pre-excluded. Second, we applied vertebral level–specific manual QC to remove spinal cord labels with visibly poor quality. All vertebral labels were manually inspected as a conservative approach, given that these phenotypes had not been previously derived at scale. Third, we excluded additional data points showing large residuals between processing steps. This step accounts for instances where vertebral labelling was reliable, but errors still occurred during phenotype extraction from the segmentation mask.

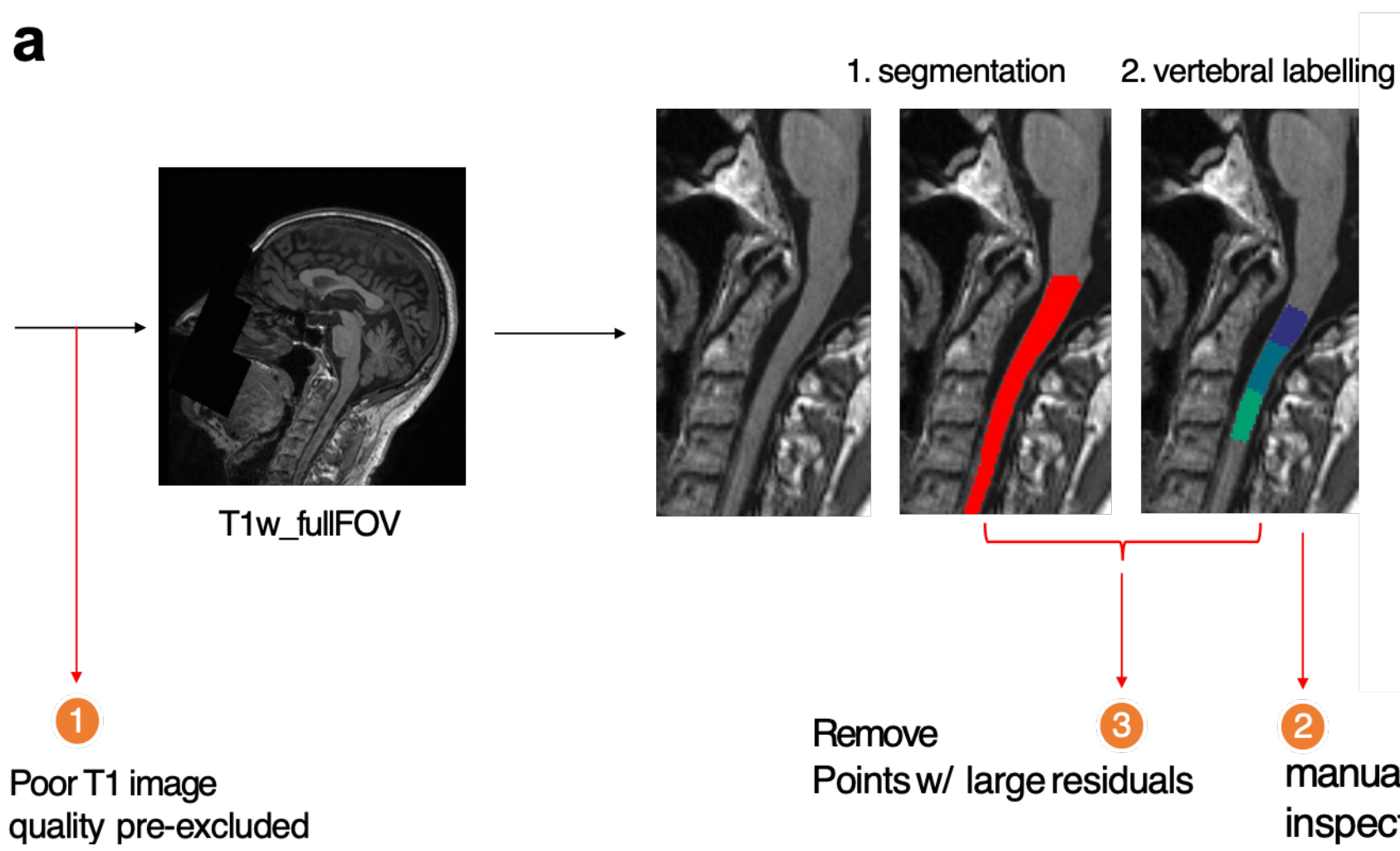

b

After QC step #	C1	C2	C3
1		46366	
2	45734	45044	40304
3	45684	44737	40024
%Excluded	1.5%	3.5%	13.7%

Supplementary Figure 41: summary of spinal cord image QC procedure. (a) first images with poor overall quality were pre-excluded from processing the spinal cord pipeline. Then vertebral labels were manually inspected using the manual QC procedure (figure below). Lastly, points with large residuals between processing steps were removed. (b) a summary of sample sizes after each QC step. Source data for (b) is in source data file. Reproduced by kind permission of UK Biobank ©.

14.2 Manual QC on vertebral labels

The manual QC process followed a systematic three-step approach. First, we generated QC snapshots using SCT's sct_qc command and screened them using a custom-built toolbox that allowed rapid identification of poor-quality or mislabelled vertebral regions. Multiple regions could be marked as QC failures. Representative examples of failed QC cases are shown in Supplementary figure 43. These failed QC cases are due to inaccurate vertebral level labelling due to poor image quality or mislabels. Second, any uncertain cases were reviewed in 3D, since the standard QC images only show mid-sagittal slices. Finally, we visualized failed cases in the context of the full phenotype distribution among every 1000 samples. For any unlabelled outliers, we conducted further manual inspection to sure these points are not produced by image artifacts.

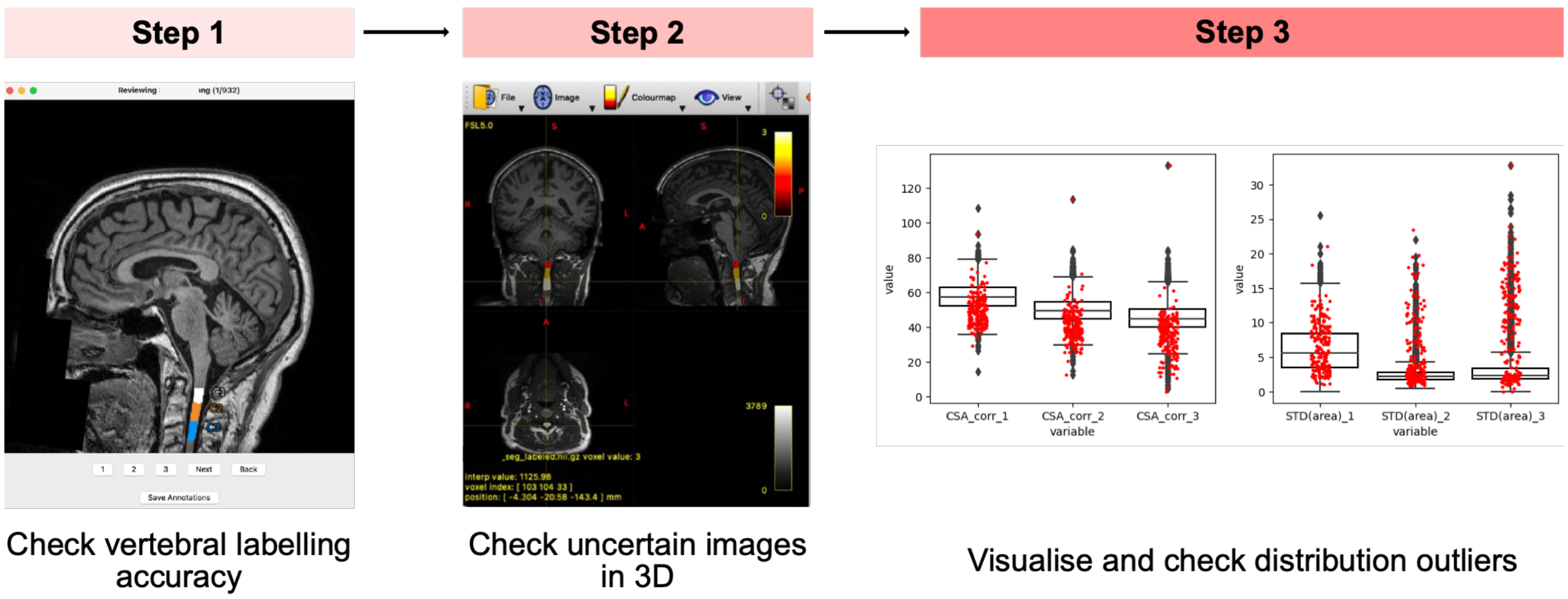

Supplementary Figure 42: The manual QC on vertebral labels follow a systematic 3-step. Step 1: visual inspection on vertebral labels generated using sct_qc. Step 2: check uncertain images in 3D. Step 3: visualise failed QC cases in distribution and check any unlabelled outliers. Reproduced by kind permission of UK Biobank ©.

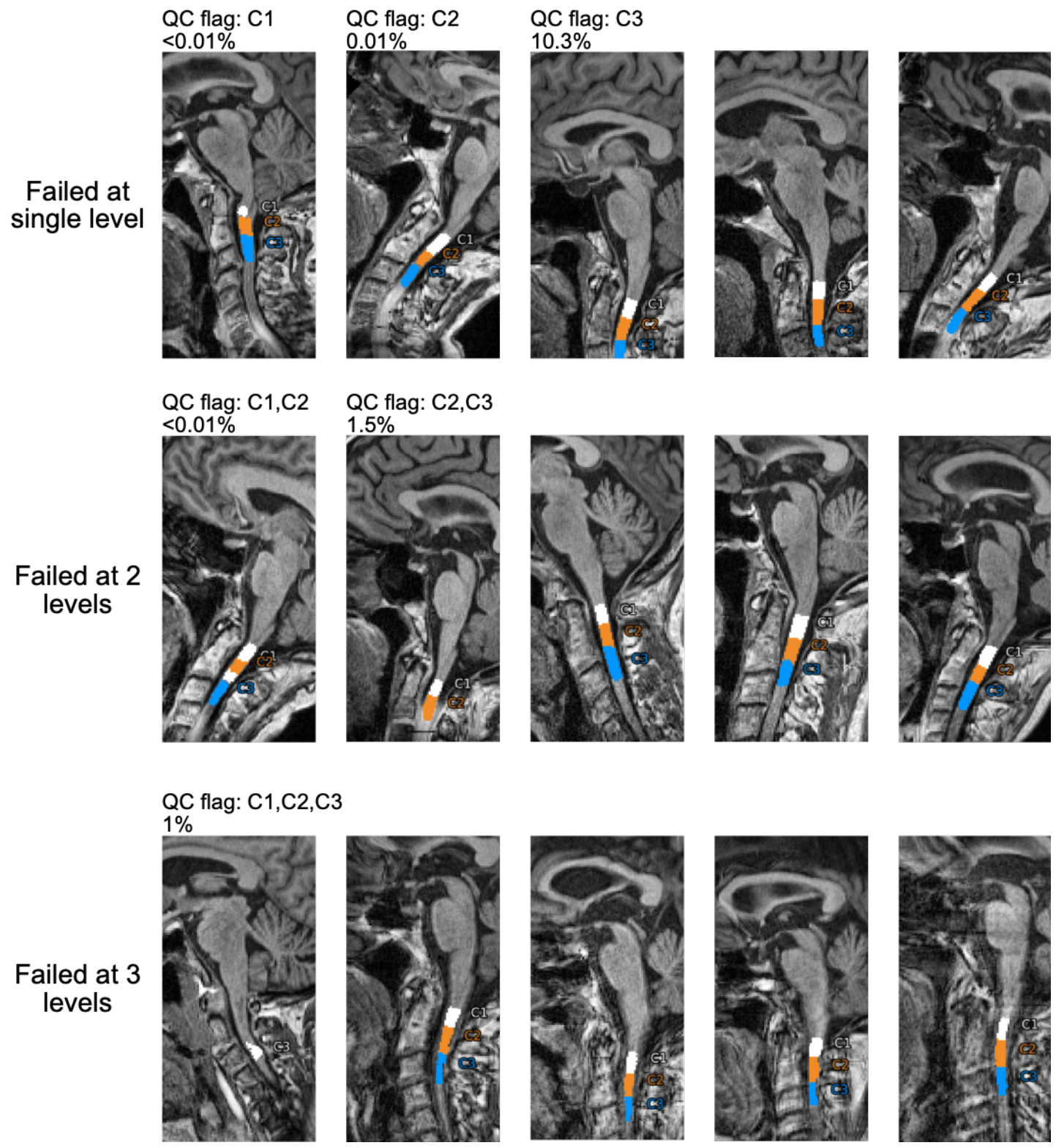

Supplementary Figure 43: Examples of different QC failure scenarios, including cases with failure at a single vertebral level and those with failures across multiple levels, along with their corresponding proportions in the dataset. Reproduced by kind permission of UK Biobank ©.

14.3 Additional removal of noise between processing steps

To remove any points with inconsistent values between processing steps, we compared shape metrics derived from the segmentation mask and those from the vertebral label image. Residuals between the two were calculated, and any point exceeding 5 standard deviations was excluded. These discrepancies were typically due to inaccuracies in the segmentation mask, which were corrected during vertebral labelling, as the model was trained to preserve the tubular structure of the spinal cord. This served as the final QC step for fine cleaning. Only a small percentage of noisy points were removed, with the majority showing high agreement. Although the metrics derived from the vertebral label image more closely resemble the results of manual QC, we did not use them for downstream analysis because they are not based on true axial slices and do not align with the image plane.

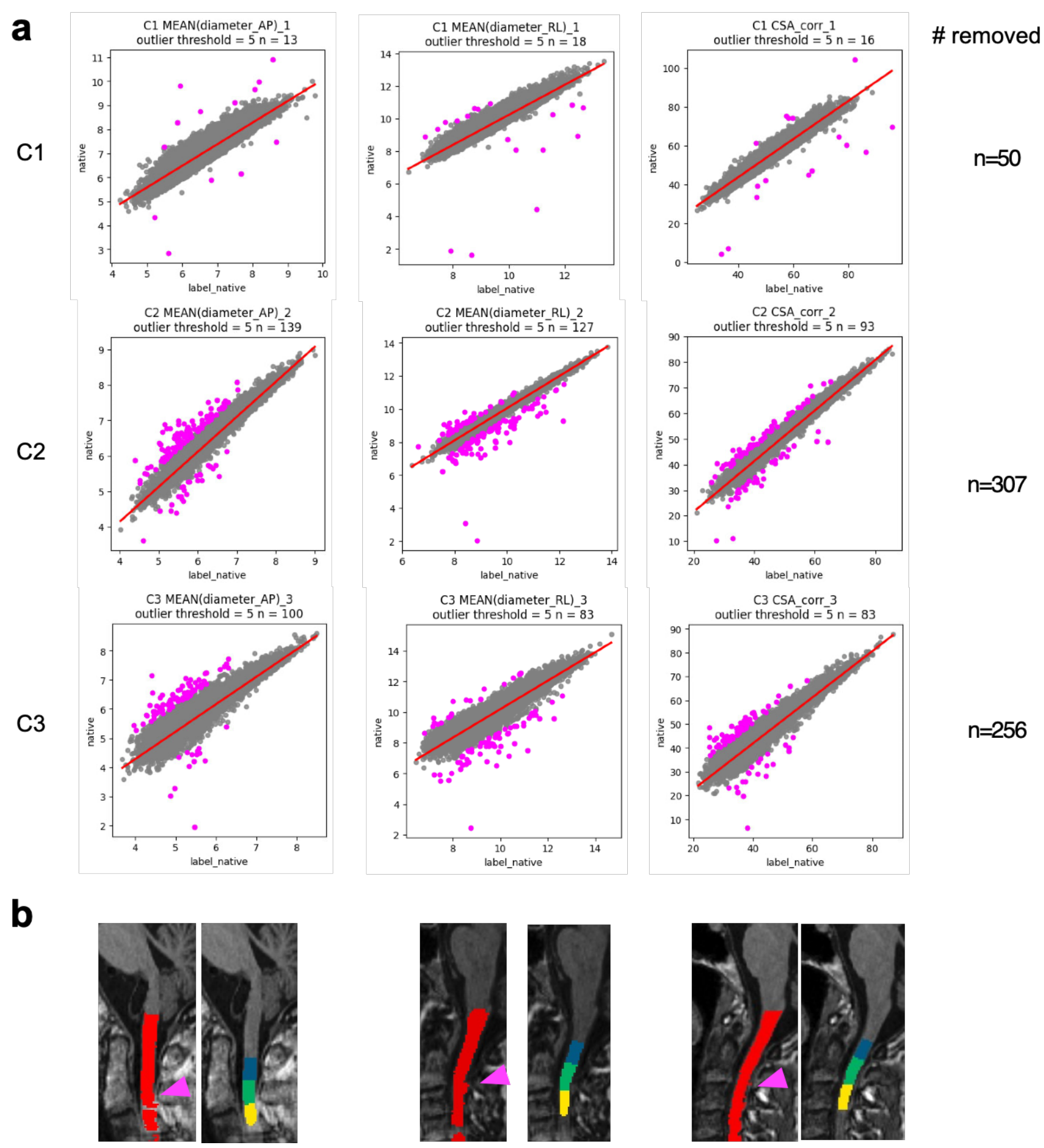

Supplementary Figure 44: (a) Scatterplot showing residuals between shape metrics derived from segmentation masks and vertebral labels. Points exceeding 5 standard deviations were excluded (pink). (b) Example images of excluded points, with arrows highlighting segmentation errors (left) corrected during vertebral labelling (right). Reproduced by kind permission of UK Biobank ©.

Supplementary References

1. De Leener, B. *et al.* PAM50: Unbiased multimodal template of the brainstem and spinal cord aligned with the ICBM152 space. *Neuroimage* **165**, 170–179 (2018).
2. Frostell, A., Hakim, R., Thelin, E. P., Mattsson, P. & Svensson, M. A review of the segmental diameter of the healthy human spinal cord. *Front. Neurol.* **7**, 238 (2016).
3. Alfaro-Almagro, F. *et al.* Image processing and Quality Control for the first 10,000 brain imaging datasets from UK Biobank. *Neuroimage* **166**, 400–424 (2018).
