# Supplementary material for: Genetic architecture of MRI-derived cervical spinal cord morphology reveals sensory-motor axis and systemic disease associations
Source: Nat Commun. 2026 Sep 16;17:10904. doi: 10.1038/s41467-026-77419-x (PMC13677749; doi:10.1038/s41467-026-77419-x)
Supplement: Supplementary file 2 [file 41467_2026_77419_MOESM2_ESM.pdf]

The Description of Additional Supplementary Files

Supplementary Data 1: Excluded conditions related to cervical spine congenital conditions and operative procedures.

Supplementary Data 2: Genome-wide significant independent loci for spinal cord shape metrics.

Supplementary Data 3: Lambda and LD Scrore regression intercepts values for GWAS.

Supplementary Data 4: Cross-reference of independent genome-wide significant variants with previously reported trait associations in the GWAS Catalog.

Supplementary Data 5: Genome-wide significant independent loci for spinal cord traits without adjustment for height.

Supplementary Data 6: Comparison of GWAS effect sizes and P-values with and without height adjustment.

Supplementary Data 7: The SNP-heritability estimates on observed scale from LDSC.

Supplementary Data 8: Gene c correlations of spinal cord phenotypes.

Supplementary Data 9: Phenotypic correlations of spinal cord phenotypes.

Supplementary Data 10: Significant SNPs-by-sex interaction effects

Supplementary Data 11: Gene c correlation between discovery and replica on GWASs of spinal cord phenotypes.

Supplementary Data 12: Discovery, replica on and meta-analysis results for genome-wide significant loci

Supplementary Data 13. Replica on of genome-wide significant lead SNPs for spinal cord phenotypes in the replica on cohort

Supplementary Data 14: Unique-gene counts by gene-based association method across 12 spinal cord phenotypes.

Supplementary Data 15: Unique-gene counts by gene-based association method spinal cord cervical level (C1/C2/C3) collapsed to 4 morphological measures.

Supplementary Data 16: Significant gene counts by test and measure (FDR < 0.05).

Supplementary Data 17: Significant gene counts by test and measure (Bonferroni P < 0.05).

Supplementary Data 18: Significant associations by test and phenotype (per-level breakdown).

Supplementary Data 19: Unique significant genes by test and phenotype (FDR < 0.05).

Supplementary Data 20: Unique significant genes by test and phenotype (Bonferroni P < 0.05).

Supplementary Data 21: Overlap of significant genes across gene-based tests.

Supplementary Data 22: Per-gene cross-test support summary.

Supplementary Data 23: COLOC / HEIDI breakdown by panel and by convergence er.

Supplementary Data 24: Gene-level colocalization and cross-test support for high-confidence genes.

Supplementary Data 25: FDR-significant MAGMA gene-based results.

Supplementary Data 26: FDR-significant mBAT-combo gene-based results.

Supplementary Data 27: FDR-significant SMR results using eQTLGen blood eQTL data.

Supplementary Data 28: FDR-significant SMR results using BrainMeta brain eQTL data

Supplementary Data 29: FDR-significant FUSION TWAS results using PsychENCODE weights.

Supplementary Data 30: FDR-significant FUSION TWAS results across GTEx v8 brain tissues.

Supplementary Data 31: 31.1-31.12 GSA-MiXeR and MAGMA gene-set enrichment analysis to each spinal cord morphology phenotype

Supplementary Data 32: Gene c correlations between spinal cord phenotypes and brain volumes

Supplementary Data 33: Phenotypic correlations between spinal cord phenotypes and brain volumes

Supplementary Data 34: Phenotypic associations between spinal cord phenotypes and Phecode diseases (minimum 100 cases) in European samples

Supplementary Data 35: Comparison of demographic characteristics and disease prevalence between UK Biobank imaging and non-imaging (PRS) cohorts.

Supplementary Data 36: Polygenic risk scores (PRS) associations with diseases that showed significant associations with spinal cord phenotypes

Supplementary Data 37: Gene c correlations between spinal cord phenotypes and diseases

Supplementary Data 38: Mendelian randomization (MR) causal effect estimates and sensitivity analyses between traits and spinal cord shape metrics

Supplementary Data 39: Mendelian randomization results using CAUSE for select phenotype associations.

Supplementary Data 40: Mendelian randomization (MR) causal effect estimates and sensitivity analyses between spinal cord shape metrics and traits

Supplementary Data 41: Conversion table mapping ICD-10 codes (retrieved from UK Biobank field) to Phecode X

Supplementary Data 42: List of complex human phenotypes used to estimate gene c correlations with spinal cord phenotypes.
