# Supplementary material for: Genetic architecture of MRI-derived cervical spinal cord morphology reveals sensory-motor axis and systemic disease associations
Source: Nat Commun. 2026 Sep 16;17:10904. doi: 10.1038/s41467-026-77419-x (PMC13677749; doi:10.1038/s41467-026-77419-x)
Supplement: Supplementary file 3 [file 41467_2026_77419_MOESM45_ESM.pdf]

nature portfolio

Corresponding author(s): Melanie Bahlo

Last updated by author(s): Aug 20, 2026

Reporting Summary

Nature Portfolio wishes to improve the reproducibility of the work that we publish. This form provides structure for consistency and transparency in reporting. For further information on Nature Portfolio policies, see our Editorial Policies and the Editorial Policy Checklist.

Statistics

For all statistical analyses, confirm that the following items are present in the figure legend, table legend, main text, or Methods section.

n/a	Confirmed	
☐	☒	The exact sample size (*n*) for each experimental group/condition, given as a discrete number and unit of measurement
☐	☒	A statement on whether measurements were taken from distinct samples or whether the same sample was measured repeatedly
☐	☒	The statistical test(s) used AND whether they are one- or two-sided<br>*Only common tests should be described solely by name; describe more complex techniques in the Methods section.*
☐	☒	A description of all covariates tested
☐	☒	A description of any assumptions or corrections, such as tests of normality and adjustment for multiple comparisons
☐	☒	A full description of the statistical parameters including central tendency (e.g. means) or other basic estimates (e.g. regression coefficient) AND variation (e.g. standard deviation) or associated estimates of uncertainty (e.g. confidence intervals)
☐	☒	For null hypothesis testing, the test statistic (e.g. *F*, *t*, *r*) with confidence intervals, effect sizes, degrees of freedom and *P* value noted<br>*Give P values as exact values whenever suitable.*
☒	☐	For Bayesian analysis, information on the choice of priors and Markov chain Monte Carlo settings
☒	☐	For hierarchical and complex designs, identification of the appropriate level for tests and full reporting of outcomes
☐	☒	Estimates of effect sizes (e.g. Cohen's *d*, Pearson's *r*), indicating how they were calculated

Our web collection on statistics for biologists contains articles on many of the points above.

Software and code

Policy information about availability of computer code

Data collection	No software were used for data collection.
Data analysis	The spinal cord image processing pipeline is publicly available at: https://github.com/art2mri/Enigma-SC<br><br>Publicly available software tools were used to perform genetic analyses and are referenced throughout the paper:<br>SCT (Spinal Cord Toolbox) v6.0, PLINK2 (v2.0.0-a.6.9), REGENIE (v2.1.0), GCTA-COJO (v1.94.4), LDSC (v1.0.1), FAVOR, MAGMA, GCTA / mBAT-combo (v1.94.1), COLOC (R package), SMR, HEIDI, GSA-MiXeR, PRS-CS, TwoSampleMR (R package), CAUSE (v1.2.0), ggseg (R package)

For manuscripts utilizing custom algorithms or software that are central to the research but not yet described in published literature, software must be made available to editors and reviewers. We strongly encourage code deposition in a community repository (e.g. GitHub). See the Nature Portfolio guidelines for submitting code & software for further information.

Data

Policy information about availability of data

All manuscripts must include a data availability statement. This statement should provide the following information, where applicable:

- Accession codes, unique identifiers, or web links for publicly available datasets
- A description of any restrictions on data availability
- For clinical datasets or third party data, please ensure that the statement adheres to our policy

The data used in the study are obtained from UKB Resource under Application Number 36610. All genetics, health related information, and imaging data described in this paper are publicly available to registered researchers through the UKB data-access protocol. Access to UK Biobank data is restricted to approved researchers with a valid application; additional information about registration for access to the data are available at: https://www.ukbiobank.ac.uk/enable-your-research/apply-for-access. The raw spinal cord phenotype data generated in this study will be returned to UK Biobank and researchers can access through UK Biobank upon approved application. Supplementary data are provided as Supplementary Data files uploaded with this submission. Source data are provided with the paper. The GWAS summary statistics generated in this study will be deposited in the GWAS Catalog upon publication.

Research involving human participants, their data, or biological material

Policy information about studies with human participants or human data. See also policy information about sex, gender (identity/presentation), and sexual orientation and race, ethnicity and racism.

Reporting on sex and gender	Sex was determined using genetic data. We obtained sex information from UK Biobank field Data-Field 22001. Sex was included as a covariate in all analyses.<br><br>For the primary analysis,19,552 (52.7%) individuals were female, 17,516 (47.3%) were male. Detailed demographic characteristics are reported in Supplementary Data 35.
Reporting on race, ethnicity, or other socially relevant groupings	Our analyses utilised grouping of individuals by ancestry, based on genetic similarity to individuals from the 1000 Genomes Project and Human Genome Diversity Panel. These broad continental ancestry assignments were generated through the Pan-UK Biobank project (https://pan.ukbb.broadinstitute.org/), and were obtained from UK Biobank returned dataset 2442.
Population characteristics	Among the individuals included in the primary analyses in this paper, 47.3% were male, and participants had a mean age of 64.0 years at imaging (SD = 7.69). Genetic information for participants was derived via genotyping with Affymetrix UK BiLEVE or UK Biobank arrays, and imputation to the TOPMed r2 reference panel. All individuals had complete information for the covariates included in the reported analyses: age, sex, age², genotyping batch, imaging site, ICV, height, inverted signal-to-noise ratio in T1-weighted images, and the first ten genetic ancestry principal components.
Recruitment	The UK Biobank is a prospective cohort study of over 500,000 individuals from across the United Kingdom, aged between 40 and 69 at recruitment. A range of phenotypic information and biological samples have been collected for all participants, in addition to linkage to electronic health records. At baseline, self-reported socio-demographic, lifestyle and health-related factors were collected, along with physical measures, and blood, urine and saliva samples.
Ethics oversight	Access to all UKB (https://www.ukbiobank.ac.uk/) data was granted on June 18th, 2019, application #36610. UKB has ethics approval from the North West Multi–centre Research Ethics Committee. This study was approved by the Walter and Eliza Hall Institute of Medical Research (WEHI), Human Research Ethics Committee (HREC reference 17/09LR).

Note that full information on the approval of the study protocol must also be provided in the manuscript.

Field-specific reporting

Please select the one below that is the best fit for your research. If you are not sure, read the appropriate sections before making your selection.

☒ Life sciences ☐ Behavioural & social sciences ☐ Ecological, evolutionary & environmental sciences

For a reference copy of the document with all sections, see nature.com/documents/nr-reporting-summary-flat.pdf

Life sciences study design

All studies must disclose on these points even when the disclosure is negative.

Sample size	The sample size was not pre-determined; instead, the analyses utilised the largest available dataset with both brain MRI data and genome-wide genetic data.
Data exclusions	Analyses were restricted to individuals with both genetic and brain MRI imaging data available, passing all quality control for these data, and with complete data for utilised covariates (see above).<br>To reduce potential confounding, we further excluded individuals with conditions affecting the cervical spine, as identified by ICD-10 diagnostic codes (Data-Field 41270) and operative procedure (OPCS-4, Data-Field 41272).<br>Primary analyses included individuals with European ancestries, with secondary analyses in individuals with African, East Asian, and South Asian ancestries. Individuals who were determined to be of any other ancestry, or mixed ancestries were excluded, due to insufficient sample

size to allow meaningful analyses.

Replication: Replication in independent samples was undertaken using the latest UK Biobank data release v20.5 (2025). Primary genetic analyses were conducted in individuals of European ancestry, with cross-ancestry replication within UK Biobank performed in individuals of African, East Asian, and South Asian ancestries.
In addition, we estimated polygenic risk scores (PRS) for each of the 12 spinal cord measures in the remaining UK Biobank participants without imaging data and tested these scores for association with disease outcomes.

Randomization: This is an observational study, and no randomization was undertaken.

Blinding: No blinding was undertaken for this study. No allocation to groups was made.

Reporting for specific materials, systems and methods

We require information from authors about some types of materials, experimental systems and methods used in many studies. Here, indicate whether each material, system or method listed is relevant to your study. If you are not sure if a list item applies to your research, read the appropriate section before selecting a response.

Materials & experimental systems

n/a	Involved in the study
☒	☐ Antibodies
☒	☐ Eukaryotic cell lines
☒	☐ Palaeontology and archaeology
☒	☐ Animals and other organisms
☒	☐ Clinical data
☒	☐ Dual use research of concern
☒	☐ Plants

Methods

n/a	Involved in the study
☒	☐ ChIP-seq
☒	☐ Flow cytometry
☐	☒ MRI-based neuroimaging

Plants

Seed stocks: *Report on the source of all seed stocks or other plant material used. If applicable, state the seed stock centre and catalogue number. If plant specimens were collected from the field, describe the collection location, date and sampling procedures.*

Novel plant genotypes: *Describe the methods by which all novel plant genotypes were produced. This includes those generated by transgenic approaches, gene editing, chemical/radiation-based mutagenesis and hybridization. For transgenic lines, describe the transformation method, the number of independent lines analyzed and the generation upon which experiments were performed. For gene-edited lines, describe the editor used, the endogenous sequence targeted for editing, the targeting guide RNA sequence (if applicable) and how the editor was applied.*

Authentication: *Describe any authentication procedures for each seed stock used or novel genotype generated. Describe any experiments used to assess the effect of a mutation and, where applicable, how potential secondary effects (e.g. second site T-DNA insertions, mosiacism, off-target gene editing) were examined.*

Magnetic resonance imaging

Experimental design

Design type: No task was used in the MRI scanner for this study.

Design specifications: No task was used in the MRI scanner for this study.

Behavioral performance measures: No task was used in the MRI scanner for this study.

Acquisition

Imaging type(s): Structural

Field strength: 3T

Sequence & imaging parameters: AMRI image acquisition was done following the UKB imaging protocol (https://biobank.ctsu.ox.ac.uk/crystal/crystal/docs/brain_mri.pdf). In brief, brain imaging was acquired across four identical imaging centres in the UK. T1-weighted (T1-w) structural scans were acquired using a 3D MPRAGE protocol (voxel size = 1 × 1 × 1 mm3, matrix size = 208 × 256 × 256, inversion time (TI)/TR = 880/2,000 ms, in-plane acceleration = 2, total scan time = 4 min 54 s).

Area of acquisition: Whole-brain including neck

Diffusion MRI ☐ Used ☒ Not used

Preprocessing

Preprocessing software	No preprocessing software was used. Uncropped T1-w images are used.
Normalization	No normalization was applied when generating phenotypes for GWAS. Although we tested normalization strategy by registering to PAM50 template in Section 2 of the Supplementary Information.
Normalization template	PAM50 template was used for evaluation purpose only to ensure method robustness.
Noise and artifact removal	The UKB standard QC pipeline was applied to exclude any images of poor quality and severe artefacts (labelled as unusable)
Volume censoring	No volume censoring was conducted as entire T1 scan was used as input for spinal cord processing.

Statistical modeling & inference

Model type and settings	N/A
Effect(s) tested	N/A

Specify type of analysis: ☐ Whole brain ☒ ROI-based ☐ Both

Anatomical location(s): Extraction of the spinal cord, followed by vertebral labeling of the C1-C3 levels using the optimised labeling methods https://github.com/art2mri/Enigma-SC

Statistic type for inference (See Eklund et al. 2016)	No cluster type statistical inferences were done with MRI data directly in this study.
Correction	No correction is applicable for the MRI data alone, but all data within the GWAS analysis was corrected using the Bonferroni approach.

Models & analysis

n/a	Involved in the study
☒	☐ Functional and/or effective connectivity
☒	☐ Graph analysis
☒	☐ Multivariate modeling or predictive analysis
