# Supplementary material for: Genetic architecture of MRI-derived cervical spinal cord morphology reveals sensory-motor axis and systemic disease associations
Source: Nat Commun. 2026 Sep 16;17:10904. doi: 10.1038/s41467-026-77419-x (PMC13677749; doi:10.1038/s41467-026-77419-x)
Supplement: Supplementary file 4 [file 41467_2026_77419_MOESM46_ESM.pdf]

nature portfolio

Peer Review File

Genetic architecture of MRI-derived cervical spinal cord morphology reveals sensory-motor axis and systemic disease associations

Corresponding Author: Professor Melanie Bahlo

This file contains all reviewer reports in order by version, followed by all author rebuttals in order by version.

Version 0:

Reviewer comments:

Reviewer #1

(Remarks to the Author)
This is an interesting paper with robust findings. However, I have several major comments on this paper.

First, literature review is not complete. Here are several closely related papers.

Shu, Juan, Rong Zheng, Julio Chirinos, Carlos Copana, Bingxuan Li, Zirui Fan, Xiaochen Yang et al. "Inferring multi-organ genetic connections using imaging and clinical data through Mendelian randomization." Nature Biomedical Engineering (2025): 1-16.
Jiang, Zhiwen, Patrick F. Sullivan, Tengfei Li, Bingxin Zhao, Xifeng Wang, Tianyou Luo, Shuai Huang et al. "The X chromosome's influences on the human brain." Science Advances 11, no. 4 (2025): eadq5360.
Zhao, B., Luo, T., Li, T., Li, Y., Zhang, J., Shan, Y., ... & Zhu, H. (2019). Genome-wide association analysis of 19,629 individuals identifies variants influencing regional brain volumes and refines their genetic co-architecture with cognitive and mental health traits. Nature genetics, 51(11), 1637-1644.

Please better situate your work within this literature and explicitly connect your key findings to the results and interpretations in these papers.

Second, the validation strategy could be substantially strengthened. The manuscript attempts to replicate the 179 identified variants in non-European UKB cohorts (Central/South Asian, African, and East Asian ancestry). While directional concordance is encouraging (up to 83.3% for certain traits in the East Asian cohort), none of the variants reach statistical significance, and the authors appropriately attribute this to limited power in currently available non-European samples. To improve validation, the authors should consider leveraging additional UKB participants (e.g., from newer data releases) and/or implementing a clear internal validation scheme by splitting the current sample into discovery and replication sets.

Third, there may be meaningful sampling differences between the UKB imaging cohort and UKB participants without imaging data. Such differences could reduce portability and predictive performance of PRS when applied outside the imaging subset. The authors should characterize these differences and, if possible, evaluate how they affect PRS performance (e.g., by comparing key demographics/clinical variables and conducting sensitivity analyses).

(Remarks on code availability)

Reviewer #2

(Remarks to the Author)
Sun et al. derived spinal cord IDPs using a machine learning pipeline and conducted a large-scale GWAS analysis. The authors further performed a range of downstream analyses, including heritability estimation, identification of significant loci, sex-stratified analysis, phenotypic and genetic correlations among spinal cord IDPs, and cross-trait analyses with other health-related outcomes. These analyses characterized the genetic architecture of spinal cord traits and explored their connections with brain phenotypes and related diseases. The scope of the study is extensive, and the manuscript is generally well written. Below are several comments that may help improve the clarity and overall quality of the manuscript.

1. Line 104: The authors report very high correlations (r^2 = 0.97–0.99) between the proposed machine learning–derived phenotypes and those obtained using existing methods. While this high concordance supports the validity of the approach, it would be helpful for the authors to more explicitly articulate the practical advantages of the current pipeline. Clarifying the advantages would better highlight the added value of this approach beyond its high agreement with established methods.

2. Lines 99 and 205: The manuscript reports both Pearson's and Spearman's correlation coefficients in different sections. Please clarify the rationale for choosing one metric over the other in each context.

3. Line 210: In the gene-based analysis, six approaches were used to identify significant genes associated with spinal cord phenotypes. However, the manuscript primarily reports the number of significant genes identified by each method, with limited detail about the genes themselves. Providing more information on key overlapping genes or biological insights may strengthen this section.

4. Line 239: The authors applied six approaches to prioritize genes associated with spinal cord phenotypes. Could the authors clarify why gene-set enrichment analysis was performed based solely on MAGMA results? Were the other gene-based analysis methods considered in pathway analyses? Additionally, it would be helpful to indicate whether the enriched pathways involved key genes identified consistently across multiple methods.

5. Lines 273-275 and 299-301: Some reported associations do not include correlation coefficients, and in certain cases, only multiple-testing-adjusted P-values are provided. Reporting both the effect sizes and the corresponding original P-values would improve the interpretability and transparency of the reported associations.

(Remarks on code availability)
The GitHub repository is well prepared.

Reviewer #3

(Remarks to the Author)
I co-reviewed this manuscript with one of the reviewers who provided the listed reports. This is part of the Nature Communications initiative to facilitate training in peer review and to provide appropriate recognition for Early Career Researchers who co-review manuscripts.

(Remarks on code availability)

Reviewer #4

(Remarks to the Author)
Reviewer comments on the manuscript "Genetic architecture of ... spinal cord morphology..."

The authors develop a novel deep-learning approach to segment and identify the very first levels of the spinal cord from brain MRI, apply it to UKB data to compute IDPs, then investigate these using GWAS. They find significant heritabilities and multiple associations with genomic variants. While representing a significant advance in investigations of the genetics influencing spinal cord, the study has some limitations and areas that need clarification. My main comments are as follows:

1) Brain MRI has inherently limited cervical coverage; even the segments that are covered are often plagued by distortion and susceptibility artefacts. With large voxel size (1mm), that imposes additional challenges. Ensuring accurate measures is surely difficult. The authors provide (for review only) their working manuscript that details the methods and include Dice/Jaccard coefficients, along with other metrics for accuracy. However, readers will not have access to these until (and if) the other manuscript is published.

2) That more caudal segments of the upper cervical spinal cord were observed to have lower heritability likely demonstrates the problem: the more caudal segments are precisely those that have poorer coverage and are subjected to most artefacts. These are also where sample size becomes smaller. Such artefacts may well be present also in the more cranial parts, just to a lesser extent.

3) The authors explain that from a biological standpoint, e.g., due to proximity to brain-stem. However, such biological explanations are only warranted after ensuring confounds as above are not present. The more likely explanation for the progressively reduced "signal" from C1 to C3 is lack of coverage and artefacts.

4) Use of composite phenotypes: excentricity is a mixture (ratio) of AP and RL, which are in themselves traits that, as the authors observed, are influenced by shared genetic factors. It does not come as a surprise, therefore, that excentricity has mostly negative genetic correlations with RL and AP across levels (Supplementary Table, in osf.io). This is a form of mathematical coupling. Ideally, the authors would seek primary phenotypes that are as uniquely genetically determined as possible, as opposed to trait mixtures as ECC.

5) The authors are commended for their investigations with MS and neuropathies, but these are known to affect the spinal cord. Sure, spinal cord could be a biomarker, but they may reflect more a consequence of the disease than a cause. I'm not sure that the "biomarker" interpretation is a valuable one, or even the best interpretation. Biomarker for risk? Biomarker for disease progression? That said, studying genetic influences on the spinal cord is a valuable end on its own, with or without such disease biomarker interpretation, which is a weak one.

6) Stil on this, causal mechanisms pervade the discussion, e.g., "these findings suggest that spinal cord morphology may reflect broader systemic health and disease susceptibility." (p.10), "results suggest potential shared genetic architecture between spinal cord shape metrics and a range of disease categories" (p.11, at the top). Yet, the authors themselves admit that "Thus, our results do not provide robust evidence of a causal effect of PD or BMI on spinal cord phenotypes" (p.11, at the bottom). The overall story of the paper isn't well tied within a consistent interpretation.

7) Perhaps I'm missing among the various files submitted and table in the osf.io website but do the authors report SNPs differentially associated with spinal cord traits with and without height as a confound? It matters as both sets of variants are of interest: those that affect spinal cord possibly also impacting height, and those that are uniquely associated with the spinal cord itself with no impact on height. Supplementary Table 5 (Excel file) shows the correlations between the two analyses, but that isn't interesting. The variants themselves are.

8) Some comments in the discussion are highly speculative, e.g., role of kidneys, synaptic homeostasis, etc. The fact, is, we don't know and won't know until the pathways involving these genes are elucidated one by one. As long as we keep studying statistical associations, we won't learn much more (I blame the damage caused by Systems Biology across multiple basic sciences, but I digress...).

All and all, this is an interesting study, timely given the data availability. The authors have clearly put a lot of effort in it, e.g., by performing multi-ancestry replication (even if likely underpowered, but there are no other datasets like UKB), by stratifying by sex, and by going further than just doing a GWAS (colocalization, gene prioritization, PheWAS, MR, etc). They also make the code available on Github, which is a plus. I believe my critiques above are addressable, except the inherent issues related using brain MRI to assess non-brain tissue. Perhaps the uncertainty in the more caudal segments can be addressed by modifications in the deep-learning method for segmentation, which is the topic of their other manuscript.

(Remarks on code availability)

Version 1:

Reviewer comments:

Reviewer #2

(Remarks to the Author)
The authors have made useful revisions in response to my previous comments, including clarifying the pipeline's analytical rationale, explaining the choice of Pearson over Spearman correlations, and adding biological interpretation for key candidate genes. These revisions improve the manuscript's transparency. Several points warrant further consideration to strengthen the paper.

The rationale for the gene-set enrichment analysis still needs clarification. The authors state that gene-set enrichment was not applied to genes derived from the other five methods because many alternative pathway analyses rely on hypergeometric tests that may show p-value inflation due to gene length, gene density, co-expression, and LD. However, these factors mainly affect the upstream probability that a gene is selected by a given gene-prioritization method, whereas the hypergeometric test itself simply evaluates overlap between a gene list and a background set. Therefore, the authors should clarify whether their concern is that the other five methods produce heterogeneous outputs that are not suitable for a unified pathway enrichment framework, or whether there are specific statistical limitations that prevent pathway analysis based on those gene sets.

There are also several minor issues in the gene-set enrichment section. The authors report both 432 unique significant genes and 434 genes significantly associated with a spinal cord phenotype in at least one approach; this discrepancy should be checked and corrected. For the 37 overlapping genes, it would be clearer to specify whether the overlap was calculated using the 96 high-confidence genes, the 218 genes supported by at least two approaches, or the full 432/434-gene set. If the overlap is intended only as a descriptive comparison, this should be stated clearly; otherwise, additional statistical support would be needed to interpret it as evidence of enrichment or convergence beyond chance. In addition, the phrase "organ development pathways" is broad; since the response specifically mentions kidney and renal development

pathways, the revised text should use more precise wording.

(Remarks on code availability)

Reviewer #3

(Remarks to the Author)
I co-reviewed this manuscript with one of the reviewers who provided the listed reports. This is part of the Nature Communications initiative to facilitate training in peer review and to provide appropriate recognition for Early Career Researchers who co-review manuscripts.

(Remarks on code availability)

Reviewer #4

(Remarks to the Author)
I'd like to thank the authors for their responsive revision. The bulk of my concerns have been addressed.

I have one remaining minor point, which I believe is easily resolvable: The "biomarker" framing has been toned down, yet "potential radiological biomarker" survives in the Discussion and "potent as a biomarker" in the Limitations. A natural question still arises on biomarker for what? There are some elements in the ms that allude to "a broad range of disease states", after mentions to "viral infections", "musculoskeletal disorders", "gout arthropathies", and "essential hypertension". But if said biomarker is that unspecific, how helpful is it? Addressing this is easy: studying genetic influences on the spinal cord is valuable on its own, regardless of whether there is a "biomarker" interpretation for something or not. In fact, I see no need to mention the idea of biomarkers at all.

(Remarks on code availability)
N/A

Open Access This Peer Review File is licensed under a Creative Commons Attribution 4.0 International License, which permits use, sharing, adaptation, distribution and reproduction in any medium or format, as long as you give appropriate credit to the original author(s) and the source, provide a link to the Creative Commons license, and indicate if changes were made.
In cases where reviewers are anonymous, credit should be given to 'Anonymous Referee' and the source.
The images or other third party material in this Peer Review File are included in the article’s Creative Commons license, unless indicated otherwise in a credit line to the material. If material is not included in the article’s Creative Commons license and your intended use is not permitted by statutory regulation or exceeds the permitted use, you will need to obtain permission directly from the copyright holder.
To view a copy of this license, visit https://creativecommons.org/licenses/by/4.0/

REVIEWER COMMENTS

We thank both reviewers for their considered and useful comments. A point-by-point response is provided below, with the reviewers' original comments in italics. Due to the excessive file size, the updated supplementary tables are available for downloading at online repository OSF: https://osf.io/9s6gk/.

Reviewer #1 (Remarks to the Author):

This is an interesting paper with robust findings. However, I have several major comments on this paper.

1. *First, literature review is not complete. Here are several closely related papers. Shu, Juan, Rong Zheng, Julio Chirinos, Carlos Copana, Bingxuan Li, Zirui Fan, Xiaochen Yang et al. "Inferring multi-organ genetic connections using imaging and clinical data through Mendelian randomization." Nature Biomedical Engineering (2025): 1-16. Jiang, Zhiwen, Patrick F. Sullivan, Tengfei Li, Bingxin Zhao, Xifeng Wang, Tianyou Luo, Shuai Huang et al. "The X chromosome's influences on the human brain." Science Advances 11, no. 4 (2025): eadq5360. Zhao, B., Luo, T., Li, T., Li, Y., Zhang, J., Shan, Y., ... & Zhu, H. (2019). Genome-wide association analysis of 19,629 individuals identifies variants influencing regional brain volumes and refines their genetic co-architecture with cognitive and mental health traits. Nature genetics, 51(11), 1637-1644. Please better situate your work within this literature and explicitly connect your key findings to the results and interpretations in these papers.*

Thank you for this beneficial suggestion. We have now added these suggested references to our introduction paragraph as reference 19 and 23.

2. *Second, the validation strategy could be substantially strengthened. The manuscript attempts to replicate the 179 identified variants in non-European UKB cohorts (Central/South Asian, African, and East Asian ancestry). While directional concordance is encouraging (up to 83.3% for certain traits in the East Asian cohort), none of the variants reach statistical significance, and the authors appropriately attribute this to limited power in currently available non-European samples. To improve validation, the authors should consider leveraging additional UKB participants (e.g., from newer data releases) and/or implementing a clear internal validation scheme by splitting the current sample into discovery and replication sets.*

Thank you for this constructive suggestion. We agree that strengthening the validation strategy is important. In response, we have substantially expanded our replication analyses by adding newer data release in 2025 (v20.5). An additional 35,530 images were processed for the replication analysis.

First, we performed replication in European ancestry individuals using additional participants, ensuring no overlap with the discovery cohort. After imaging and genetic quality control, the replication sample included 27,073 European individuals at the C1 spinal level, 26,234 at C2, and 22,450 at C3. We have added a new section titled "Associations in replication GWAS" in results and

supplementary materials, demonstrating strong consistency between discovery and replication results. To summarise, we observed strong genetic correlations (rg = 1.00–1.12), complete directional concordance across all variants, and a high proportion of detected loci from the discovery cohort showing nominal significance (84.6% - 100%), as well as with a substantial subset (28.6% - 69.2%) reaching genome-wide significance ($P < 5e-8$). These findings support the robustness and reproducibility of our results in a well-powered, ancestry-matched replication setting.

Second, we expanded the non-European ancestry analyses (Central/South Asian, African, and East Asian cohorts) by including the updated imaging dataset, increasing sample sizes and modestly improving power. Directional concordance ranged from 38.5% (C2 CSA in AFR) to 100% (C3 RL diameter in C/SA and C3 AP diameter in AFR), with overall higher concordance observed compared to the previous analysis. However, most variants did not reach statistical significance in non-European cohorts, likely reflecting the still limited sample sizes (maximum $n \approx 893$).

Together, these analyses significantly strengthen the replication framework and further support the robustness of our findings, while highlighting the need for larger and more diverse cohorts for well-powered cross-ancestry replication.

3. *Third, there may be meaningful sampling differences between the UKB imaging cohort and UKB participants without imaging data. Such differences could reduce portability and predictive performance of PRS when applied outside the imaging subset. The authors should characterize these differences and, if possible, evaluate how they affect PRS performance (e.g., by comparing key demographics/clinical variables and conducting sensitivity analyses).*

We thank the reviewer for this important point. We agree that systematic differences between the UK Biobank imaging cohort and the broader UK Biobank population may influence the portability of PRS derived from imaging-based GWAS.

To address this, we compared key demographic characteristics of the discovery data (imaging release v15.1), replication data (v20.5), and non-imaged participants (Supplementary Table 24). The imaging and non-imaging subsets were broadly comparable, with similar sex distributions (52.6–54.3% female in imaging cohorts and 54.3% in the non-imaging sample). Participants in the imaging cohorts were slightly younger at baseline (mean age 54–55 years) compared to the non-imaging sample (57 years). The mean age at imaging was higher in the more recent imaging release than in the initial release (Supplementary Table 24).

In addition, we calculated the disease prevalence of the 47 diseases we tested. Overall imaged and non-imaged cohorts had similar disease prevalences, except for viral infectious diseases, which showed substantially lower prevalences in the non-imaged cohort. This difference is mainly due to a data collection artefact, as self-reported information for these conditions, including chickenpox, rubella, and measles was recorded during the follow-up imaging visits, but were not collected for participants who only attended the baseline visit. We have included a plot in Supplementary Figure 39 to illustrate this bias.

We recalculated the PRS Phecode associations removing the imaged participants in the replication data and updated in Supplementary Figure 37. 96.08% of the significant PRS disease associations from the discovery study showed concordant effect sign (direction), with rubella as the only exception.

Reviewer #2 (Remarks to the Author):

Sun et al. derived spinal cord IDPs using a machine learning pipeline and conducted a large-scale GWAS analysis. The authors further performed a range of downstream analyses, including heritability estimation, identification of significant loci, sex-stratified analysis, phenotypic and genetic correlations among spinal cord IDPs, and cross-trait analyses with other health-related outcomes. These analyses characterized the genetic architecture of spinal cord traits and explored their connections with brain phenotypes and related diseases. The scope of the study is extensive, and the manuscript is generally well written. Below are several comments that may help improve the clarity and overall quality of the manuscript.

1. *Line 104: The authors report very high correlations (r^2 = 0.97–0.99) between the proposed machine learning–derived phenotypes and those obtained using existing methods. While this high concordance supports the validity of the approach, it would be helpful for the authors to more explicitly articulate the practical advantages of the current pipeline. Clarifying the advantages would better highlight the added value of this approach beyond its high agreement with established methods.*

We thank the reviewer for the opportunity to clarify this point, as the reported high correlation was intended as an internal robustness assessment rather than as a benchmark against established external pipelines. Specifically, our entire processing workflow consists of three distinct stages (Refer to Fig.1: segmentation, vertebral labelling, and metric extraction) and this alternative pipeline comparison focused solely on the third stage: the extraction of metrics in either atlas or subject space, rather than comparing against other established methods.

To further clarify, the first segmentation step remains consistent with existing methodologies using the *deepseg* algorithm within the Spinal Cord Toolbox (SCT). The actual novelty and added value of the newly proposed Enigma-SC pipeline lie in the second stage of processing: the vertebral labelling. While established methods are technically robust in segmentation, their labelling components frequently require extensive manual correction. To date, only one other study utilised the UKB dataset for spinal cord analysis. 804 images free from severe artifacts were processed; however, this workflow still required manual intervention to ensure labelling accuracy (Bedard and Cohen-Adad, 2022). Thus, direct benchmarking using large dataset against established methods remained challenging because fully automated processing has not yet been established as a standard practice in the field. Our approach addresses this technical bottleneck by implementing a newly trained deep learning model for fully automatic and robust vertebral labelling, benefitting from a valuable training set containing severe spinal cord pathological cases. This advancement enables fully automated processing of the entire UK Biobank cohort, a capability not currently available in any established semi-automatic pipelines.

After vertebral labelling, we sought to ensure that our final of phenotype extraction was robust and independent of coordinate space. We have now expanded the Results section to more clearly define this robustness testing and avoid any potential confusion regarding these correlation values. “At the metric extraction stage, additional robustness tests were conducted to ensure our final set of phenotypes extracted was robust and independent of coordinate space. Spinal cord phenotypes at C2 and C3 showed high cross-method consistency across all traits (r^2 = 0.97-0.99) using the subject- and atlas-based analysis. Notably, extraction in the subject space yield better C1 detection and eliminated potential registration errors.” We also expanded on this point in the second paragraph of the Discussion to further highlight these practical advantages of the Enigma-SC pipeline.

2. *Lines 99 and 205: The manuscript reports both Pearson’s and Spearman’s correlation coefficients in different sections. Please clarify the rationale for choosing one metric over the other in each context.*

We thank the reviewer for this comment. We used Spearman’s correlation in analyses sensitive to outliers or non-normality, acknowledging that this method loses some power in comparison to Pearson’s correlation as a trade off, and Pearson’s correlation, when possible, to quantify linear concordance between continuous measures. We agree that the rationale behind these choices were not explicitly discussed in detail in the manuscript methods.

Specifically, Spearman’s correlation was used in settings where robustness to outliers and non-normality was present, including: (i) assessing consistency between first and second scans, where imaging artefacts introduced outliers, and (ii) comparing effect sizes between European and non-European samples across the 179 significant loci, where extreme values may be present. In contrast, Pearson’s correlation was used to assess linear concordance between continuous estimates, including (i) agreement between GWAS summary statistics with and without height adjustment (effect size and p-values) where the data follow normal distribution; and (ii) concordance of sex-specific SNP-based heritability estimates across the 12 traits. For the latter, this was also assessed using Spearman’s correlation ($r = 0.94$, $P < 2.2e-16$), supporting a strong positive relationship. While both methods may be applicable in this case, the results were comparable, given that this analysis only used 12 data points.

We have revised the manuscript accordingly in instances where Spearman’s correlation was chosen, for example: “Spearman’s correlations (robust to outliers) between the first and second scans were calculated to assess repeatability, stratified by scan interval (Supplementary Section 2.1).”

3. *Line 210: In the gene-based analysis, six approaches were used to identify significant genes associated with spinal cord phenotypes. However, the manuscript primarily reports the number of significant genes identified by each method, with limited detail about the genes themselves. Providing more information on key overlapping genes or biological insights may strengthen this section.*

We agree that highlighting key genes identified in the gene-based analysis adds biological depth to the manuscript. Previously we mainly reported the number of genes in the results, and we highlighted some plausible gene candidates in the discussion:

"Our gene-based analysis identified 96 high-confidence genes likely contributing to cervical spinal cord morphology variation … Among these, *SGTB*, *TGFA*, and *ROR2* emerged as top ranked and biologically plausible gene candidates. For example, *SGTB*, encoding a small tetratricopeptide repeat co-chaperone, showed strong evidence of colocalization in brain tissues with CSA and diameters. *SGTB* has a key role in shaping neurite morphology and promoting neuron survival and has been implicated in GWAS of neuroimaging phenotypes and white matter integrity. Meanwhile, TGFA promotes proliferation and survival in epithelial and neural tissues during development and is expressed in the developing spinal cord. *ROR2* is essential for axial skeletal development and vertebral patterning, with mutations causing autosomal recessive Robinow syndrome characterized by vertebral defects."

We have revised our manuscript by adding a short summary in the results highlighting these biologically plausible candidates:

"These candidate genes have important implications in diverse functions such as neuronal survival and neurite morphology, neural tissue proliferation during development, and axial skeletal and vertebral patterning."

4. *Line 239: The authors applied six approaches to prioritize genes associated with spinal cord phenotypes. Could the authors clarify why gene-set enrichment analysis was performed based solely on MAGMA results? Were the other gene-based analysis methods considered in pathway analyses? Additionally, it would be helpful to indicate whether the enriched pathways involved key genes identified consistently across multiple methods.*

Our gene-set enrichment analyses used MAGMA-derived gene-level statistics because the underlying approach, GSA-MiXeR, is designed to operate on MAGMA-based gene association outputs. However, we would like to note the gene-set enrichment results are not solely based on MAGMA results. Rather, GSA-MiXeR estimates the heritability of individual genes which can be used in a partitioned heritability framework to assess the fold enrichment of gene heritability in predefined gene sets (in our case, gene ontology categories). As recommended by the authors of GSA-MiXeR, we filtered the fold enrichment results on significant MAGMA results to improve interpretability. The full distribution of fold enrichment results (i.e., without MAGMA filtering) is available in Supplementary Table 20.1-20.12). We did not consider other pathway analysis approaches as most of these are based on hypergeometric tests which are prone to (p-value) inflation due to their inability to deal with gene characteristics (i.e., gene length, gene density, gene co-expression) and correlated effects (linkage disequilibrium).

To investigate whether the enriched pathways involved key genes identified consistently across multiple methods, we intersected gene members for significantly enriched pathways (based on fold enrichment from GSA-MiXeR; Supplementary Tables 20.1-20.12) with our list of candidate effector genes (Supplementary Table 19). We found 37 overlapping genes between our prioritised list of effector genes and gene members of enriched pathways. The key insight from this gene overlap is the enrichment of kidney and renal development pathways is driven by a core set of significant genes from our gene-based analysis (OSR1, BMP7, PKD2, WNT9B, and PDGRFA) that are significant

in at least one of the spinal cord phenotypes tested. Top-ranked pathways also contain significant gene-based results (e.g., NBL1 in GOBP_SEQUESTERING_OF_BMP_IN_EXTRACELLULAR_MATRIX). We have added a sentence in the results section to highlight these observations:

"We found 37 overlapping genes between our prioritized list of candidate genes (Supplementary Table 19) and gene members of enriched gene sets. The gene overlap showed the enrichment of organ development pathways is driven by a core set of significant genes from our gene-based analysis (OSR1, BMP7, PKD2, WNT9B, and PDGRFA) that are significant in at least one of the spinal cord phenotypes tested."

5. *Lines 273-275 and 299-301: Some reported associations do not include correlation coefficients, and in certain cases, only multiple-testing-adjusted P-values are provided. Reporting both the effect sizes and the corresponding original P-values would improve the interpretability and transparency of the reported associations.*

For tests that used FDR/Benjamini-Hochberg, we have now updated the raw P-values in additional to FDR corrected P-values in our results. For tests that used Bonferroni Correction, only raw P-values were reported in the text.

Reviewer #3 (Remarks to the Author):

I co-reviewed this manuscript with one of the reviewers who provided the listed reports. This is part of the Nature Communications initiative to facilitate training in peer review and to provide appropriate recognition for Early Career Researchers who co-review manuscripts.

4. Reviewer #4 (Remarks to the Author):

Reviewer comments on the manuscript "Genetic architecture of ... spinal cord morphology..."

The authors develop a novel deep-learning approach to segment and identify the very first levels of the spinal cord from brain MRI, apply it to UKB data to compute IDPs, then investigate these using GWAS. They find significant heritabilities and multiple associations with genomic variants. While representing a significant advance in investigations of the genetics influencing spinal cord, the study has some limitations and areas that need clarification. My main comments are as follows:

1. *Brain MRI has inherently limited cervical coverage; even the segments that are covered are often plagued by distortion and susceptibility artefacts. With large voxel size (1mm), that imposes additional challenges. Ensuring accurate measures is surely difficult. The authors provide (for review only) their working manuscript that details the methods and include Dice/Jaccard coefficients, along with other metrics for accuracy. However, readers will not have access to these until (and if) the other manuscript is published.*

We agree with the reviewer that the limited coverage of the spinal cord is indeed a major challenge in this project, which required significant improvements to established spinal cord processing pipelines. To address this, we implemented a newly trained model that enabled a fully automated pipeline (vs. previously semiautomatic correction was required). The robustness of this model was significantly enhanced by leveraging a training dataset that included clinical cases with severe spinal cord pathology. To highlight the advancements of this pipeline for the UKB data, it enables a fully automatic workflow that is highly robust to anatomical variation, and it maintains the tubular structure of the spinal cord to ensure accurate labelling even in cases where segmentation masks are fragmented due to image artifacts (Fig. S8). While the accompanying technical manuscript has currently undergone a few rounds of review process, our related open-source software has been active on GitHub for over two years with step-by-step instructions to run the full test. The full information on the model training and testing is beyond the scope of the current manuscript, but access to the pipeline is available through GitHub so readers can access and run analyses while the other manuscript is pending publication.

Although coverage is limited and image quality varies within the spinal cord, the UK Biobank remains a valuable asset for this analysis. In the current manuscript, we have tailored specific methodological considerations for this GWAS application, such as our vertebral-level specific QC approach and a registration-free pipeline, to better suit the large-scale genetic analysis. We hope our findings, including rigorous validation, QC, and now additional replication, showed that impactful discovery is possible despite these limitations. LDSC intercept values and genetic inflation factors indicate minimal inflation, confirms that with proper QC, C3 phenotypes are robust and well-suited for GWAS. In this revised version, our replication analyses further demonstrated that despite C3 having worse imaging quality, GWAS results of C3 spinal cord were highly replicable (complete directional concordance across all variants, and all C3 loci identified in the discovery GWAS reached nominal significance in the replication). We agree interpreting differences between vertebral levels needs to be taken with caution, so we expanded in the discussion section on the

inherent limitation of the imaging data quality, which will be further explained in response to comments #2 and #3 below.

2. *That more caudal segments of the upper cervical spinal cord were observed to have lower heritability likely demonstrates the problem: the more caudal segments are precisely those that have poorer coverage and are subjected to most artefacts. These are also where sample size becomes smaller. Such artefacts may well be present also in the more cranial parts, just to a lesser extent.*

We thank the reviewer for this insightful comment. We agree that the decreasing trend of heritability in more caudal cervical spinal cord segments cannot be attributed to biological factors without ruling out image quality variability. We have now revised our discussion, removing "The observed decline in heritability from C1 to C3 across all phenotypes may be attributed to both underlying genetic trends and decreasing sample size from C1 to C3. Notably, C1 lies closest to the brainstem, a highly heritable structure, which may explain its stronger genetic signal. Scans covering regions beyond C3 will help to further explore these patterns". We further acknowledge that the inherent limitations of the UK Biobank imaging make it challenging to fully disentangle these effects across specific vertebral levels. We have added this as a limitation in our Discussion. We highlight that while our current findings provide a foundation, future studies with extended coverage or specialised spinal cord pipelines will be better positioned to explore these variations in greater detail. "While we observed a decline in both GWAS hits (partially due to decrease sample size) and deceasing heritability from C1 to C3, these trends can be attributed to worse image quality of the C3 level. Fully disentangling biological effects from data quality factors is challenging with the UKB data, thus interpretations of these inter-level trends should be approached with caution."

As an attempt to investigate these effects using the UK Biobank, we performed additional sensitivity analyses restricting heritability estimation to a smaller set of individuals who had data available across all three of C1-C3, both in the discovery and replication cohorts. As shown in Fig. S20, heritability estimates were highly consistent between analyses using all available samples and those restricted to the same individuals, as well as between the discovery and replication datasets. Notably, the pattern of decreasing heritability towards more caudal segments was preserved in the discovery cohort under same-sample analyses and was independently replicated in both the replication cohort and its corresponding same-sample analyses. Despite the consistent trends observed, no definitive conclusions can be drawn at this time. Future studies with more consistent image quality for the C1–C3 regions are necessary to further investigate these findings. We updated the "Heritability and genetic correlation of spinal cord shape metrics" results section as follows:

"This pattern was consistently observed in analyses of the discovery cohort restricted to the same individuals across segments and was also replicated in both the replication cohort and its corresponding same-sample analyses (Fig. S20)."

3. *The authors explain that from a biological standpoint, e.g., due to proximity to brain-stem. However, such biological explanations are only warranted after ensuring confounds as above are not present. The more likely explanation for the progressively reduced "signal" from C1 to C3 is lack of coverage and artefacts.*

We appreciate the reviewer's observation and agree that technical confounds, such as artifacts and limited coverage, likely contribute to the reduced signal observed from C1 to C3. Accordingly, we have revised our interpretation and added this as a formal limitation in the Discussion (see answer in response to comment #2 above). We chose to preserve C3 and analysed C1-C3 as distinct levels to investigate vertebral-specific signals, different from standard neuroimaging studies where C2-C3 is typically analysed jointly. From a biological perspective, the spinal column is anatomically specialised and may shape the spinal cord, with genes differentially influencing specific vertebral levels. As this is the first GWAS of its kind, we sought to maximise the information captured from the available data. This approach is supported by our results: C3 yielded seven unique loci and a distinct phenotypic association with mononeuritis of the upper limb that were not present in C1 or C2, suggesting that there may be unique biological signals detectable even within this technically challenging region. Nevertheless, we recognise the difficulty in making biological interpretations without fully ruling out technical confounds and have included a clear cautionary statement in the Discussion to highlight this limitation as "While we observed a decline in both the number of GWAS associations (partially due to decreased sample size) and decreasing heritability from C1 to C3, these trends can be influenced by worse image quality of the C3 level. Fully disentangling biological effects from data quality factors is challenging with the UKB data, thus interpretations of these inter-level trends should be approached with caution."

4. *Use of composite phenotypes: eccentricity is a mixture (ratio) of AP and RL, which are in themselves traits that, as the authors observed, are influenced by shared genetic factors. It does not come as a surprise, therefore, that eccentricity has mostly negative genetic correlations with RL and AP across levels (Supplementary Table, in osf.io). This is a form of mathematical coupling. Ideally, the authors would seek primary phenotypes that are as uniquely genetically determined as possible, as opposed to trait mixtures as ECC.*

We appreciate the reviewer's perspective on the mathematical relationship between the phenotypes. These four phenotypes were selected with careful consideration of both biological relevance and data stability. In the methods section titled "Spinal cord IDP and covariate selection" we detail our decision to exclude metrics such as spinal cord angle, length, and orientation because they lacked the necessary repeatability and biological interpretability for this study. Our final selection of AP/RL diameters, CSA, and ECC was instead driven by rigorous repeatability analysis and Principal Component Analysis tests to ensure we utilised information that is consistently measurable.

While CSA and ECC are indeed composite measurements, we maintain that they yield unique biological information that individual linear dimensions cannot capture. Much like the use of Body Mass Index or waist-to-hip ratio in systemic health research, composite metrics often provide a meaningful summary of morphology beyond mathematical coupling. CSA is a standard metric that allows for direct comparison with existing literature, while ECC captures the specific shape of the spinal cord, a trait previously shown to be altered in movement disorder patient but remained stable over time (Rezende et al., 2023, 2024). This shape-based information is not directly interpretable by looking at diameters or CSA alone, as those metrics primarily reflect volume and size rather than the shape of the spinal cord.

The inclusion of ECC is further supported by its exceptional stability. Our analysis of repeat scans, including those taken up to seven years apart, showed that ECC measurements for C1–C3 achieved remarkably high correlations ($r = 0.82–0.91$, Figure S4). This suggests that ECC is a highly stable trait that may be congenital and less impacted by transient environmental factors than other metrics. This stability was reflected in our genetic results, where ECC yielded the highest number of GWAS signals and the highest heritability estimates across our analysis. We believe the shape of the spinal cord may be influenced by the surrounding vertebral structures, and we have added a section to the Discussion commenting on these phenotypic differences and the value of including shape-based metrics in genetic discovery: "Interestingly, we found that eccentricity, a metric reflecting circular or flattened shape, emerged as the most genetically robust metric, yielding the highest heritability and the greatest number of independent GWAS signals…The longitudinal stability of eccentricity measure suggests it captures a highly conserved aspect of spinal cord morphology, likely reflecting congenital structural variations that are less susceptible to environmental influences."

5. *The authors are commended for their investigations with MS and neuropathies, but these are known to affect the spinal cord. Sure, spinal cord could be a biomarker, but they may reflect more a consequence of the disease than a cause. I'm not sure that the "biomarker" interpretation is a valuable one, or even the best interpretation. Biomarker for risk? Biomarker for disease progression? That said, studying genetic influences on the spinal cord is a valuable end on its own, with or without such disease biomarker interpretation, which is a weak one.*

We agree that the term "biomarker" may imply a stronger causal role than our current data supports, as spinal cord alterations can be a consequence rather than a primary driver of disease. We have revised the title of the manuscript to use the term "associations" instead of "biomarker". The extensive scope of the study, including the associations with other brain phenotypes and health outcomes were intended not only for novel discovery but also as a crucial validation step to ensure our imaging-derived phenotypes yield sensible and robust findings across different types of analyses. While the link between the spinal cord and conditions such as multiple sclerosis and neuropathies is well-documented, a systematic analysis of the overall health impact across the broader phenome has not been previously examined at this scale. Our objective is to advocate for greater research attention to this region of the central nervous system, emphasising that it is not an isolated structure but one that demonstrates links to a wide range of health outcomes. We hope this work encourages future research to consider the spinal cord's broader clinical relevance more comprehensively.

6. *Stil on this, causal mechanisms pervade the discussion, e.g., "these findings suggest that spinal cord morphology may reflect broader systemic health and disease susceptibility." (p.10), "results suggest potential shared genetic architecture between spinal cord shape metrics and a range of disease categories" (p.11, at the top). Yet, the authors themselves admit that "Thus, our results do not provide robust evidence of a causal effect of PD or BMI on spinal cord phenotypes" (p.11, at the bottom). The overall story of the paper isn't well tied within a consistent interpretation.*

We thank the reviewer for pointing out the lack of consistency in our discussion of causal mechanisms. Our use of five Mendelian Randomization methods as sensitivity analyses led to mixed signal. We have now revised the Discussion to provide a single, consistent interpretation as: "While

we observed evidence of genetic overlap across several conditions, MR analyses did not support robust causal effects."

7. *Perhaps I'm missing among the various files submitted and table in the osf.io website but do the authors report SNPs differentially associated with spinal cord traits with and without height as a confound? It matters as both sets of variants are of interest: those that affect spinal cord possibly also impacting height, and those that are uniquely associated with the spinal cord itself with no impact on height. Supplementary Table 5 (Excel file) shows the correlations between the two analyses, but that isn't interesting. The variants themselves are.*

Thank you for this insightful comment. We agree that distinguishing SNPs associated with spinal cord traits with and without adjustment for height is important for assessing whether associations are independent of overall body size. In response, we have included a new supplementary table (Supplementary Table 5) reporting genome-wide significant independent loci from the GWAS without height adjustment for spinal cord shape metrics. We also performed a direct comparison with the height-adjusted analysis to identify loci that are shared between analyses or specific to either analysis.

We have updated the Results section to summarise the overlap between analyses with and without height adjustment and to highlight loci that are shared or specific to the non-height-adjusted analysis.
"For sensitivity analysis, we performed GWAS without adjusting for height (Fig. S16–19, Table S3). We identified 182 independent genome-wide significant variants ($P < 4.2 \times 10^{-9}$), mapping to 104 unique genomic loci (Table S5). Most loci (100/104) were consistently identified in both height-adjusted and non-adjusted analyses, indicating robust associations independent of height. However, three loci (rs5861819, rs2956033, rs10883922) were significant only in the height-adjusted analysis, while four loci (rs58273475, rs112635299, rs2410978, rs4128399) were significant only in the non-adjusted analysis, suggesting that a small subset of loci may reflect height-mediated or height-independent effects..."

8. Some comments in the discussion are highly speculative, e.g., role of kidneys, synaptic homeostasis, etc. The fact, is, we don't know and won't know until the pathways involving these genes are elucidated one by one. As long as we keep studying statistical associations, we won't learn much more (I blame the damage caused by Systems Biology across multiple basic sciences, but I digress...).

We acknowledge that systems biology findings require downstream validation and cannot establish causality, a limitation inherent to GWAS-based approaches more broadly. Nevertheless, these analyses remain invaluable for identifying genomic regions harbouring genetic signals that influence spinal cord morphology, generating testable hypotheses that can be pursued through functional genetics approaches. We hope this work provides a robust foundation for spinal cord genetics and encourages the field to direct greater attention toward this region in the CNS.

All and all, this is an interesting study, timely given the data availability. The authors have clearly put a lot of effort in it, e.g., by performing multi-ancestry replication (even if likely underpowered,

but there are no other datasets like UKB), by stratifying by sex, and by going further than just doing a GWAS (colocalization, gene prioritization, PheWAS, MR, etc). They also make the code available on Github, which is a plus. I believe my critiques above are addressable, except the inherent issues related using brain MRI to assess non-brain tissue. Perhaps the uncertainty in the more caudal segments can be addressed by modifications in the deep-learning method for segmentation, which is the topic of their other manuscript.

REVIEWER COMMENTS

We thank both reviewers for their considered and useful comments. A point-by-point response is provided below, with the reviewers' original comments in italics.

Reviewer #2 (Remarks to the Author):

The authors have made useful revisions in response to my previous comments, including clarifying the pipeline's analytical rationale, explaining the choice of Pearson over Spearman correlations, and adding biological interpretation for key candidate genes. These revisions improve the manuscript's transparency. Several points warrant further consideration to strengthen the paper.

The rationale for the gene-set enrichment analysis still needs clarification. The authors state that gene-set enrichment was not applied to genes derived from the other five methods because many alternative pathway analyses rely on hypergeometric tests that may show p-value inflation due to gene length, gene density, co-expression, and LD. However, these factors mainly affect the upstream probability that a gene is selected by a given gene-prioritization method, whereas the hypergeometric test itself simply evaluates overlap between a gene list and a background set. Therefore, the authors should clarify whether their concern is that the other five methods produce heterogeneous outputs that are not suitable for a unified pathway enrichment framework, or whether there are specific statistical limitations that prevent pathway analysis based on those gene sets.

We thank the reviewer for this point and agree that our original wording was imprecise. Our concern is primarily statistical, although we note the methods also differ in their testable gene universes and therefore produce heterogeneous outputs that are not suitable for a unified pathway enrichment analysis. To clarify our original response, we would argue that gene selection for each of our methods will be influenced in part by the factors outlined by the reviewer (i.e., gene length, SNP density, cis-eQTL discoverability, and LD/physical clustering), which violates the assumption of equal selection underlying standard hypergeometric tests and are also correlated with biological pathway annotations. In addition, the six methods we employed test different sets of genes (13,729-18,504 of 28,140), meaning that neither the union nor intersection provides an appropriate common background. We therefore used GSA-MiXeR, which avoids thresholded gene lists and explicitly accounts for factors such as gene size and density. Although over-representation analysis could be performed using a matched, permutation-calibrated background, we believe this would largely reproduce the corrections incorporated in the GSA-MiXeR approach.

We have added a brief justification of GSA-MiXeR for gene-set enrichment analysis in methods:

> We used GSA-MiXeR rather than gene-set analysis of the convergent gene list, to avoid biases arising from gene selection by the individual gene-based tests (such gene length, SNP density and cis-eQTL discoverability, which are properties also correlated

with pathway annotation) and because the tests share no common background appropriate for over-representation testing.

There are also several minor issues in the gene-set enrichment section. The authors report both 432 unique significant genes and 434 genes significantly associated with a spinal cord phenotype in at least one approach; this discrepancy should be checked and corrected. For the 37 overlapping genes, it would be clearer to specify whether the overlap was calculated using the 96 high-confidence genes, the 218 genes supported by at least two approaches, or the full 432/434-gene set. If the overlap is intended only as a descriptive comparison, this should be stated clearly; otherwise, additional statistical support would be needed to interpret it as evidence of enrichment or convergence beyond chance. In addition, the phrase "organ development pathways" is broad; since the response specifically mentions kidney and renal development pathways, the revised text should use more precise wording.

We thank the reviewer for their attention to detail and noticing the small discrepancy in the number of significant gene-based associations across methods. We found the cause of this discrepancy was due to a mismatch between gene symbols from different gene-based tests. For example, previously unmapped symbols were retained as distinct genes and collapsed to a single gene and undercounted in the union. To fix this oversight, the six gene-based tests were aligned to a common gene reference (GENCODE v47) before aggregation. This alignment resulted in a slightly different number of unique significant genes (N=442 rather than 432/434). The number of genes implicated by at least 2 and 3 gene-based approaches remains 218 and 96, respectively. We want to emphasize the results of each gene-based test remain unchanged, and our description of key genes (e.g.,*SGTB*, *TGFA*, and *ROR2*) in both results and discussion remain valid.

We have updated the relevant results section with tracked changes and pasted in full below:

> Candidate risk genes for each of the four morphological features of cervical spinal cord segments C1–C3 were prioritized using six gene-based mapping approaches. Positional mapping was conducted with MAGMA and mBAT-combo, while functional mapping leveraged eQTL data via TWAS (PsychENCODE and GTEx v8 expression weights) and SMR (eQTLGen and BrainMeta datasets). Across all spinal cord morphology-segment combinations, we identified 442 unique significant genes after applying a Bonferroni correction (P < 0.05 after adjusting for all tests within each gene-based method and phenotype; for the multi-tissue TWAS and SMR panels the denominator is the number of gene-by-tissue tests, and the most significant tissue per gene was selected only after correction). The largest number of unique gene associations was found for eccentricity (*n* = 241), followed by AP diameter (*n* = 194), CSA (*n* = 165), and RL diameter (*n* = 113). The proximity-based approaches mBAT-combo and MAGMA identified the largest number of significant genes, with 292 and 196 genes, respectively, followed by TWAS of GTEx tissues with 128 significant

gene associations (Table S14-17). Of the 442 genes significantly associated with a spinal cord phenotype in at least one approach, 218 were implicated by at least two approaches and 96 by at least 3 approaches (Table S18-19). Five genes—*DPYSL4*, *CCDC88B*, *UQCC1*, *UFSP1* and *DDHD2*—were significant in all six approaches.

Among the 96 genes identified by at least three mapping approaches, gene candidates for spinal cord phenotypes were further prioritized using colocalization (TWAS-COLOC) and SMR-HEIDI analyses (Fig. 4a; Table S14-17, Fig. S35). Colocalization identified TWAS associations supported by a shared causal variant between GWAS and eQTL signals (posterior probability H4 > 0.8). HEIDI was used to retain SMR associations consistent with a single causal variant influencing both gene expression and trait (P > 0.05). Of the 96 genes implicated by at least three gene-based tests, 44 had a significant TWAS association in GTEx brain tissue and showed evidence of colocalization (i.e., PP.H4 > 0.8), while 41 genes had a significant SMR association in brain tissue, 29 of which had a HEIDI *P* value > 0.05. Top among the list of high confidence genes was *SGTB,* which was implicated by five gene-based approaches across three morphological measures and showed evidence of colocalization in human brain tissue. Other high confidence genes included *TGFA* and *ROR2*, both of which were implicated by five gene-based approaches (across three spinal levels) and colocalized in human brain tissue (Fig. 4a). Although *TGFA* fell just outside the top 20 when genes were ranked jointly across all features and tests (rank 24 of 1,761 genes), it was among the most highly ranked genes for individual features—8th for RL diameter and 17th for CSA—and is nonetheless highlighted here as a candidate of interest (Supplementary Figure 35). These candidate genes have important implications in diverse functions such as neuronal survival and neurite morphology[45], neural tissue proliferation during development[46], and axial skeletal and vertebral patterning[47].

For transparency, we have also added a description of how the gene-based tests were aggregated in the methods section:

Aggregation of gene-based tests

Results from the six gene-based tests were aligned to a common gene reference (GENCODE v47) before aggregation. Multiple-testing correction was applied within each gene-based test and spinal cord phenotype separately. For the multi-tissue panels (FUSION TWAS across 13 GTEx v8 brain tissues, and the SMR panels) multiple testing correction was done on the number of gene-by-tissue tests, and the most

significant tissue per gene was selected only after correction, so tissue selection does not inflate significance. Both Benjamini-Hochberg FDR and Bonferroni-adjusted thresholds of 0.05 were computed. Genes were then aggregated across the 12 phenotypes: a test was counted as implicating a gene if that gene reached the threshold for any of the four morphological measures at any cervical level. Similarly, per-measure counts implicated a gene if it was significant at any of C1-C3. Convergence was then quantified as the number of tests implicating each gene at Bonferroni significance. Genes passing the significance thresholds were further priortised using the TWAS colocalisation filter (PP.H4>0.8) and the SMR HEIDI test (P>0.05).

We have also updated Figure 4 and all Supplementary Tables 14-20 (now Supplementary Data 14-30) related to the gene-based tests. To improve transparency and confidence in each intersection, the new Supplementary Data include cross-tabulations of significant genes across gene-based tests and spinal cord morphology phenotypes. Furthermore, within the Supplementary Data, we have highlighted numbers in yellow that are quoted in the main body of the manuscript. We once again thank the reviewer for spotting this error in the aggregation of gene-based results.

Reviewer #3 (Remarks to the Author):

I co-reviewed this manuscript with one of the reviewers who provided the listed reports. This is part of the Nature Communications initiative to facilitate training in peer review and to provide appropriate recognition for Early Career Researchers who co-review manuscripts.

Reviewer #4 (Remarks to the Author):

I'd like to thank the authors for their responsive revision. The bulk of my concerns have been addressed.

I have one remaining minor point, which I believe is easily resolvable: The "biomarker" framing has been toned down, yet "potential radiological biomarker" survives in the Discussion and "potent as a biomarker" in the Limitations. A natural question still arises on biomarker for what? There are some elements in the ms that allude to "a broad range of disease states", after mentions to "viral infections", "musculoskeletal disorders", "gout arthropathies", and "essential hypertension". But if said biomarker is that unspecific, how helpful is it? Addressing this is easy: studying genetic influences on the spinal cord is valuable on its own, regardless of whether there is a "biomarker" interpretation for something or not. In fact, I see no need to mention the idea of biomarkers at all.

We thank the reviewer for acknolwedge that studying the genetic architecture of the spinal cord is a valuable contribution in its own right, independent of any biomarker interpretation. On reflection, we recognise that our health-outcome analyses were comprehensive in scope, extending well beyond the core GWAS findings. This was inspired by the approach taken in prior neuroimaging genetics studies of subcortical brain structures (e.g., García-Marín et al.), where a similarly broad phenotypic and genetic correlation screen was used to characterise a structure's relevance across health domains, rather than to establish a specific clinical biomarker. Our intent was similar, to explore whether the spinal cord shows a similar or distinct pattern of health associations to well-studied brain structures, and in doing so, to motivate future, more targeted research rather than to claim diagnostic or predictive utility.

We have therefore removed all references to "biomarker" from the Discussion and Limitations, including the phrases "potential radiological biomarker" and "potent as a biomarker," and have revised the surrounding text to frame these findings as motivation for future research into the spinal cord's role in systemic health, rather than as evidence of clinical biomarker utility.
